# Harmine Alleviated Sepsis-Induced Cardiac Dysfunction by Modulating Macrophage Polarization *via* the STAT/MAPK/NF-κB Pathway

**DOI:** 10.3389/fcell.2021.792257

**Published:** 2022-01-17

**Authors:** Weibin Ruan, Xinyun Ji, Yating Qin, Xinxin Zhang, Xiaoning Wan, Chuanmeng Zhu, Chao Lv, Chongqing Hu, Juan Zhou, Li Lu, Xiaomei Guo

**Affiliations:** ^1^ Department of Cardiology, Tongji Hospital, Tongji Medical College, Huazhong University of Science and Technology, Wuhan, China; ^2^ Department of Cardiology, Hubei Provincial Hospital of Traditional Chinese Medicine, Wuhan, China; ^3^ Department of Cardiology, Renmin Hospital of Wuhan University, Wuhan, China

**Keywords:** harmine, sepsis, cardiac dysfunction, macrophage polarization, STATs, MAPKs, NF-κB

## Abstract

Sepsis is a dysregulated systemic inflammatory response that often leads to cardiac dysfunction, which is termed sepsis-induced cardiomyopathy (SIC). Harmine, a natural *β*-carboline alkaloid compound, has been shown to exert pharmacological effects on several diseases. Here, we investigated whether harmine protected against SIC development and the underlying mechanisms. *In vitro*, the expression of the M1 phenotype markers iNOS and COX-2 was increased in RAW 264.7 cells stimulated with lipopolysaccharide (LPS), but this effect was reversed by the harmine intervention. Furthermore, LPS-induced increases in the levels of inflammatory cytokines, including IL-1*β*, IL-6, TNF-*α*, iNOS, COX-2, PGE2 and TXB2, generated by macrophages were suppressed when the cells were pretreated with harmine. Meanwhile, our findings showed that harmine administration effectively attenuated inflammation and apoptosis in H9c2 cells in the proinflammatory environment produced by macrophages, as evidenced by reductions in NLRP3 and cleaved caspase 3 levels and the p-NF-κB/NF-κB ratio. The western blot results indicated that the mechanisms underlying harmine-mediated inhibition of M1 polarization might be associated with suppression of STAT1/3, NF-κB and MAPK activation. Furthermore, an LPS injection induced cardiac dysfunction and decreased the survival rate of mice, which were alleviated by harmine treatment, and the relevant mechanism was possibly attributed to a drug-induced attenuation of the inflammatory and apoptotic processes in cardiomyocytes. Collectively, these results implied that harmine treatment protected against SIC by suppressing M1 phenotypic polarization and inflammation in macrophages.

## Introduction

Sepsis is a systemic inflammatory response syndrome caused by various dysregulated responses to host infections, accompanied by life-threatening multiorgan dysfunction (Lelubre and Vincent, 2018). Among the injuries or dysfunctions involved in the process of sepsis, cardiac injury and malfunction often occur and are associated with the pathogenesis of hemodynamic disturbance, which is also defined as sepsis-induced cardiomyopathy (SIC) (Hollenberg and Singer, 2021). To date, numerous researchers have attempted to decipher the mechanisms of SIC, and inflammation-related macrophages have been shown to play important roles in SIC development (Li et al., 2021). Macrophages are characterized by highly heterogeneous and plastic immunocyte subtypes, and phenotypic changes in macrophages in a specific environment and time are referred to as macrophage polarization (Murray, 2017). M1 macrophages exhibit a proinflammatory phenotype related to the host defense against pathogens, while M2 macrophages possess an anti-inflammatory phenotype related to inflammation resolution and tissue repair (Liu et al., 2014). When exposed to diverse stimuli, such as LPS, TNF-*α* and IFN-*γ*, M1 macrophages are activated, and intracellular inflammatory and metabolic reprogramming pathways are activated, including STAT1, NF-κB and HIF-1*α* axes, eventually promoting the generation of plentiful cytokines that damage cardiomyocytes and then induce cardiac dysfunction (Y. Liu et al., 2017; Chen et al., 2021). Moreover, other bioactive factors, such as PGE2, TXB2 (Amunugama et al., 2021), NO (Jia et al., 2018) and NLRP3 (Busch et al., 2021), are reported to facilitate inflammation progression and tissue injury during the course of sepsis. However, to date, the specific mechanisms responsible for SIC remain elusive.

Harmine, which is mainly derived from the seeds of the medicinal plant *Peganum harmala L.* (Li et al., 2017; Sharifi-Rad et al., 2021), is a natural *β*-carboline alkaloid compound with numerous pharmacological functions, including anti-inflammatory, neuroprotective (Li et al., 2018), and antiviral functions (Benzekri et al., 2018), as well as the regulation of cellular apoptosis and proliferation (Zhu et al., 2021). For instance, harmine exerts inhibitory effects on LPS-induced inflammatory reactions in the kidney by suppressing the NF-κB and NLRP3 pathways, accompanied by a reduction in TNF-*α*, IL-6 and IL-1*β* levels (Niu et al., 2019). Additionally, harmine inhibits diabetes by regulating PPAR-γ activity and promoting adipogenesis in adipocytes (Waki et al., 2007). It also accelerates human pancreatic beta cell proliferation by weakening the activation of dual-specificity tyrosine-regulated kinase-1a (Wang et al., 2015). In terms of the cardiovascular system, harmine represents an effective therapeutic agent for cardiac hypertrophy (Huang et al., 2021) and atherosclerosis (Yang et al., 2021). Nevertheless, researchers do not completely understand whether harmine exerts regulatory effects on antagonizing SIC progression. As the inflammatory response is strongly implicated in the pathogenic processes of SIC, in this study, we investigated the effects of harmine on the development of SIC and subsequently explored the potential molecular mechanisms.

## Materials and Methods

### Chemicals and Antibodies

Harmine was purchased from Med Chem Express (#442-51-3, MCE, Shanghai, China), and the purity of this compound was >99%. LPS (O111:B4, #L2630) was purchased from Sigma–Aldrich (St. Louis, MO, United States). Primary antibodies against NLRP3 (1:1000, #15101S), NF-κB (1:1000, #8242S), p-NF-κB (1:1000, #3033S), JNK (1:1000, #9252S), p-JNK (1:1000, #4668S), ERK (1:1000, #4695S), p-ERK (1:1000, #4370S), P38 (1:1000, #9212S), p-P38 (1:1000, #4511S), iNOS (1:1000, #13120S), and cleaved caspase 3 (1:1000, #9664S) were acquired from Cell Signaling Technology (Danvers, MA, United States). Primary antibodies against STAT1 (1:1000, #A0027), p-STAT1 (1:1000, #AP0054), STAT3 (1:1000, #A1192), p-STAT3 (1:1000, #AP0705), IκB*α* (1:1000, #A11168), and p-IκB*α* (1:1000, #AP1220) were acquired from ABclonal (Wuhan, China). Anti-COX-2 (1:1000, #12375-1-AP) and anti-GAPDH (1:5000, #60004-1-lg) antibodies were purchased from Proteintech (Wuhan, China). The secondary anti-mouse and anti-rabbit antibodies were purchased from Cell Signaling Technology (Danvers, MA, United States). Cell Counting Kit 8 (WST-8/CCK-8) was obtained from Meilun (Dalian, China). Dihydroethidium (DHE) reagents and a TUNEL apoptosis kit were purchased from Beyotime (Shanghai, China). The nuclear dye DAPI applied in cellular and histological staining was obtained from BOSTER (Wuhan, China). All other chemicals or reagents applied in this study were of analytical grade.

### Study Design and Animal Experiment

Eight- to ten-week-old C57BL/6J male mice weighing 22–25 g were purchased from Beijing Vital River Laboratory Animal Technology Co., Ltd. (Beijing, China) and housed in a specific pathogen-free (SPF) environment with a temperature of 23–25°C and humidity of 55 ± 5% with free access to food and water. All animal experiments were approved by the Animal Research Committee of Tongji Medical College and conducted in accordance with the animal welfare procedures mentioned in ARRIVE and NIH guidelines. Briefly, forty mice were adapted to the environment for 1 week and then randomly divided into four groups (*n* = 10 mice per group): 1) Control, 2) harmine alone, 3) LPS alone, and 4) LPS + harmine. Harmine was suspended in 0.5% CMC-Na (sodium carboxymethylcellulose), and then ultrasound was applied for 10 minutes to dissolve and acquire a better suspension. Mice in the harmine group received 50 mg/kg harmine by gavage daily for seven consecutive days, while mice in the remaining groups received an equal volume of the 0.5% CMC-Na solution. The dosage of harmine used here was based on a previous study (Niu et al., 2019). At the end of Day 7 and 1 h after the last harmine or vehicle solution gavage, mice in the LPS group were intraperitoneally injected with LPS (10 mg/kg, dissolved in sterile normal saline) to induce septic cardiomyopathy (Li et al., 2019), while mice in the other groups received an equal volume of normal saline.

Another forty mice were also subjected to the same procedure as mentioned above, except that the dosage of LPS was 25 mg/kg to reach a lethal dosage (Jia et al., 2018), to investigate survival. After the LPS injection, the survival of the mice was observed every 12 h up to 96 h.

### Echocardiogram Analysis

Twelve hours after the LPS injection, the mice were anesthetized with 5% isoflurane and subjected to transthoracic two-dimensional (2-D)-guided M-mode echocardiography (Vevo3100, FUJIFILM VisualSonics, Toronto, ON, Canada) with a 30 MHz phased array transducer. The short axis of left ventricular M-mode images was analyzed using Vevo 3100 Imaging software. The calculation of the ejection fraction (EF) was in accordance with the left ventricular end-systolic diameter (LVEDs) and left ventricular end-diastolic diameter (LVEDd). LV end diastolic dimensions (EDDs) and end systolic dimensions (ESDs) were recorded. Fractional shortening (FS) was calculated as [(EDD−ESD)/EDD] ×100. The cardiac parameters were calculated in five consecutive cardiac cycles.

After the echocardiography analysis, mice were sacrificed with sodium pentobarbital (200 mg/kg). The heart tissues were removed, placed in liquid nitrogen, and then transferred to -80°C until further experiments.

### Drug Pretreatment and Cell Culture

Harmine was dissolved in DMSO (MP Biology, United Kingdom) at a concentration of 50 mmol/L and then aliquotted and stored at −80°C until further dilution. LPS was dissolved in sterile PBS at a final concentration of 1 mg/ml and subsequently aliquotted and stored at −80°C. H9c2 cells and RAW 264.7 cells (ATCC, United States) were cultured in Gibco high-glucose DMEM (Thermo Fisher, United States) containing 10% (v/v) Gibco fetal bovine serum (Thermo Fisher, United States), 100 IU/ml penicillin and 100 μg/ml streptomycin (Solarbio, Beijing, China) at 37°C in a humidified incubator with 5% CO_2_. The medium was changed every other day during the culture period. When the cells reached 80% confluence, they were seeded in 6-well plates for protein collection, in 12-well plates for RNA extraction and immunofluorescence staining, and in 96-well plates for the CCK-8 assay.

### Cell Viability and Cell Treatment

For the CCK-8 assay, RAW 264.7 cells were seeded in 96-well plates and cultured overnight. The next day, harmine was diluted with medium to different concentrations of 1, 5, 10, 25, and 50 μmol/L and added to the cells. At the indicated time points, the medium was replaced with a mixture of CCK-8 solution and medium, followed by an incubation for 2 h according to the manufacturer’s instructions. The OD values were read at 450 nm using a microplate reader (Thermo Fisher, United States). For the protein and RNA analyses, RAW 264.7 cells were treated with LPS (1 μg/ml) for different times (1, 3, 6, 12, and 24 h). Furthermore, they were pretreated with different concentrations of harmine (1, 5, 10, 25 μmol/L) or an equal volume of vehicle (DMSO) for 1 h and then incubated in the presence or absence of LPS (1 μg/ml) for 12 h. At the indicated time points, they were subjected to protein or RNA extraction.

### Immunofluorescence Staining

For immunofluorescence (IF) staining, RAW 264.7 cells were seeded in 12-well plates containing coverslips. Cells were pretreated with harmine (25 μmol/L) or an equal volume of vehicle for 1 h and then incubated in the presence or absence of LPS (1 μg/ml) for 12 h. Cells were washed with PBS twice, fixed with 4% paraformaldehyde for 20 min, and then permeabilized with 0.2% Triton X-100 for 15 min. Subsequently, 10% goat serum (BOSTER, Wuhan, China) was added to block nonspecific binding for 1 h at 37°C. After blocking, the cells on coverslips were incubated with an antibody against iNOS (1:200, CST, United States) overnight at 4°C. The next day, Alexa Fluor® 488-conjugated goat anti-rabbit IgG (H + L) (1:200, Servicebio, Wuhan, China) and DAPI (1:200, BOSTER, Wuhan, China) were used to label target proteins and nuclei, respectively. Finally, the coverslips were gently mounted on glass slides using anti-fluorescence quenching agent. The expression of target proteins was observed using an Olympus BX51 fluorescence microscope. The images were captured and analyzed using Image-Pro Plus 6.0 software (Maryland, United States).

### Treatment of H9c2 Cells With Macrophage Conditioned Medium

RAW 264.7 cells were seeded in six-well plates, pretreated with 1, 5, 10, or 25 μmol/L harmine for 1 h and then stimulated with or without LPS (1 μg/ml) for 12 h. After the challenge, the medium (conditioned medium) from the macrophages was collected and centrifuged. Subsequently, the conditioned medium was applied in two assays: 1) Detection of cytokines and other inflammatory mediators (as described in detail below) and 2) the medium was removed from H9c2 cells, and the H9c2 cells were washed with PBS and then cultured with conditioned medium for another 12 h. At the end of the experiment, cardiomyocyte lysates were prepared for western blotting. The method was based on a previous study (J. Chen et al., 2018).

### Detection of Inflammatory Mediators

Enzyme-linked immunosorbent assay (ELISA) was applied to detect the levels of the cytokines IL-6, TNF-*α*, PGE2 and TXB2 in conditioned medium collected from RAW 264.7 cells. Nitric oxide (NO) levels were measured using a biochemical kit based on the Griess reaction.

Animal blood samples were collected in 1.5 ml heparinized EP tubes, centrifuged at 3000 × g for 10 min at 4°C and subsequently stored at −80°C until further analyses. Animal plasma was used to detect biomarkers of cardiac damage, including LDH and cTn-I, and inflammatory cytokines, including IL-1*β*, IL-6, TNF-*α*, NO, MCP-1 and HMGB1. The LDH kit was purchased from Jiancheng (Nanjing, China), while the remaining inflammatory mediators were detected using commercial enzyme-linked immunosorbent assay (ELISA) or biochemical kits obtained from Jingmei Biological Technology Co. Ltd. (Jiangsu, China) according to the manufacturer’s instructions.

### Western Blotting and Quantitative RT–PCR

The heart tissue and collected cells were lysed with RIPA buffer (BOSTER, Wuhan, China), and the protein concentration was detected using a BCA kit (BOSTER, Wuhan, China) according to the manufacturer’s instructions. Briefly, the protein was loaded into 8–12% SDS–PAGE gels, separated, and then transferred to 0.45 μm PVDF membranes (Millipore, Billerica, MA, United States). The membranes were blocked with 5% BSA at room temperature for 1 h and incubated with primary antibodies overnight at 4°C with gentle agitation. The next day, the membranes were washed with TBS containing 0.1% Tween-20 (TBST) and then probed with the corresponding HRP-conjugated secondary antibodies for 1 h at room temperature. Subsequently, the membranes were washed with TBST and exposed to reagents from a chemiluminescence ECL kit (BOSTER, Wuhan, China) according to the manufacturer’s instructions. The protein signal was detected using an automatic chemiluminescence imaging system (Tanon 5200, Guangzhou, China). The images were analyzed with ImageJ software, and the protein expression was normalized to the GAPDH level.

Total RNA was extracted using TRIzol reagent (RNA iso Plus, #108-95-2, Takara, Beijing, China) and reverse transcribed to cDNAs with an ABScript II cDNA Fist-Strand Synthesis Kit (#RK20402, ABclonal, Wuhan, China). The target genes were amplified using 2X Universal SYBR Green Fast qPCR Mix (#RK21203, ABclonal, Wuhan, China). The mRNA expression was normalized to the GAPDH level and calculated using the ΔΔCT method. The primers used in this study were as follows: mouse IL-1*β*, forward: 5’-TTT​GAA​GTT​GAC​GGA​CCC​C-3’, reverse: 5’-TGT​GCT​GCT​GCG​AGA​TTT​G-3’; mouse COX-2, forward: 5’-CAT​CCC​CTT​CCT​GCG​AAG​TT-3’, reverse: 5’-CAT​GGG​AGT​TGG​GCA​GTC​AT-3’; mouse TNF-*α*, forward: 5’-ACT​GAA​CTT​CGG​GGT​GAT​CGG​T-3’, reverse: 5’-TGG​TTT​GCT​ACG​ACG​TGG​GCT​A-3’; mouse IL-6, forward: 5’-GGA​GCC​CAC​CAA​GAA​CGA​TAG-3’, reverse: 5’-GTG​AAG​TAG​GGA​AGG​CCG​TG-3’; and mouse GAPDH, forward: 5’-TGT​GAA​CGG​ATT​TGG​CCG​TA-3’, reverse: 5’-GAT​GGG​CTT​CCC​GTT​GAT​GA-3’.

### Histological Assays

The mouse hearts were harvested, rinsed with phosphate-buffered saline (PBS), and fixed with 4% paraformaldehyde (PFA) for 24 h. They were dehydrated, embedded in paraffin and then sectioned into 5 μm slices. Hematoxylin and eosin (H&E) staining was performed to assess the infiltration of immune cells and the cardiomyocyte size and arrangement. The H&E-stained sections were observed using a normal light BX53 Olympus microscope. Additionally, one part of the heart tissue was embedded in OCT embedding agent and subsequently cut into 8-μm-thick sections using a constant temperature freezing microtome. The frozen sections were stored at −20°C until further staining.

DHE staining was performed to detect reactive oxygen species (ROS) in the heart tissue. Briefly, the frozen sections were rinsed with PBS twice for 10 min each. Then, a tissue pen was used to draw a circle around the heart. DHE reagent (10 μmol/L) was added to the circle and incubated with the sections at 37°C for 30 min in the dark. After the incubation, these sections were washed with PBS three times for 5 min each. Next, DAPI was added to the tissue and incubated for 10 min at room temperature. Finally, these sections were washed with PBS three times for 5 min each and then shaken dry and sealed with anti-fluorescence quenching sealant. Images of DHE-stained sections were observed and captured using an Olympus BX51 fluorescence microscope.

A terminal deoxynucleotidyl transferase-mediated dUTP nick end labeling (TUNEL) staining kit was applied to assess apoptosis in heart tissues. Briefly, circles were drawn around frozen slices with a tissue pen, and then 4% paraformaldehyde was added to fix the heart tissue and incubated for 20 min at room temperature. The slices were then washed with PBS three times for 5 min each. The TUNEL reagent was added to the slices and incubated at 37°C for 1 h in the dark. After the incubation, the slices were washed with PBS three times for 5 min each. Finally, the slices were shaken dry and sealed with anti-fluorescence quenching reagent (Beyotime, Shanghai, China) containing DAPI. TUNEL staining was captured using an Olympus BX51 fluorescence microscope. These images were analyzed with Image-Pro Plus 6.0 software.

### Statistical Analyses

All data were analyzed using GraphPad Prism 8 software (San Diego, CA, United States). The data are presented as means ± SEM. The Kaplan-Meier method was applied to assess survival followed by the log rank test. Differences between two groups were compared using two-tailed Student’s t test. Multiple comparisons among different groups were performed using one-way analysis of variance (ANOVA), followed by Tukey’s post hoc test. *p* values ≤ 0.05 were considered significant.

## Results

### Harmine Attenuated LPS-Induced M1 Polarization of RAW 264.7 Cells in a Dose-dependent Manner

**FIGURE 1:**
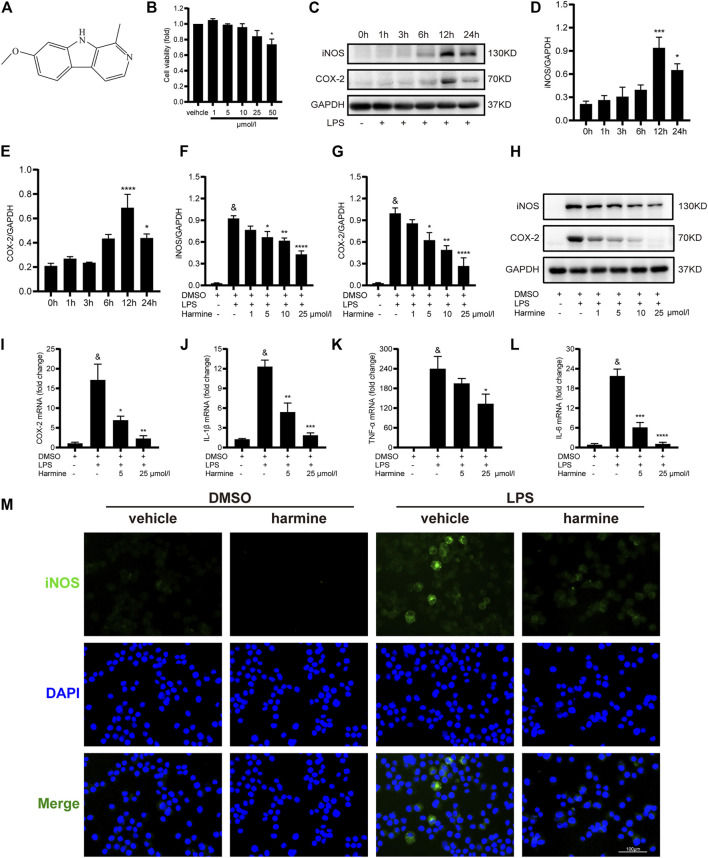
Harmine inhibited M1 macrophage polarization in a dose-dependent manner. **(A)** Chemical structure of harmine. **(B)** Viability of RAW 264.7 cells after harmine treatment for 12 h (*n* = 3 independent experiments with six replicates). **(C–E)** Expression of the iNOS (*n* = 5) and COX-2 proteins detected (*n* = 4) after LPS stimulation for different periods (1, 3, 6, 12, or 24 h). **(F–H)** Expression of the iNOS (*n* = 4) and COX-2 proteins (*n* = 4). RAW 264.7 cells were pretreated with harmine (1, 5, 10, or 25 μmol/L) for 1 h and then challenged with LPS (1 μg/ml) for 12 h **(I–L)** The relative expression of the COX-2, TNF-*α*, IL-1*β*, and IL-6 mRNAs. RAW 264.7 cells were pretreated with harmine (5 and 25 μmol/L) for 1 h and then stimulated with LPS (1 μg/ml) for 12 h. **(M)** Immunofluorescence staining for iNOS (green) and DAPI (blue) (*n* = 3, 200X, scale bar: 100 μm). RAW 264.7 cells were pretreated with harmine (25 μmol/L) for 1 h and then stimulated with LPS (1 μg/ml) for 12 h. GAPDH was applied as an internal control for western blotting and quantitative RT–PCR. Data are shown as the means ± SEM, ^&^ *p* < 0.05 compared with the control group; *p < 0.05, **p < 0.01, ***p < 0.001, and ****p < 0.0001 compared with the LPS group.

The chemical structure of harmine is shown in Figure 1A. First, we explored the optimal concentration of harmine by performing CCK-8 assay, and we found that a concentration less than 50 μmol/L was nontoxic to RAW 264.7 cells (Figure 1B). Furthermore, RAW 264.7 cells were treated with LPS (1 µg/ml) for different times of 1, 3, 6, 12, and 24 h to determine the optimal time of the LPS intervention. The expression levels of the M1 macrophage phenotype markers iNOS and COX-2 increased gradually and peaked at 12 h, as shown in Figures 1C–E. Hence, we chose 12 h as the optimal intervention time for subsequent experiments. Then, RAW 264.7 cells were pretreated with different doses of harmine (1, 5, 10, or 25 μmol/L) for 1 h and stimulated with or without LPS (1 μg/ml) for 12 h to explore the anti-inflammatory effects of harmine. The expression of iNOS and COX-2 was increased in LPS-stimulated cells, and these changes were inhibited by harmine in a dose-dependent manner (Figures 1F–H). Then, the COX-2, IL-6, TNF-*α* and IL-1*β* mRNA levels were investigated, and we observed that the expression of these genes was also downregulated by different doses (5 and 25 μmol/L) of harmine compared to the LPS-challenged groups (Figures 1I–L). Furthermore, the immunofluorescence staining showed that the LPS-induced increase in iNOS expression in RAW 264.7 cells was obviously ameliorated by harmine administration (Figure 1M). Overall, these findings suggested that harmine inhibited macrophage polarization to the M1 proinflammatory phenotype in a dose-dependent manner.

### Harmine Mitigated Inflammation in RAW 264.7 Cells and Conditioned Medium-Treated H9c2 Cells

**FIGURE 2:**
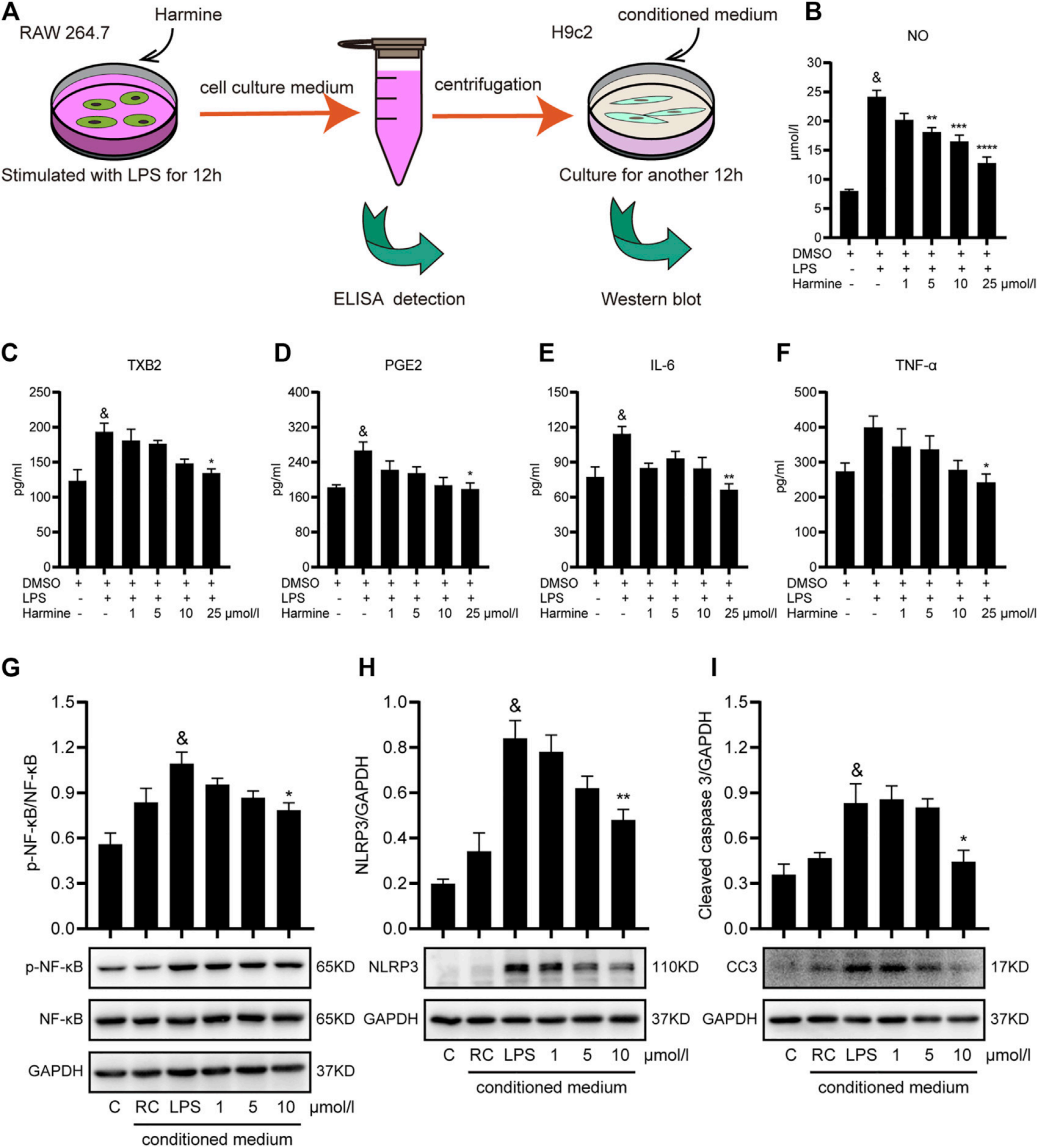
Harmine attenuated inflammation in RAW 264.7 cells and conditioned medium-treated H9c2 cells. **(A)** Diagram of conditioned medium treatment of H9c2 cells. RAW 264.7 cells were pretreated with harmine (5 and 25 μmol/L) for 1 h and then stimulated with LPS (1 μg/ml) for 12 h. The medium was collected in EP tubes and then centrifuged. The supernatants were subjected to ELISAs, and harmine-conditioned medium (1, 5, or 10 μmol/L) was subsequently incubated with H9c2 cells for another 12 h. H9c2 cell lysates were collected for western blotting after culture. **(B–F)** The levels of the following inflammatory mediators in the supernatants collected from RAW 264.7 cells were detected using Griess reaction or ELISAs: NO, TXB2, PGE2, IL6, and TNF-*α* (*n* = 4). **(G–I)** Levels of the p-NF-κB (*n* = 5), NF-κB (*n* = 5), NLRP3 (*n* = 4), and cleaved caspase 3 (*n* = 4) proteins in conditioned medium-treated H9c2 cells. Conditioned medium from RAW 264.7 cells: RAW control group (RC), 1, 5, and 10 μmol/L. GAPDH was used as the internal control for western blotting. Data are presented as the means ± SEM, ^&^ *p* < 0.05 compared with the control group; *p < 0.05, **p < 0.01, ***p < 0.001, and ****p < 0.0001 compared with the LPS group.

Then, we explored whether harmine exerted protective effects on inflammation in macrophages and cardiomyocytes using a conditioned medium-treated model (Figure 2A). RAW 264.7 cells were pretreated with different doses of harmine and then challenged with LPS for 12 h. Subsequently, the medium of these cells was centrifuged. The levels of inflammatory mediators in the supernatants were detected using ELISAs or the Griess reaction. As expected, the concentrations of NO, IL-6, TNF-*α*, PGE2, and TXB2 in the culture supernatants were increased in the LPS-stimulated group. Harmine treatment significantly decreased the levels of these inflammatory cytokines in a dose-dependent manner (Figures 2B–F). The conditioned medium was transferred to H9c2 cells and incubated for 12 h to further investigate the beneficial effects on harmine on cardiomyocyte survival. Based on our data, harmine effectively improved the inflammation-related apoptosis of cardiomyocytes insulted by conditioned medium, as evidenced by the reduction in NLRP3 and caspase 3 expression and the p-NF-κB/NF-κB ratio (Figures 2G–I).

### The Protective Effects of Harmine Were Associated With the Suppression of STAT1/3/MAPK/NF-κB Cascades

**FIGURE 3:**
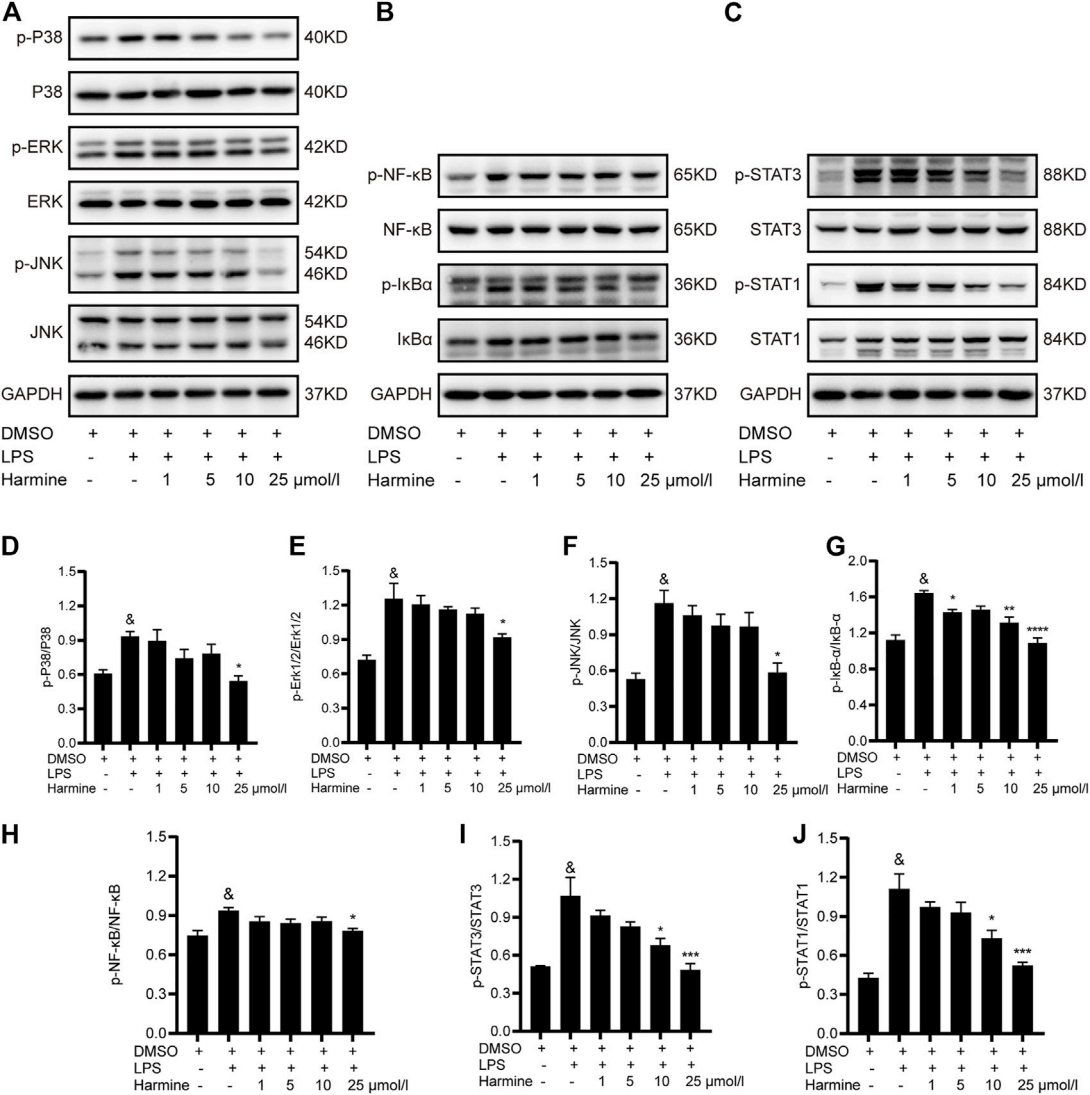
Harmine inhibited M1 macrophage polarization through the STAT1/3, MAPK, and NF-κB pathways. RAW 264.7 cells were pretreated with harmine (1, 5, 10, or 25 μmol/L) for 1 h and then stimulated with LPS (1 μg/ml) for 12 h **(A,D–F)** Levels of proteins in MAPK pathways: p-P38 (*n* = 4), P38 (*n* = 4), JNK (*n* = 3), p-JNK (*n* = 3), Erk1/2 (*n* = 3), and p-Erk1/2 (*n* = 3), **(B–H)** Levels of proteins in NF-κB pathways: p-NF-κB (*n* = 8), NF-κB (*n* = 8), p-IκB-*α* (*n* = 4), and IκB-*α* (*n* = 4), **(C–J)** Levels of STAT1/3 proteins: p-STAT3 (*n* = 4), STAT3 (*n* = 4), p-STAT1 (*n* = 4), and STAT1 (*n* = 4). GAPDH was used as internal control. Data are presented as the means ± SEM, ^&^ *p* < 0.05 compared with the control group; *p < 0.05, **p < 0.01, ***p < 0.001, and ****p < 0.0001 compared with the LPS group.

RAW 264.7 cells were pretreated with different doses of harmine and then stimulated with LPS to investigate the mechanism by which harmine attenuated inflammatory reactions in macrophages. Three pathways involved in M1 macrophage polarization and inflammation development were detected. We found that the levels of phosphorylated P38, JNK and ERK1/2 were increased in cells stimulated with LPS, and the harmine intervention decreased the activities of MAPK family proteins (Figures 3A,D–F), especially at a concentration of 25 μmol/L, in a dose-dependent manner. Moreover, the expression of IκB*α* and NF-κB and the levels of their corresponding phosphorylated forms were measured. Increased levels of p-IκB*α* and p-NF-κB were observed in cells exposed to LPS, which were reversed by harmine treatment (Figures 3B,G,H). Additionally, the LPS-evoked activation of STAT1/3 in macrophages was significantly attenuated by harmine administration (Figures 3C,I,J). Collectively, harmine was likely to suppress M1 polarization and inflammatory processes in macrophages by regulating STAT1/3/NF-κB/MAPK signal transduction.

### Harmine Ameliorated Sepsis-Induced Cardiac Injury and Improved the Survival Rate

**FIGURE 4:**
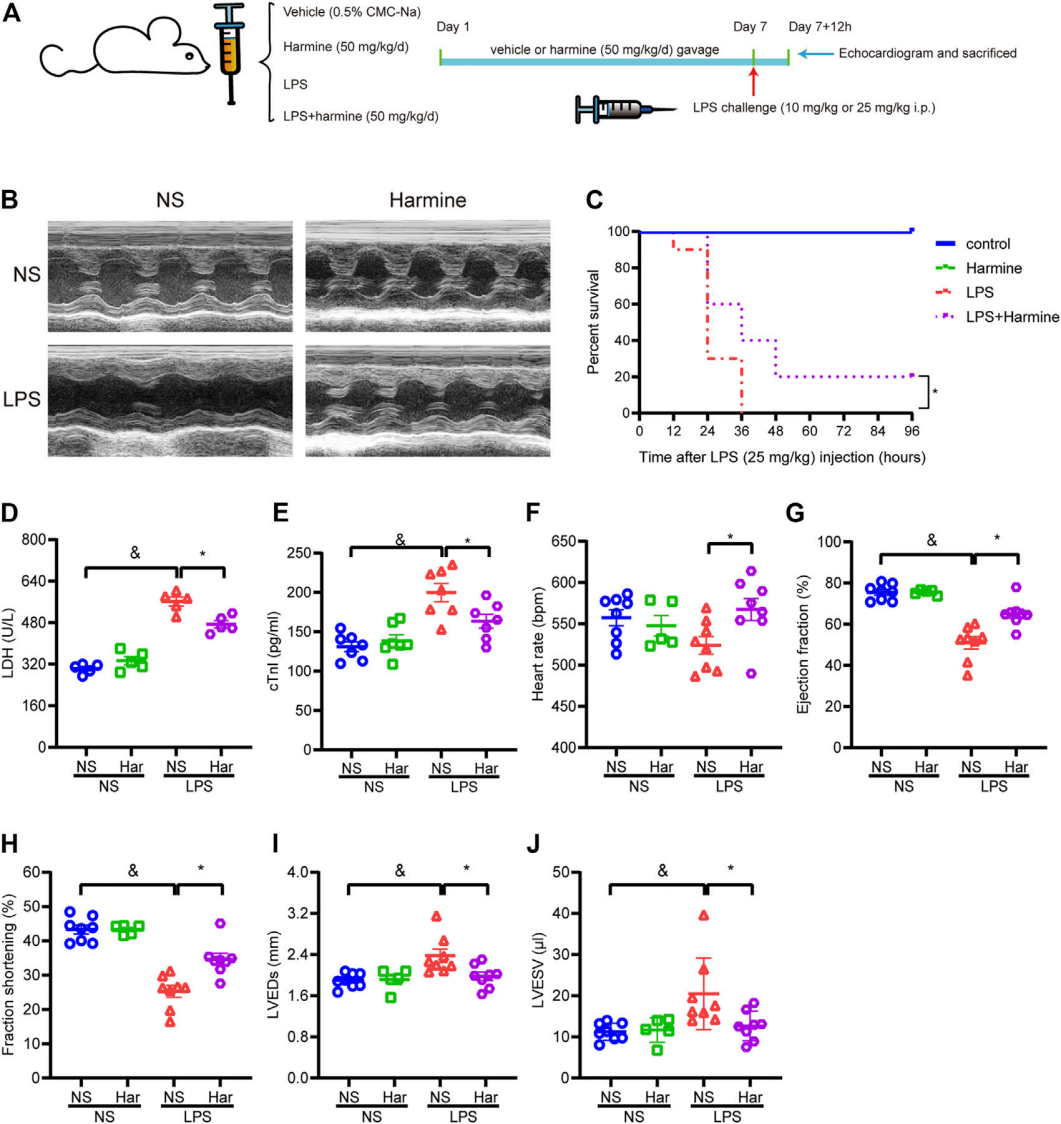
Harmine mitigated sepsis-induced cardiac dysfunction and increased survival. **(A)** Diagram of the animal experiment. Harmine (50 mg/kg) or an equal volume of a 0.5% CMC-Na solution was administered to mice daily for seven consecutive days. At the end of Day 7 and 1 h after the gavage of harmine or vehicle, mice were intraperitoneally injected with LPS (10 mg/kg) or an equal amount of normal saline (NS). After 12 h, the mice underwent echocardiograms and were then sacrificed for further experiments. **(B)** Representative images of M-mode echocardiograms from mice. NS = normal saline. **(C)** Survival of mice after LPS (25 mg/kg) or vehicle injection. Mouse survival was recorded every 12 h up to 96 h **(D,E)** Plasma levels of LDH (*n* = 5) and cTnI (*n* = 7). **(F)** Heart rate, **(G)** ejection fraction (EF), **(H)** fractional shortening (FS), **(I)** left ventricular end-systolic diameter (LVEDs), and **(J)** left ventricular end-systolic volume (LVESV). [**(F–J)**, *n* = 5 mice in the harmine group and *n* = 8 mice in the remaining groups]. Data are presented as the means ± SEM, ^&^ *p* < 0.05 compared with the control group; *p < 0.05 compared with the LPS group.

Here, we further investigated the roles of harmine in modulating the cardiac function of mice with sepsis-related inflammation. The SIC animal model was produced via an intraperitoneal injection of LPS (Figure 4A), and we observed that mice injected with LPS exhibited somnolence, diarrhea, reduced motor activity, ruffled fur, and ocular exudates, as previously reported (Luo et al., 2020). Echocardiography was performed to evaluate the cardiac function of mice. Septic mice exhibited symptoms of SIC, including a defective ejection fraction (EF) and decreased fractional shortening (FS); however, the harmine treatment significantly improved the EF and FS values compared to those of the LPS group, as indicated in Figures 4B,G–H. Additionally, the LPS-stimulated increase in the left ventricular end-systolic dimension (LVEDs) and left ventricular end-systolic volume (LVESV) and decrease in the heart rate were normalized by harmine treatment (Figures 4F,I,J). Notably, harmine slightly increased the stroke volume and cardiac output compared to those of the LPS cohort (data not shown). The levels of the myocardial damage biomarkers LDH and cTnI were also increased after the LPS injection, while harmine overtly mitigated LPS-evoked cardiac injury, as evidenced by reductions in LDH and cTnI levels (Figures 4D,E).

Regarding survival, after the injection of a lethal dose of LPS (25 mg/kg), the survival rate of mice was recorded every 12 h up to 96 h. The number of deaths in the LPS cohort increased sharply in the first 24 h, while harmine treatment slowed mortality. At the end of the survival experiment, some mice still survived in the harmine treatment group, while none of the mice in the LPS cohort survived at 36 h. Thus, harmine treatment apparently improved the survival rates of mice with LPS-elicited sepsis (Figure 4C).

### Harmine Inhibited LPS-Induced Myocardial Disarrangement, Oxidative Stress, and Cellular Apoptosis

**FIGURE 5:**
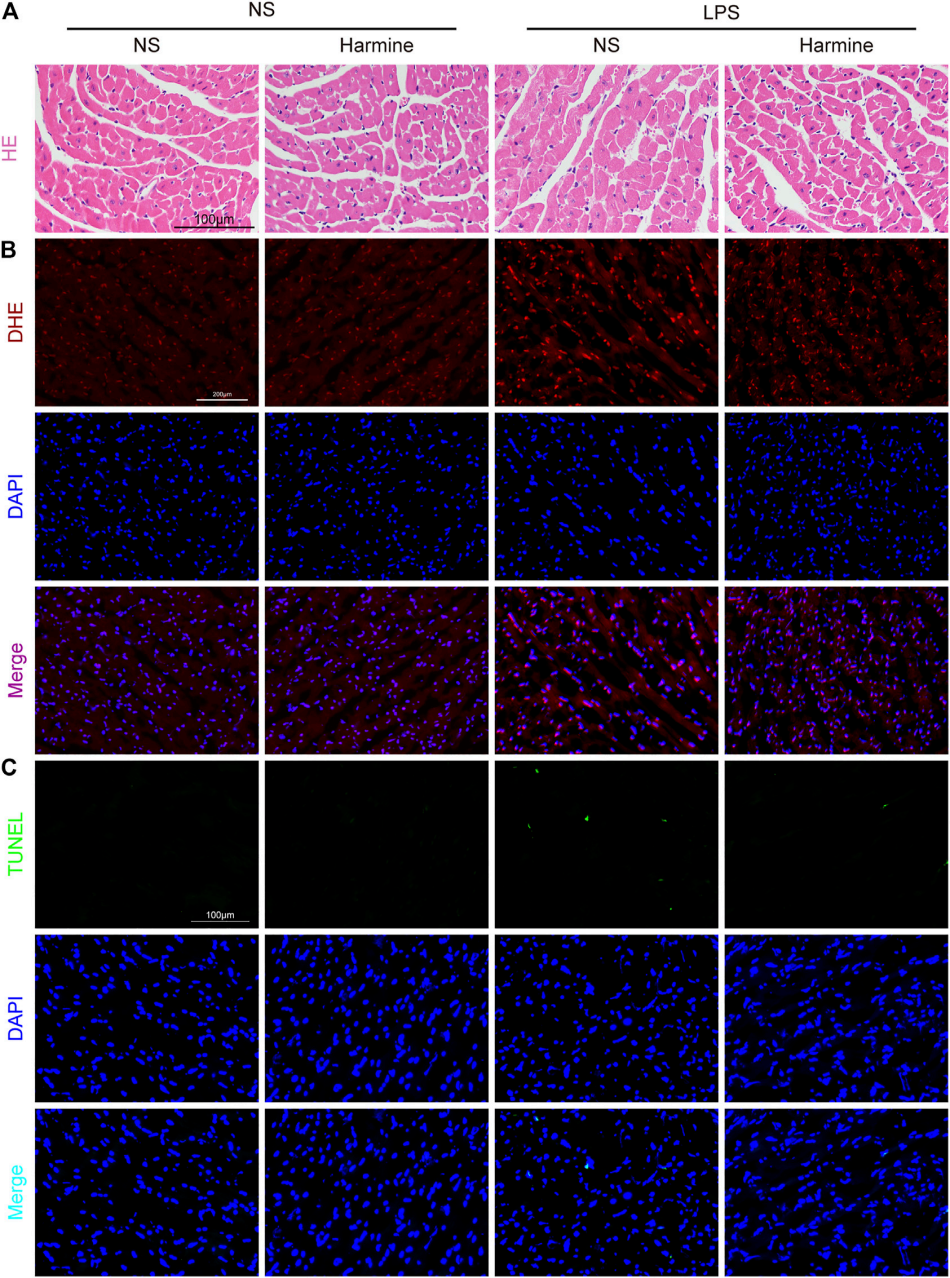
Harmine restrained sepsis-induced cardiac disarrangement, oxidative stress and apoptosis. **(A)** Representative images of H&E staining in the myocardium (400X, scale bar: 100 μm). **(B)** Representative images of DHE staining (200X, scale bar: 200 μm). DHE (red) and DAPI (blue). **(C)** Representative images of TUNEL staining in the myocardium (200X, scale bar: 100 μm). TUNEL (green) and DAPI (blue).

As shown by H&E staining (Figure 5A), the myocardial fibers were disarranged, and cardiomyocyte swelling was observed in LPS-challenged mice. Harmine treatment seemed to restore the myocardial arrangement and normalized the cardiomyocyte size in mice. Furthermore, the oxidative stress triggered by LPS in the heart tissue was detected using DHE staining. The red fluorescence intensity of cardiac samples in the LPS group was stronger, but harmine significantly decreased the fluorescence intensity (Figure 5B). Regarding myocardial apoptosis, the harmine intervention effectively alleviated the LPS-induced increase in the number of TUNEL-positive cells in the myocardium (Figure 5C). The quantitative data of TUNEL positive cells was shown in Supplementary Presentation 7. Collectively, the histopathological data indicated that harmine weakened LPS-evoked cardiac dysfunction at least partially by ameliorating edema, oxidative stress and apoptosis in the heart tissue.

### Harmine Alleviated Systemic and Cardiac Inflammation in Mice Challenged With LPS *in vivo*

**FIGURE 6:**
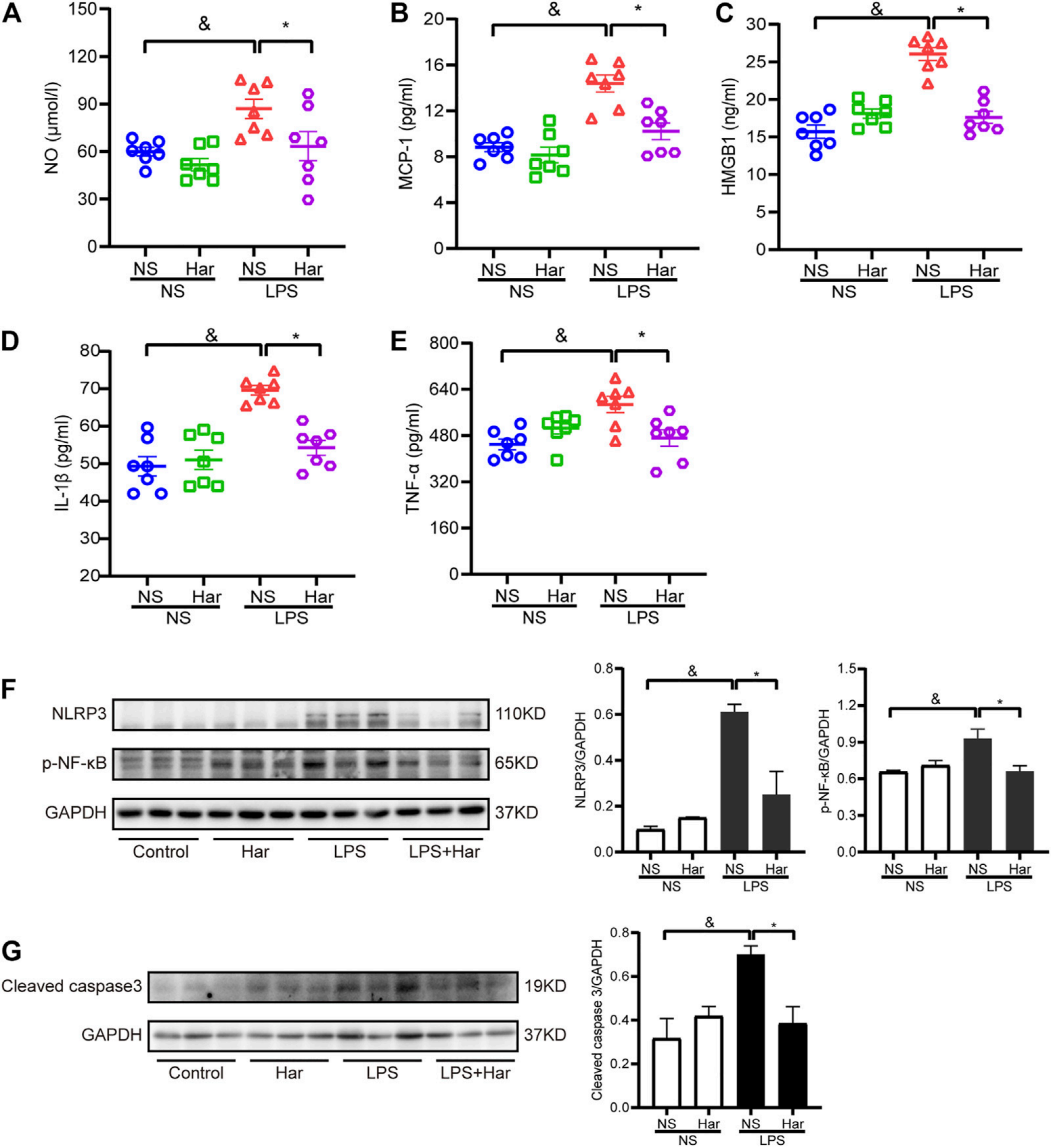
Harmine suppressed sepsis-induced systemic and cardiac inflammation. Levels of the following inflammatory mediators were detected in mouse plasma: **(A)** Nitric oxide (NO), **(B)** MCP-1, **(C)** HMGB1, **(D)** IL-1*β*, and **(E)** TNF-*α*. [**(A–E)**, *n* = 7 mice per group]. **(F)** Levels of the NLRP3 (*n* = 3) and p-NF-κB (*n* = 3) proteins. **(G)** Levels of the cleaved caspase 3 protein (*n* = 3). GAPDH was used as internal control for western blotting. Data are presented as the means ± SEM, ^&^ *p* < 0.05 compared with the control group; *p < 0.05 compared with the LPS group.

Then, the potential mechanisms underlying the cardioprotective effects of harmine were investigated. First, the levels of circulating inflammatory factors, including NO, IL-1*β*, TNF-*α*, MCP-1 and HMGB1, were detected, and the levels of these biomarkers were increased in LPS-challenged mice but were reduced by harmine treatment (Figures 6A–E). Moreover, the levels of phosphorylated NF-κB in LPS-stimulated heart tissue were increased compared to those in the control group, while harmine significantly inhibited the increased phosphorylation of these signaling molecules. In addition, the levels of NLRP3 and the proapoptotic protein cleaved caspase 3 were also increased in the cardiac tissue of LPS-challenged mice, but harmine treatment significantly decreased the levels of NLRP3 and cleaved caspase 3 (Figures 6F,G).

## Discussion

Numerous lines of evidence have established that SIC-related cardiac dysfunction is associated with increased mortality in patients with sepsis (Merx and Weber, 2007). The underlying mechanisms of SIC are intricate, and inflammatory reactions represent pivotal factors contributing to SIC initiation and progression (Martin et al., 2019). To date, the therapeutic efficacy and safety of treatments for SIC are limited in terms of clinical applications (Cavaillon et al., 2020; Lin et al., 2020). Harmine is a natural *β*-carboline alkaloid monomer mainly extracted from *P. harmala L.* that exerts potent protective effects on several diseases, such as diabetes and tumors (Machado et al., 2020). In this study, we observed that the harmine intervention effectively alleviated cardiac dysfunction triggered by LPS-related sepsis in mice, and the relevant mechanism was attributed to drug-induced inhibition of macrophage polarization to the M1 phenotype and subsequent inflammation.

Macrophages in a resting stage acquire the M1 phenotype upon stimulation with proinflammatory substances, accompanied by the activation of intracellular signaling pathways and an increase in the expression of downstream inflammatory cytokines (Porta et al., 2015). First, our findings revealed that the contents of iNOS and COX-2, which are key biomarkers of M1 macrophages, were increased following LPS insult, but these changes were abrogated by the administration of harmine in a dose-dependent manner, suggesting that harmine was capable of restraining the M1 phenotypic conversion of macrophages exposed to proinflammatory stimuli. TNF-*α* and interleukins exert crucial effects on the pathogenic processes of inflammatory responses by activating signaling networks and aggravating tissue damage. High levels of NO, which is generated by iNOS in activated M1 macrophages, are transformed to peroxynitrite by reacting with superoxide, leading to the exacerbation of oxidative stress (Carlstrom, 2021). PGE2 and TXB2 are derived from the bioconversion of polyunsaturated fatty acids and arachidonic acid through COX-2-mediated catalysis (Alhouayek and Muccioli, 2014). Previous studies have determined the positive effects of NO, PGE2 and TXB2 on facilitating inflammation progression, and reductions in the contents of these factors were proven to be effective at ameliorating sepsis-induced inflammatory reactions (Bitto et al., 2012; I. Chen et al., 2018; Jia et al., 2018; Xie et al., 2021). Then, we found that increased levels of IL-6, TNF-*α*, NO, PGE2, and TXB2 produced by RAW 264.7 cells exposed to LPS were reversed by harmine treatment, implying potent drug-related anti-inflammatory actions. Furthermore, we explored the effects of LPS-induced macrophage inflammation on cardiomyocyte damage. The signaling molecules phosphorylated NF-κB and NLRP3 were activated and the level of the apoptosis-related protein cleaved caspase 3 was increased in myocardial cells exposed to inflammatory stimuli, but these changes were ameliorated by the harmine intervention, suggesting that harmine might increase cardiomyocyte survival by inhibiting the proinflammatory signaling pathways activated by exogenous stimuli.

The MAPK signaling molecules ERK1/2, P38 and JNK participate in several cellular physiological processes, such as inflammation, apoptosis, proliferation and differentiation (Karin and Chang, 2001; Cargnello and Roux, 2011; Arthur and Ley, 2013). Recently, some studies revealed that these bioactive factors are intimately involved in regulating macrophage polarization to the M1 phenotype (Zhang et al., 2019; Qu et al., 2021). Additionally, compelling evidence has revealed that NF-κB not only acts as an important mediator responsible for the transcription of inflammatory substances but also plays favorable roles in facilitating the transformation of resting macrophages into activated M1 macrophages (Lv et al., 2020). Moreover, another group of proinflammatory transcription factors, STATs, were reported to participate in initiating the polarization to the inflammation-promoting phenotype (Wang et al., 2020). Because M1 phenotypic transformation is required for inflammation to occur in macrophages, we investigated the activities of signaling cascades implicated in the M1 phenotypic polarization of macrophages. Our findings revealed that LPS stimulation significantly increased the activities of ERK1/2, P38, JNK, NF-κB and STAT1/3 in RAW 264.7 cells, but harmine administration suppressed the activation of the aforementioned signaling intermediates in a dose-dependent manner. Based on these data, the inhibitory effects of harmine on macrophage polarization to the M1 phenotype were possibly ascribed to the suppression of signaling pathways involving MAPKs, NF-κB and STATs, laying the foundation for its anti-inflammatory actions.

Accumulating data indicate that sepsis is commonly accompanied by multiorgan impairments, particularly SIC that is characterized by cardiac dysfunction (Boissier et al., 2017), which is the leading cause of death worldwide (Rudd et al., 2020). Previous studies have confirmed the therapeutic effects of harmine on endotoxemia-induced acute kidney and lung injuries (X. Liu et al., 2017; Niu et al., 2019). LPS was intraperitoneally injected into mice to mimic sepsis-induced myocardial dysfunction and further evaluate the protective effects of harmine on SIC development *in vivo*. We observed that the LPS intervention induced a hyperinflammatory state in mice, as evidenced by increased levels of NO, IL-1*β*, TNF-*α*, MCP-1 and HMGB1 in the circulation, while harmine gavage obviously reduced the concentrations of these cytokines.

Meanwhile, harmine effectively reversed the decreased EF, FS and survival rate. Additionally, it reversed the increased LVEDs, LVESV, LDH, and cTnI levels, suggesting that harmine administration helped relieve cardiac structural and functional disorders, thereby improving the recovery and survival of mice. Disorganized myocardial fibers, edema, and cardiomyocyte death are recognized as the main histological features of cardiac dysfunction. In this study, histopathological analyses revealed that the LPS-induced myocardial disarrangement and apoptosis were significantly alleviated by harmine treatment, highlighting its cardioprotective activities. Emerging evidence supports the hypothesis that circulating macrophages infiltrate heart tissues and exacerbate local inflammatory reactions in the septic environment as the vital pathogenic mechanism contributing to SIC development (Jia et al., 2018; Wang et al., 2021). Our findings showed that the activity of phosphorylated NF-κB/NLRP3 was increased in the myocardium of mice injected with LPS, but the harmine gavage abrogated the activation of this inflammation-promoting signaling axis, accompanied by the suppression of downstream caspase 3 activity responsible for cellular apoptosis, implying that harmine was likely to weaken inflammatory signal transduction to alleviate cell death in the myocardium of mice with SIC, ultimately improving the abnormalities in parameters reflecting cardiac function.

**FIGURE 7:**
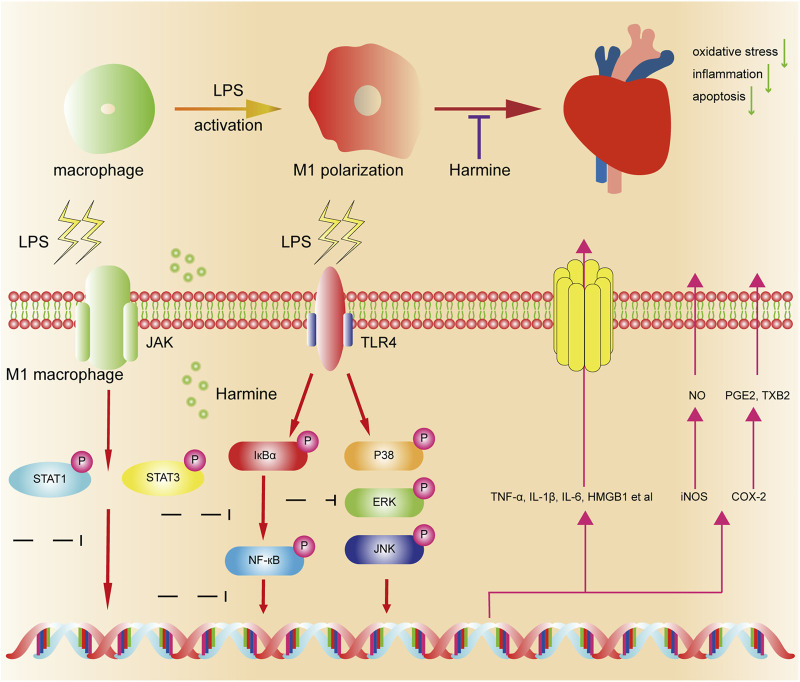
Schematic diagram of the proposed mechanism underlying the protective effects of harmine on sepsis-induced cardiac dysfunction. Macrophages are activated by LPS and produce abundant inflammatory mediators (proinflammatory M1 phenotype). Harmine inhibits M1 macrophage polarization by suppressing the STAT1/3, MAPK, and NF-κB pathways and subsequently reduces the release of inflammatory mediators. Ultimately, it alleviates cardiac oxidative stress, inflammation and apoptosis *in vitro* and *in vivo*.

In summary, we, for the first time, observed that harmine treatment exerted protective effects on sepsis-induced cardiac damage and functional disorders. Using LPS-stimulated cells and mice as the research model, our results indicated that the cardioprotective mechanisms of harmine might be associated with the suppression of macrophage polarization to the M1 phenotype, subsequent retardation of inflammation development and attenuation of cardiomyocyte apoptosis (Figures 7). This study provided a reliable preclinical basis for harmine as a potential therapeutic candidate in the management of SIC.

## Data Availability

The original contributions presented in the study are included in the article/Supplementary Material, further inquiries can be directed to the corresponding authors.
