# Supplementary material for: Harmine Alleviated Sepsis-Induced Cardiac Dysfunction by Modulating Macrophage Polarization via the STAT/MAPK/NF-κB Pathway
Source: Front Cell Dev Biol. 2022 Jan 17;9:792257. doi: 10.3389/fcell.2021.792257 (PMC8801946; doi:10.3389/fcell.2021.792257)
Supplement: Supplementary file 1 [file Presentation1.PPTX]

## Slide 1
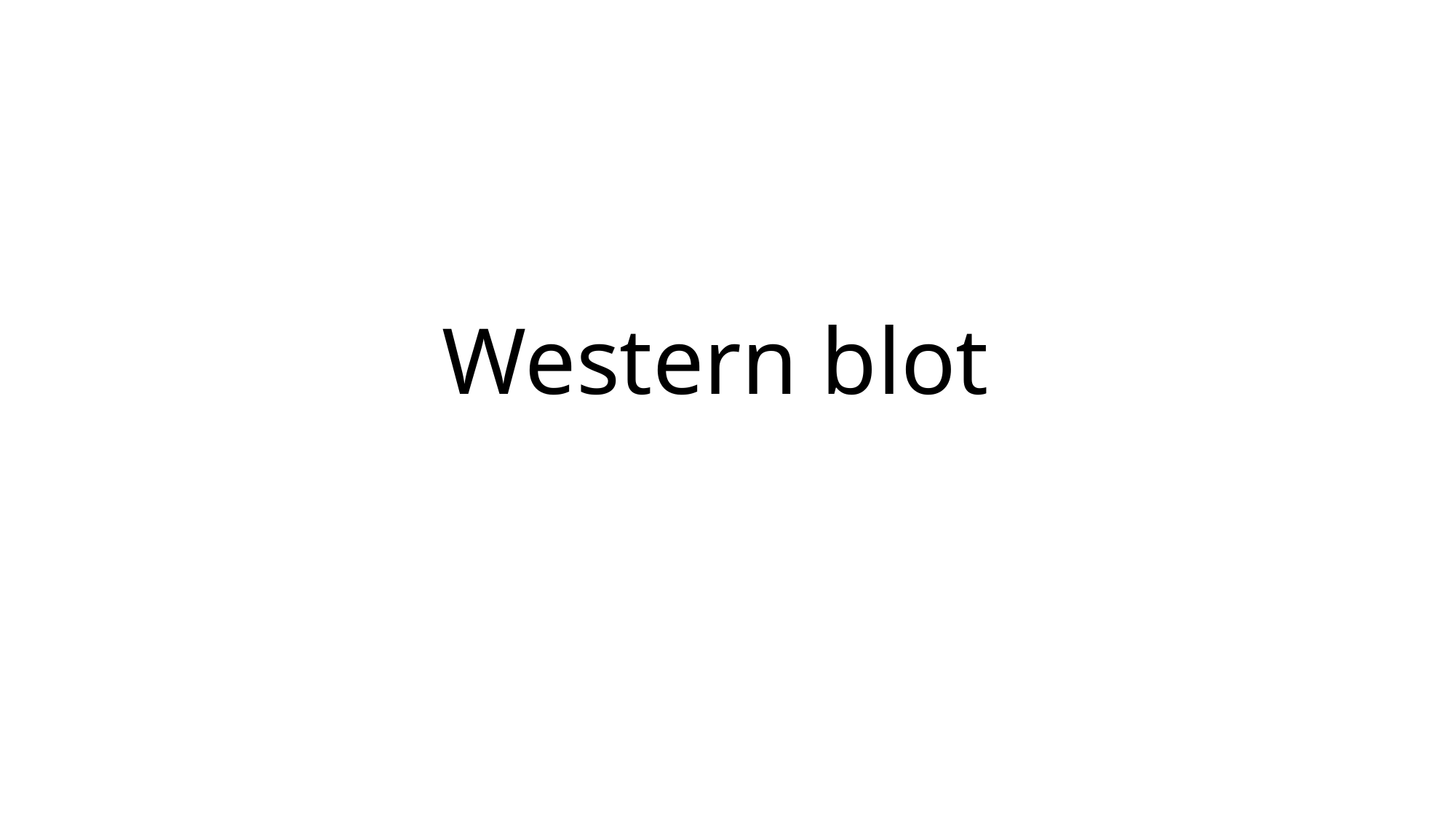

# Western blot

## Slide 2
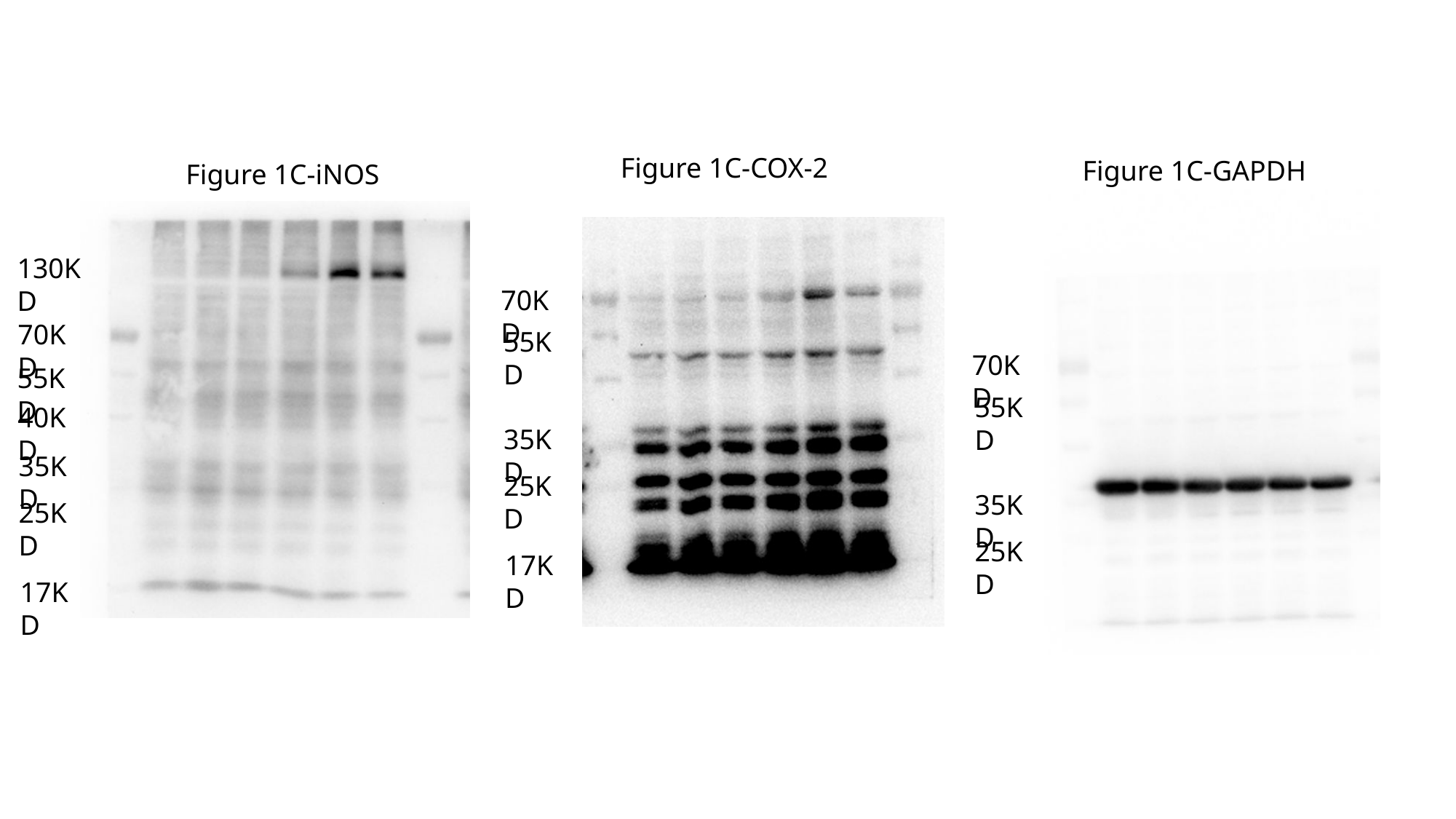

Figure 1C-COX-2
Figure 1C-GAPDH
Figure 1C-iNOS
130KD
70KD
70KD
55KD
70KD
55KD
55KD
40KD
35KD
35KD
25KD
35KD
25KD
25KD
17KD
17KD

## Slide 3
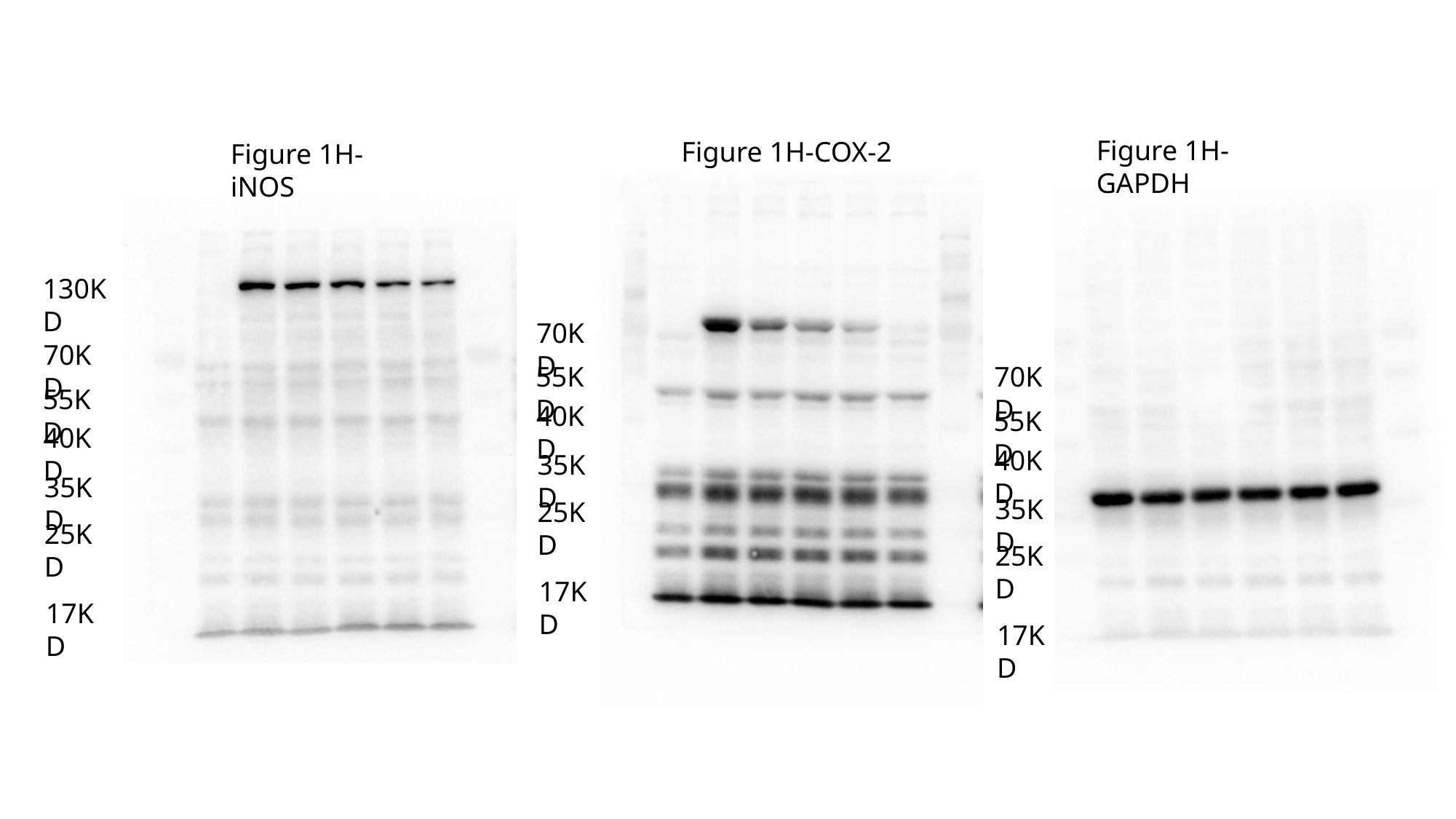

Figure 1H-GAPDH
Figure 1H-COX-2
Figure 1H-iNOS
130KD
70KD
70KD
55KD
70KD
55KD
40KD
55KD
40KD
40KD
35KD
35KD
35KD
25KD
25KD
25KD
17KD
17KD
17KD

## Slide 4
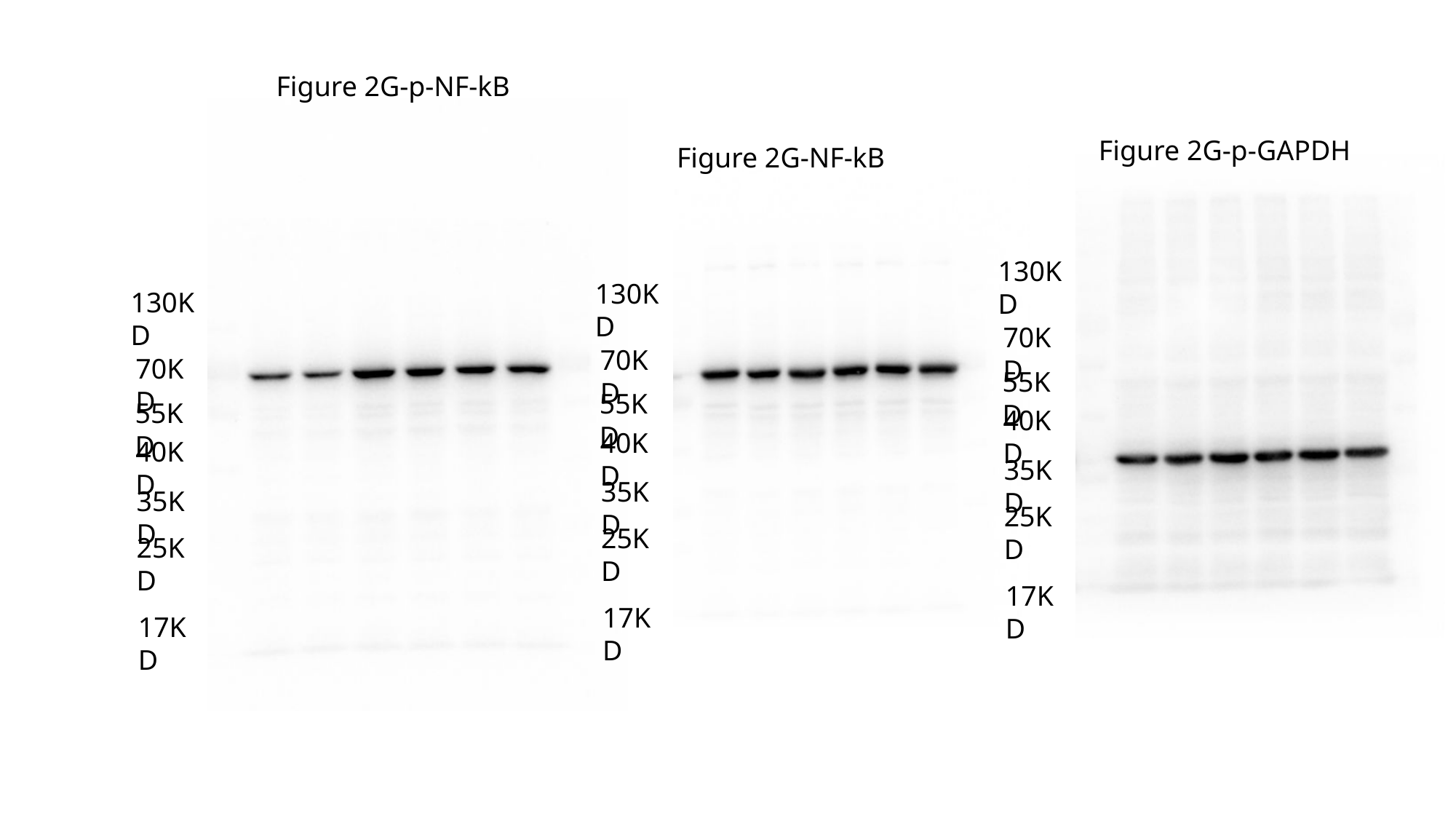

Figure 2G-p-NF-kB
Figure 2G-p-GAPDH
Figure 2G-NF-kB
130KD
130KD
130KD
70KD
70KD
70KD
55KD
55KD
55KD
40KD
40KD
40KD
35KD
35KD
35KD
25KD
25KD
25KD
17KD
17KD
17KD

## Slide 5
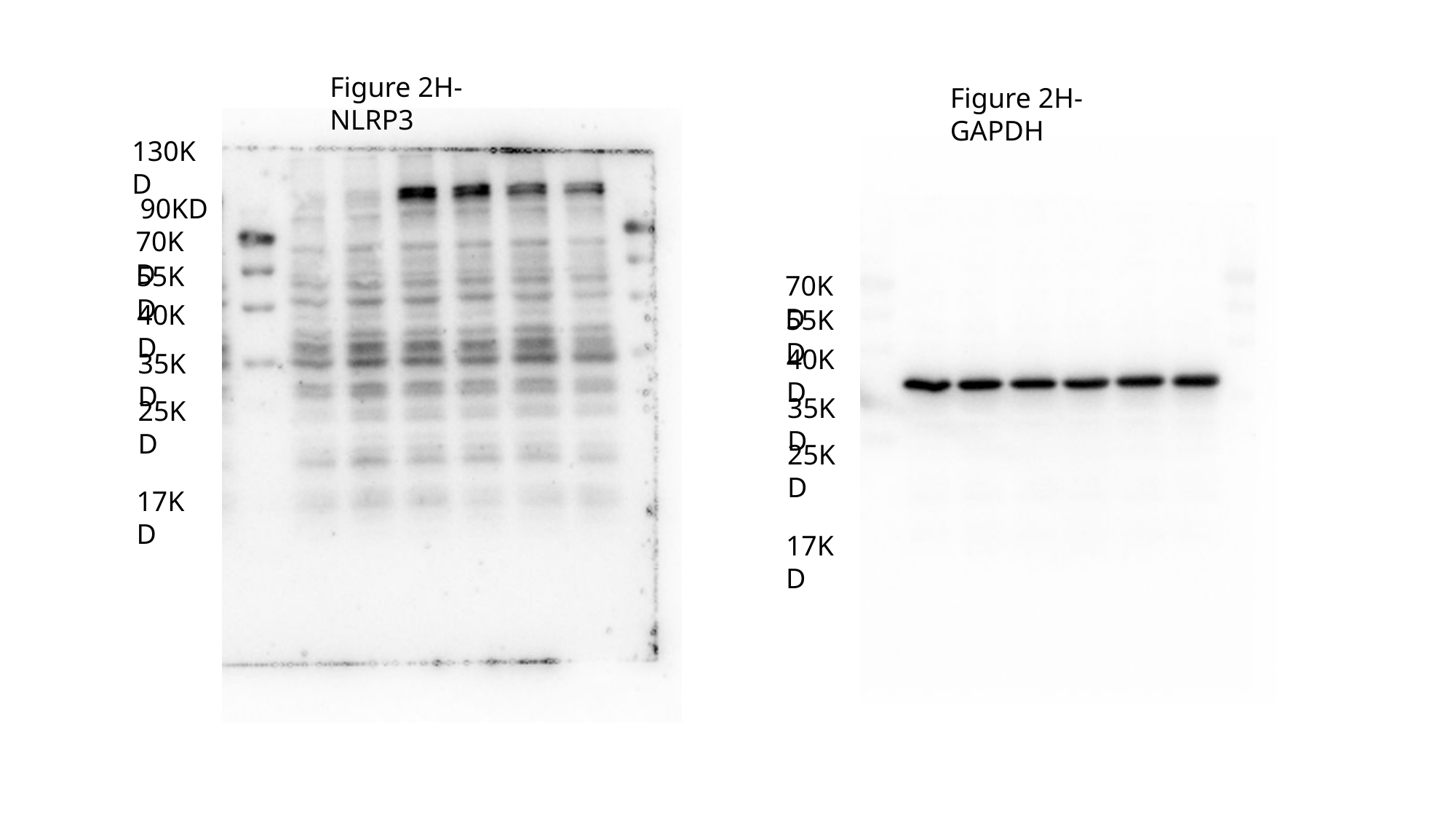

Figure 2H-NLRP3
Figure 2H-GAPDH
130KD
90KD
70KD
55KD
70KD
40KD
55KD
40KD
35KD
35KD
25KD
25KD
17KD
17KD

## Slide 6
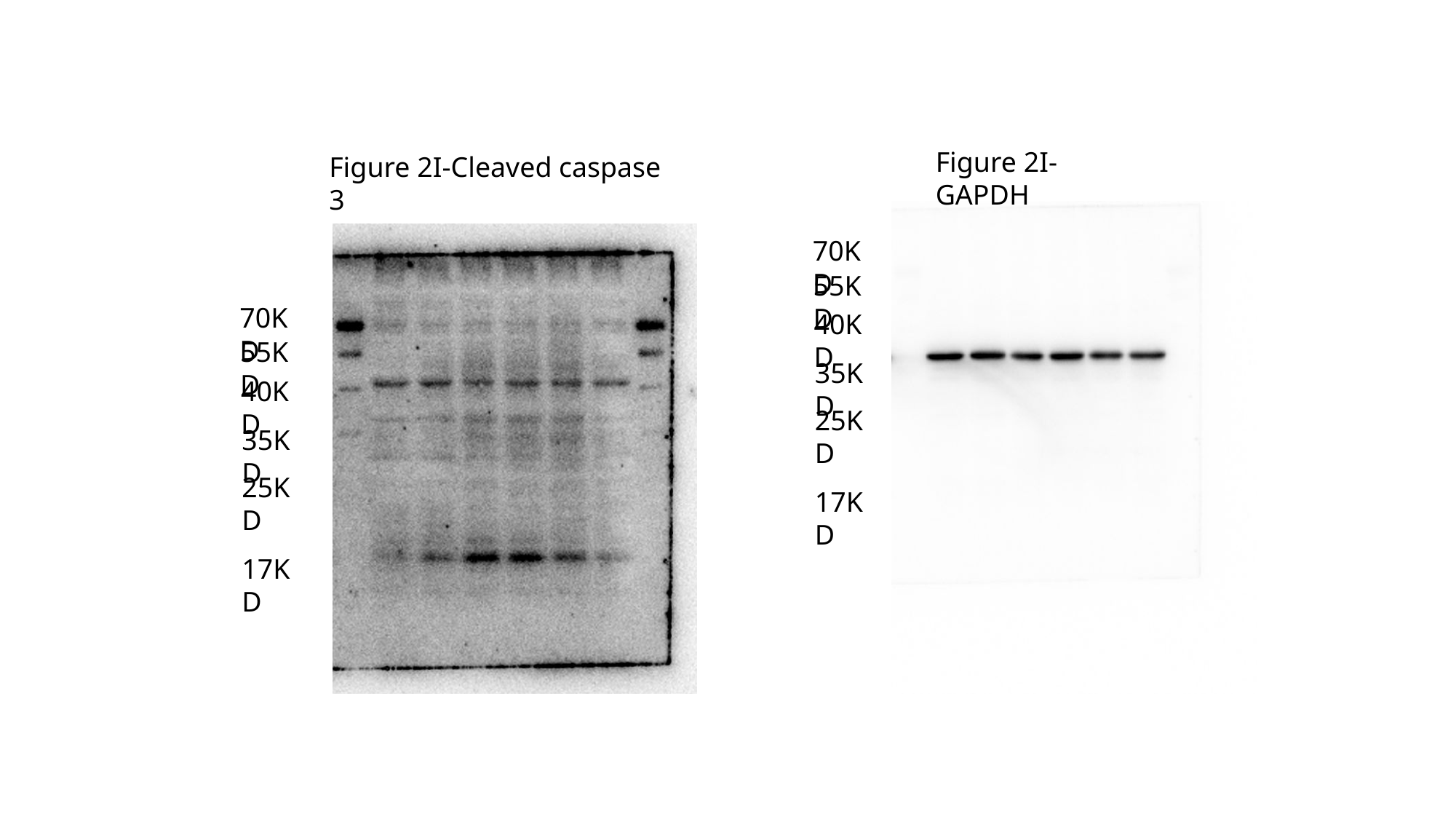

Figure 2I-GAPDH
Figure 2I-Cleaved caspase 3
70KD
55KD
70KD
40KD
55KD
35KD
40KD
25KD
35KD
25KD
17KD
17KD

## Slide 7
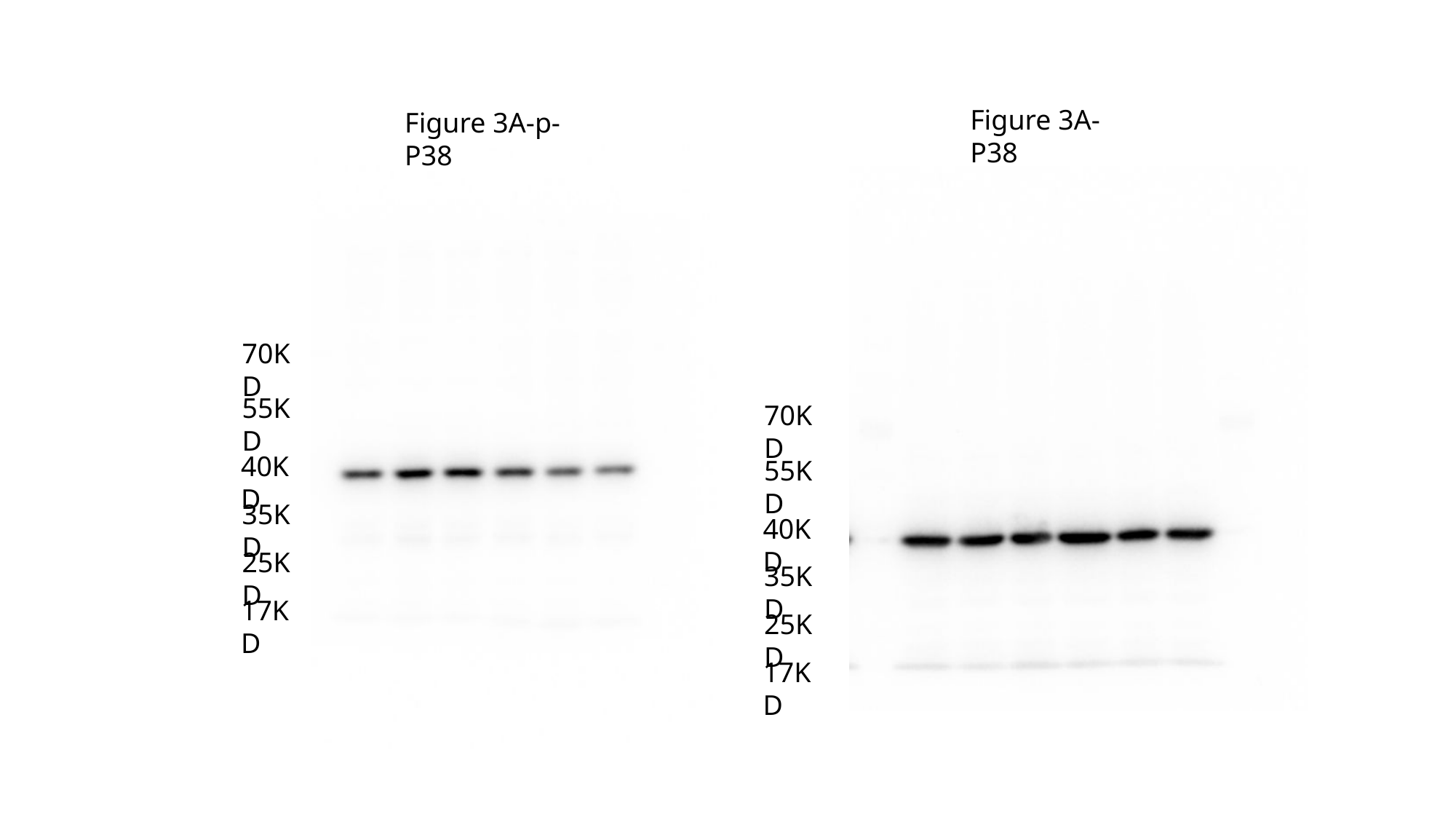

Figure 3A-P38
Figure 3A-p-P38
70KD
55KD
70KD
40KD
55KD
35KD
40KD
25KD
35KD
17KD
25KD
17KD

## Slide 8
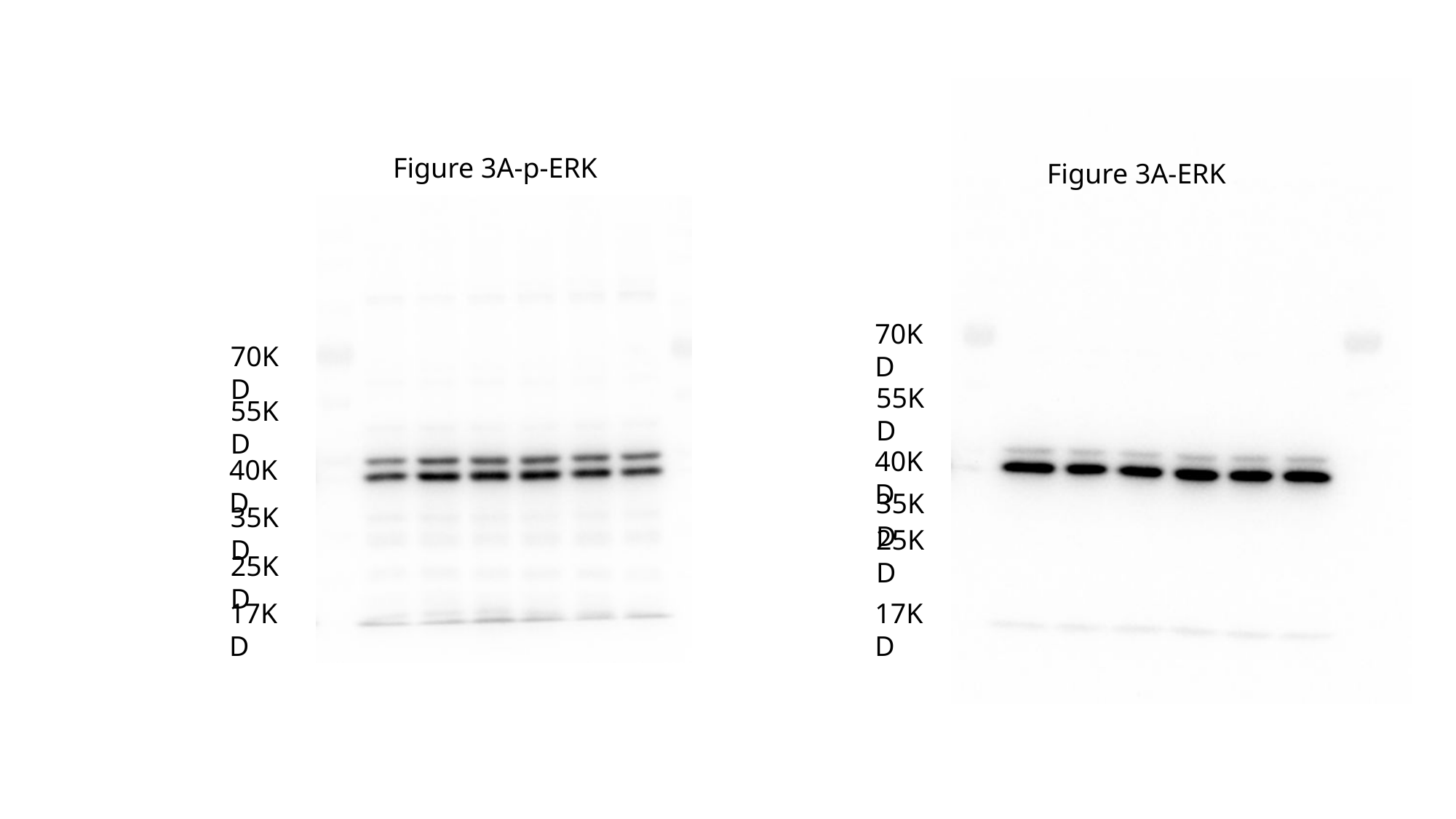

Figure 3A-p-ERK
Figure 3A-ERK
70KD
70KD
55KD
55KD
40KD
40KD
35KD
35KD
25KD
25KD
17KD
17KD

## Slide 9
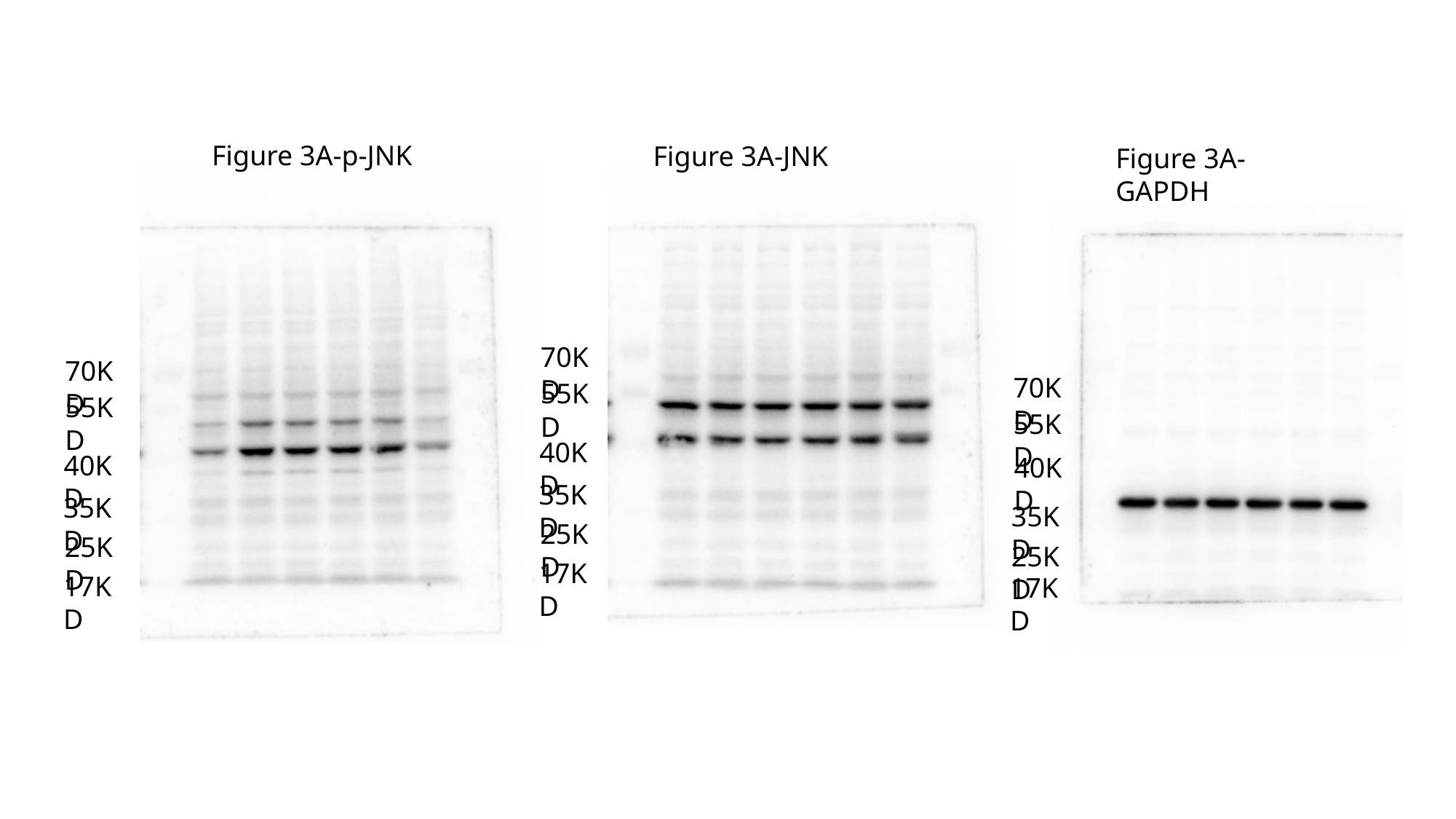

Figure 3A-p-JNK
Figure 3A-JNK
Figure 3A-GAPDH
70KD
70KD
70KD
55KD
55KD
55KD
40KD
40KD
40KD
35KD
35KD
35KD
25KD
25KD
25KD
17KD
17KD
17KD

## Slide 10
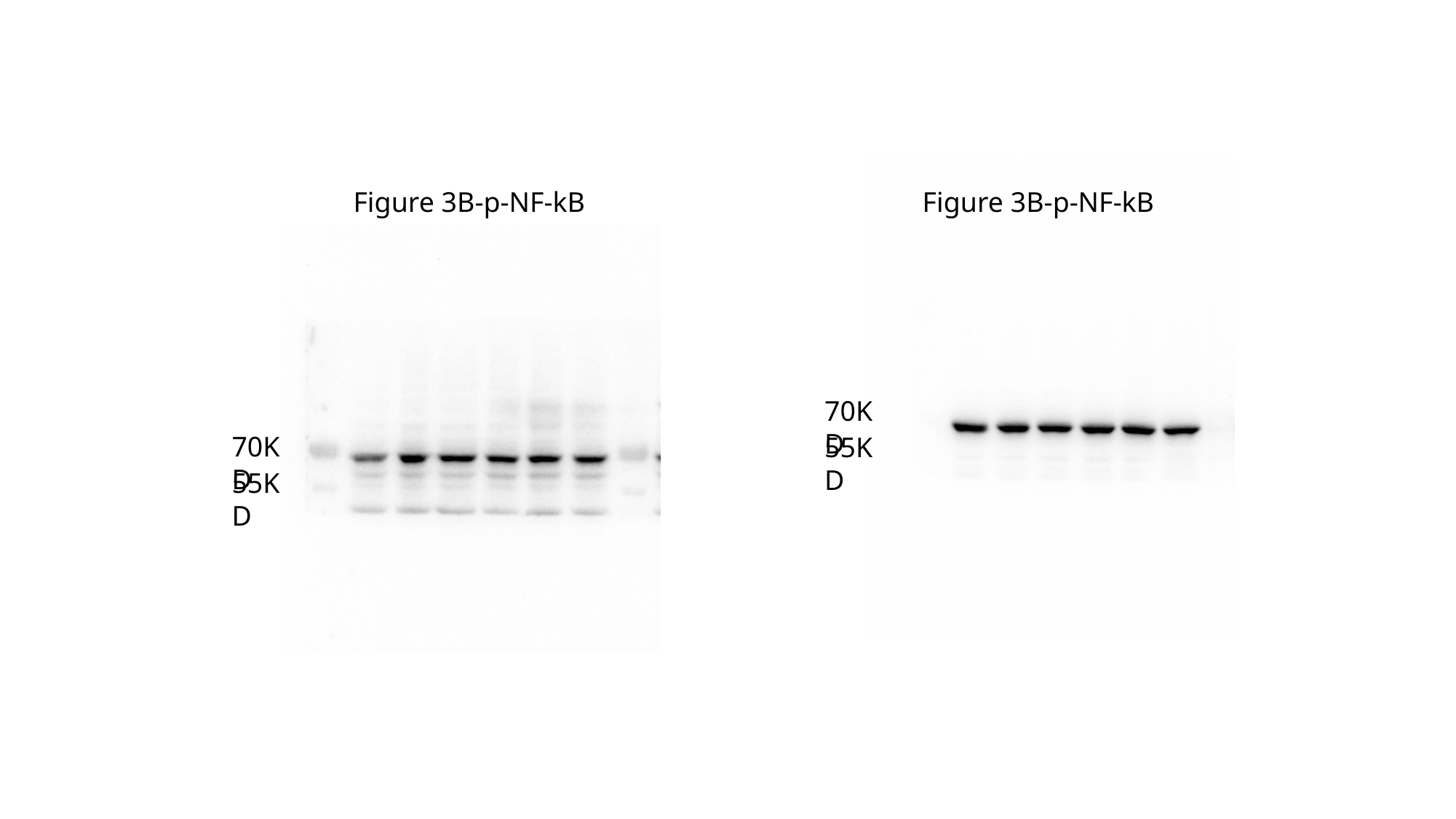

Figure 3B-p-NF-kB
Figure 3B-p-NF-kB
70KD
70KD
55KD
55KD

## Slide 11
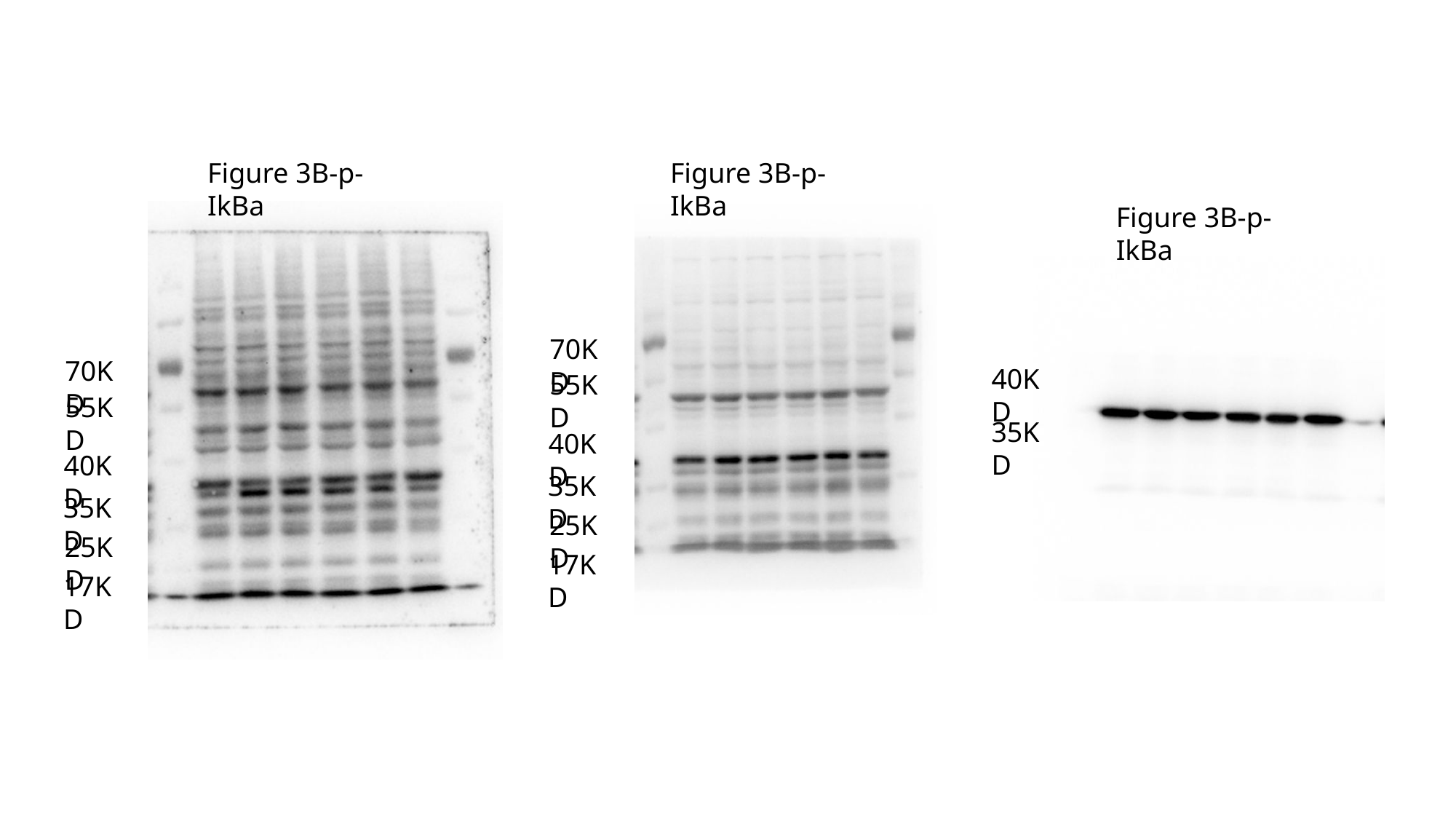

Figure 3B-p-IkBa
Figure 3B-p-IkBa
Figure 3B-p-IkBa
70KD
70KD
40KD
55KD
55KD
35KD
40KD
40KD
35KD
35KD
25KD
25KD
17KD
17KD

## Slide 12
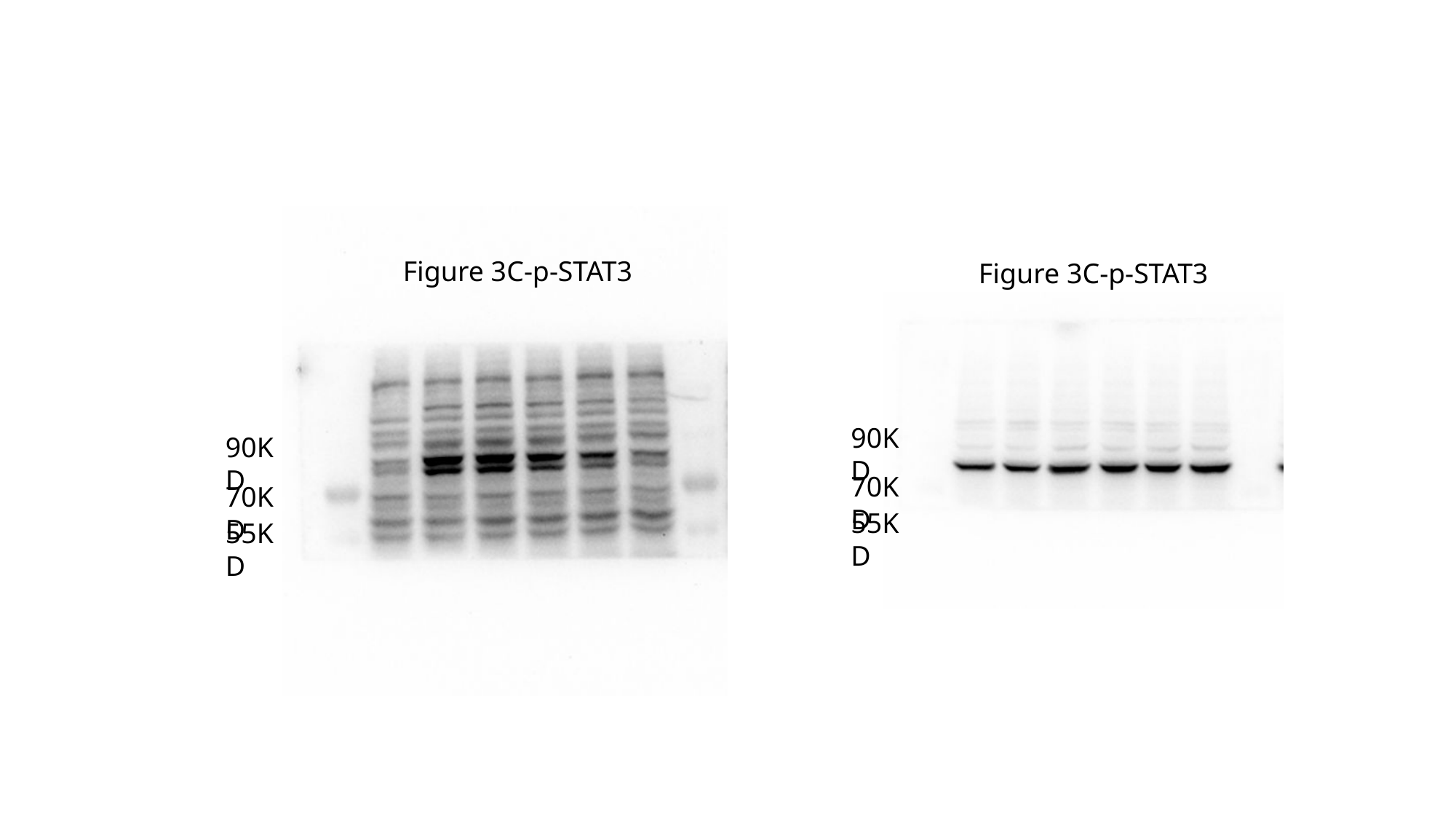

Figure 3C-p-STAT3
Figure 3C-p-STAT3
90KD
90KD
70KD
70KD
55KD
55KD

## Slide 13
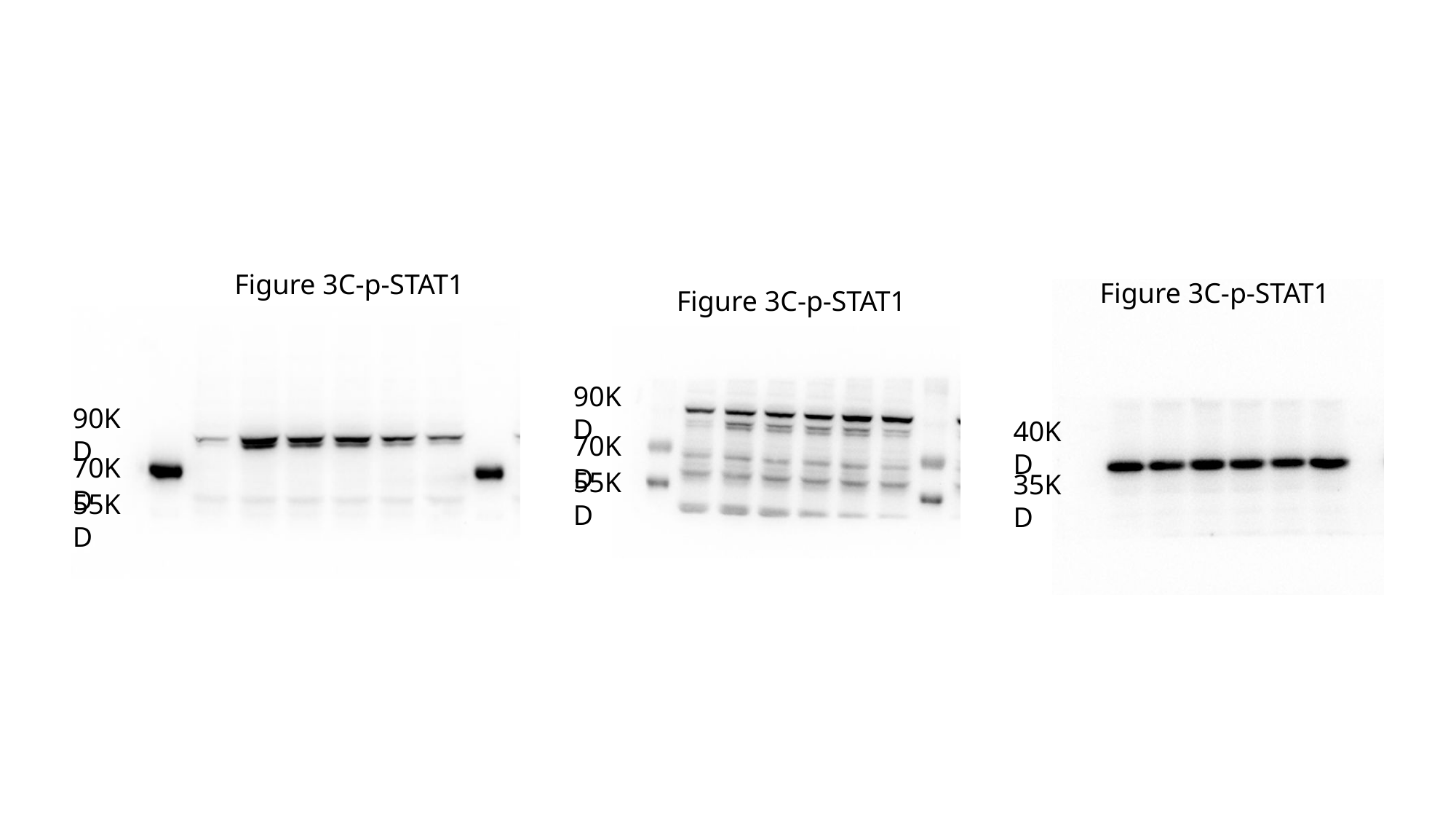

Figure 3C-p-STAT1
Figure 3C-p-STAT1
Figure 3C-p-STAT1
90KD
90KD
40KD
70KD
70KD
55KD
35KD
55KD

## Slide 14
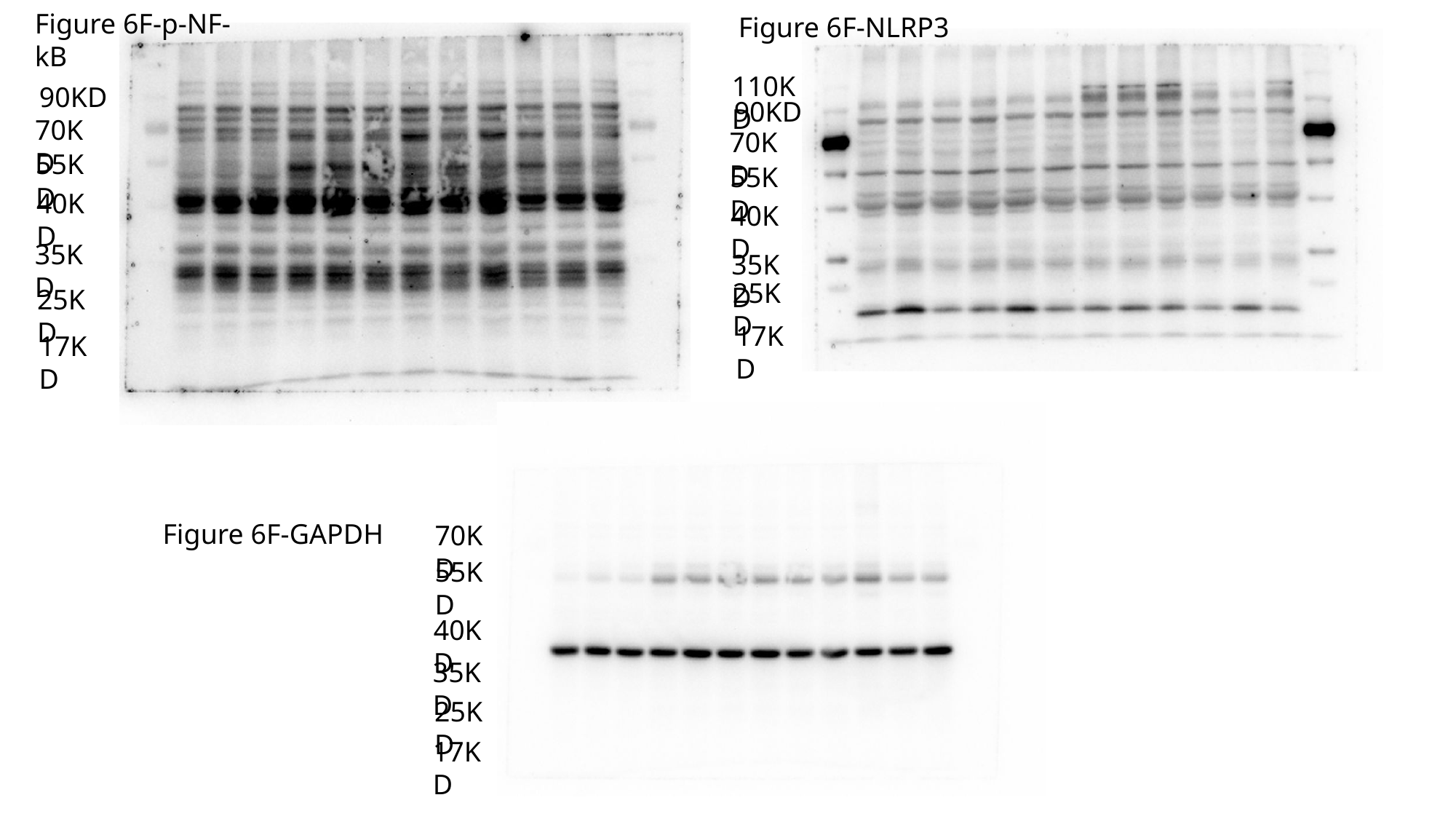

Figure 6F-p-NF-kB
Figure 6F-NLRP3
110KD
90KD
90KD
70KD
70KD
55KD
55KD
40KD
40KD
35KD
35KD
25KD
25KD
17KD
17KD
Figure 6F-GAPDH
70KD
55KD
40KD
35KD
25KD
17KD

## Slide 15
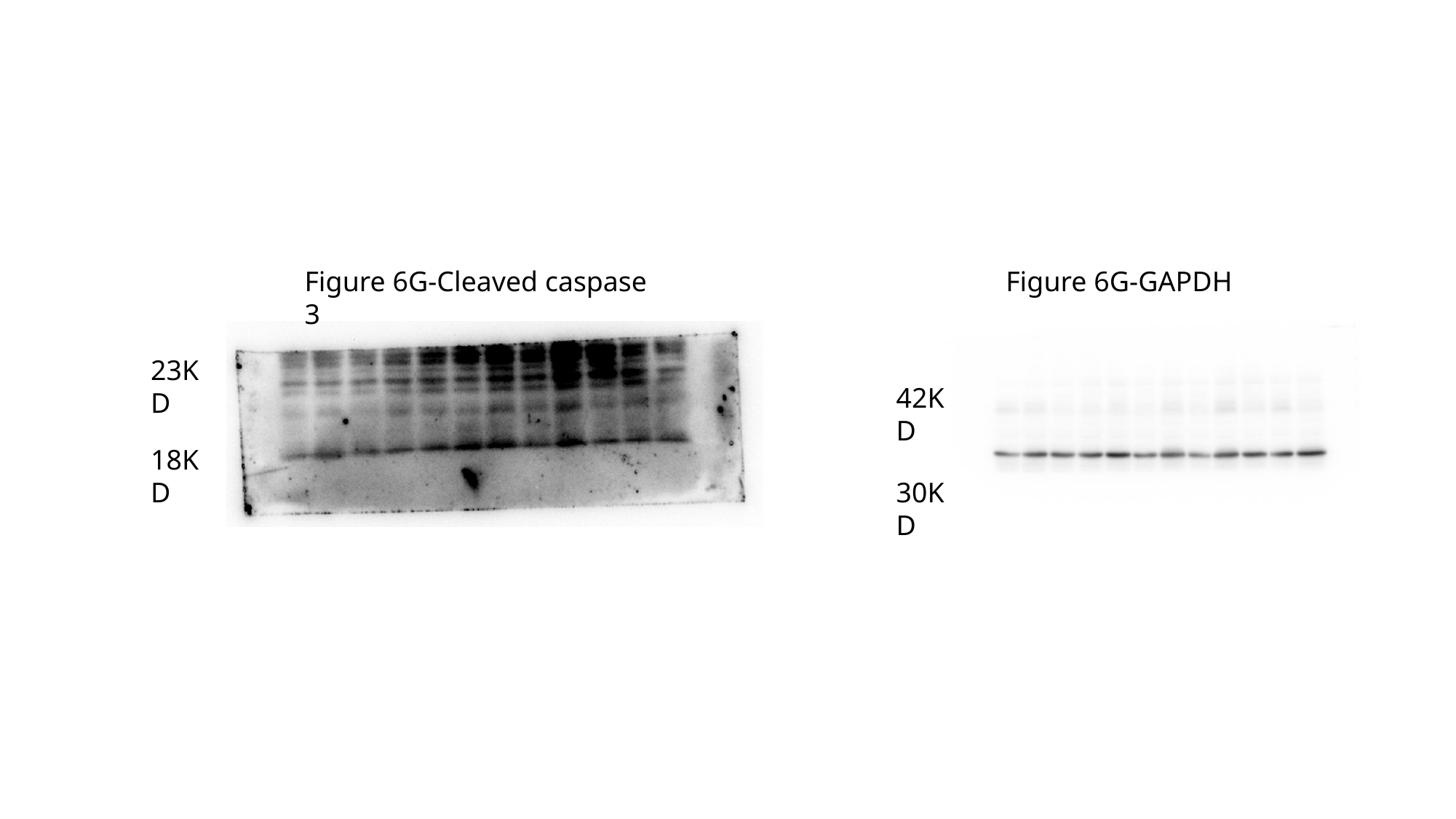

Figure 6G-Cleaved caspase 3
Figure 6G-GAPDH
23KD
42KD
18KD
30KD
