# Supplementary material for: Harmine Alleviated Sepsis-Induced Cardiac Dysfunction by Modulating Macrophage Polarization via the STAT/MAPK/NF-κB Pathway
Source: Front Cell Dev Biol. 2022 Jan 17;9:792257. doi: 10.3389/fcell.2021.792257 (PMC8801946; doi:10.3389/fcell.2021.792257)
Supplement: Supplementary file 3 [file Presentation6.PPTX]

## Slide 1
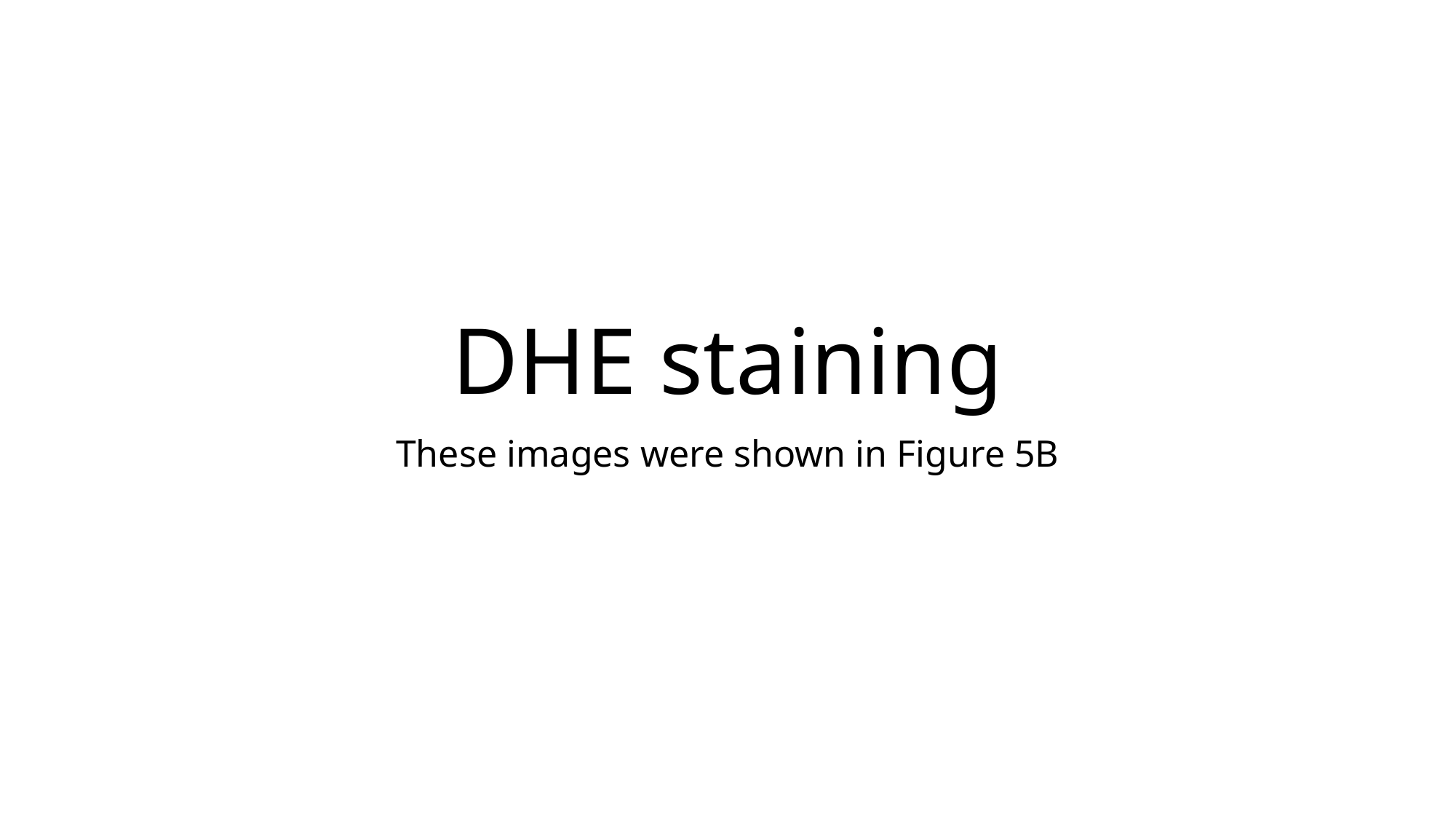

# DHE staining
These images were shown in Figure 5B

## Slide 2
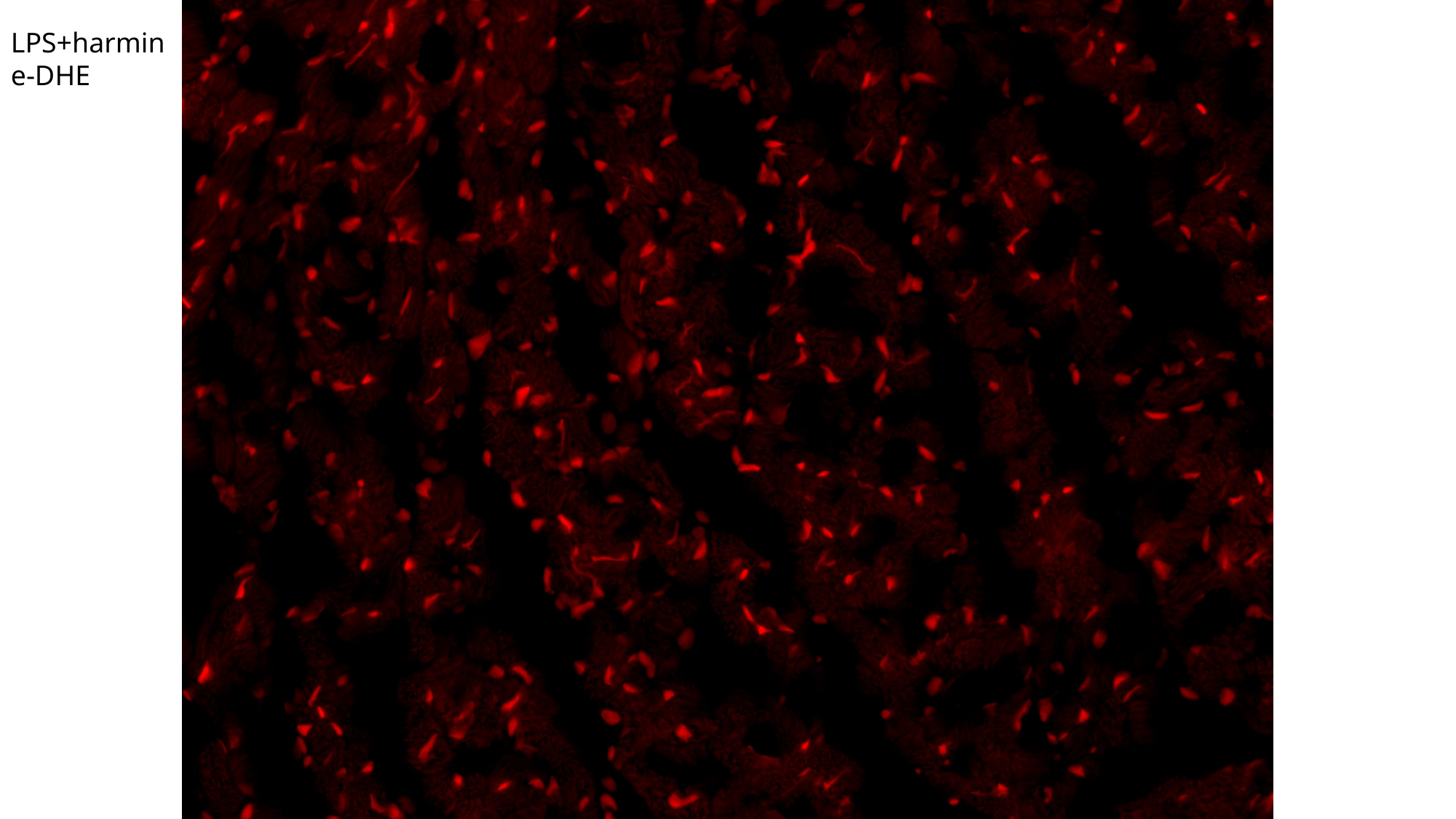

LPS+harmine-DHE

## Slide 3
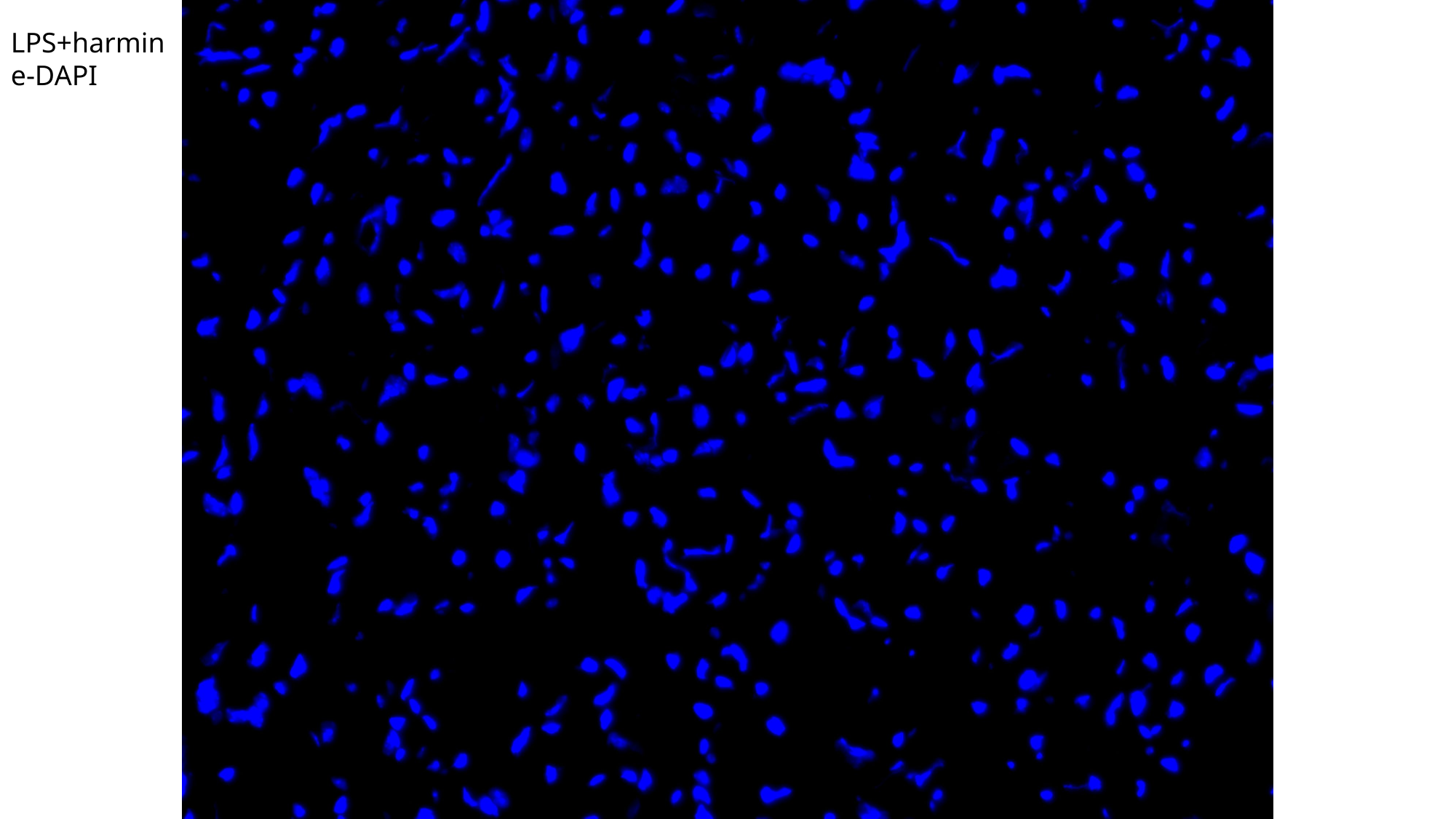

LPS+harmine-DAPI

## Slide 4
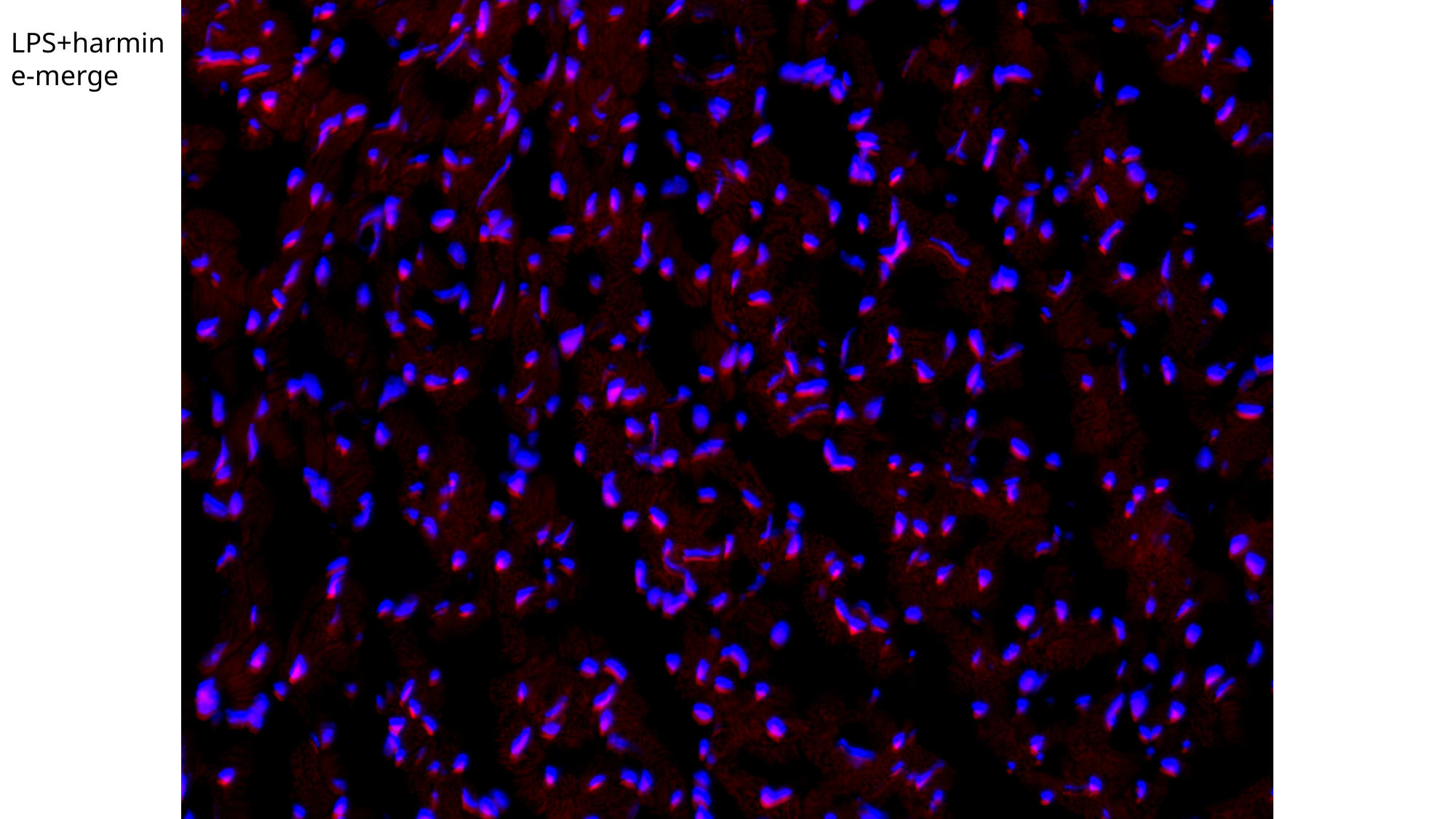

LPS+harmine-merge
