# Supplementary material for: Harmine Alleviated Sepsis-Induced Cardiac Dysfunction by Modulating Macrophage Polarization via the STAT/MAPK/NF-κB Pathway
Source: Front Cell Dev Biol. 2022 Jan 17;9:792257. doi: 10.3389/fcell.2021.792257 (PMC8801946; doi:10.3389/fcell.2021.792257)
Supplement: Supplementary file 5 [file Presentation2.PPTX]

## Slide 1
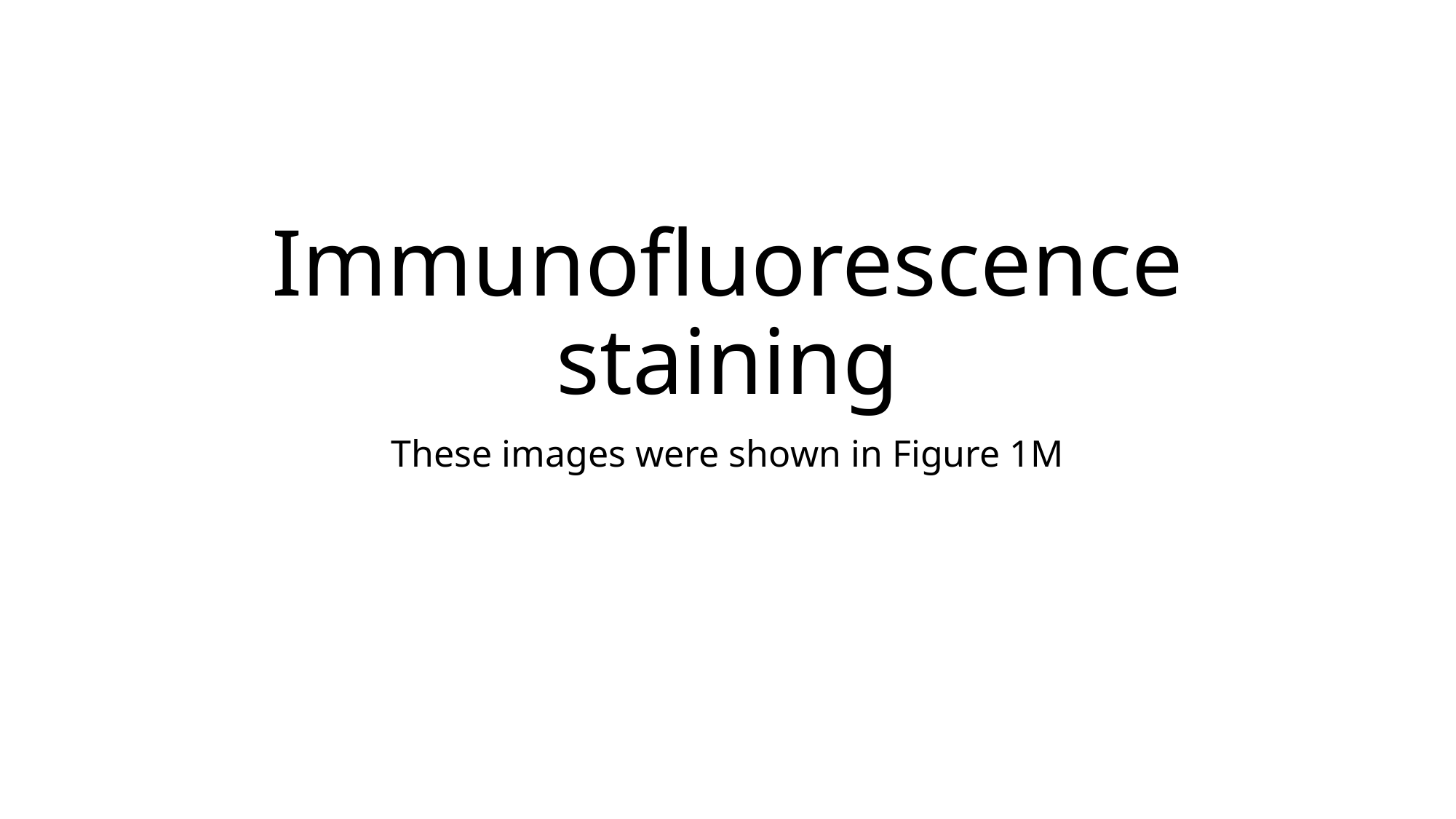

# Immunofluorescence staining
These images were shown in Figure 1M

## Slide 2
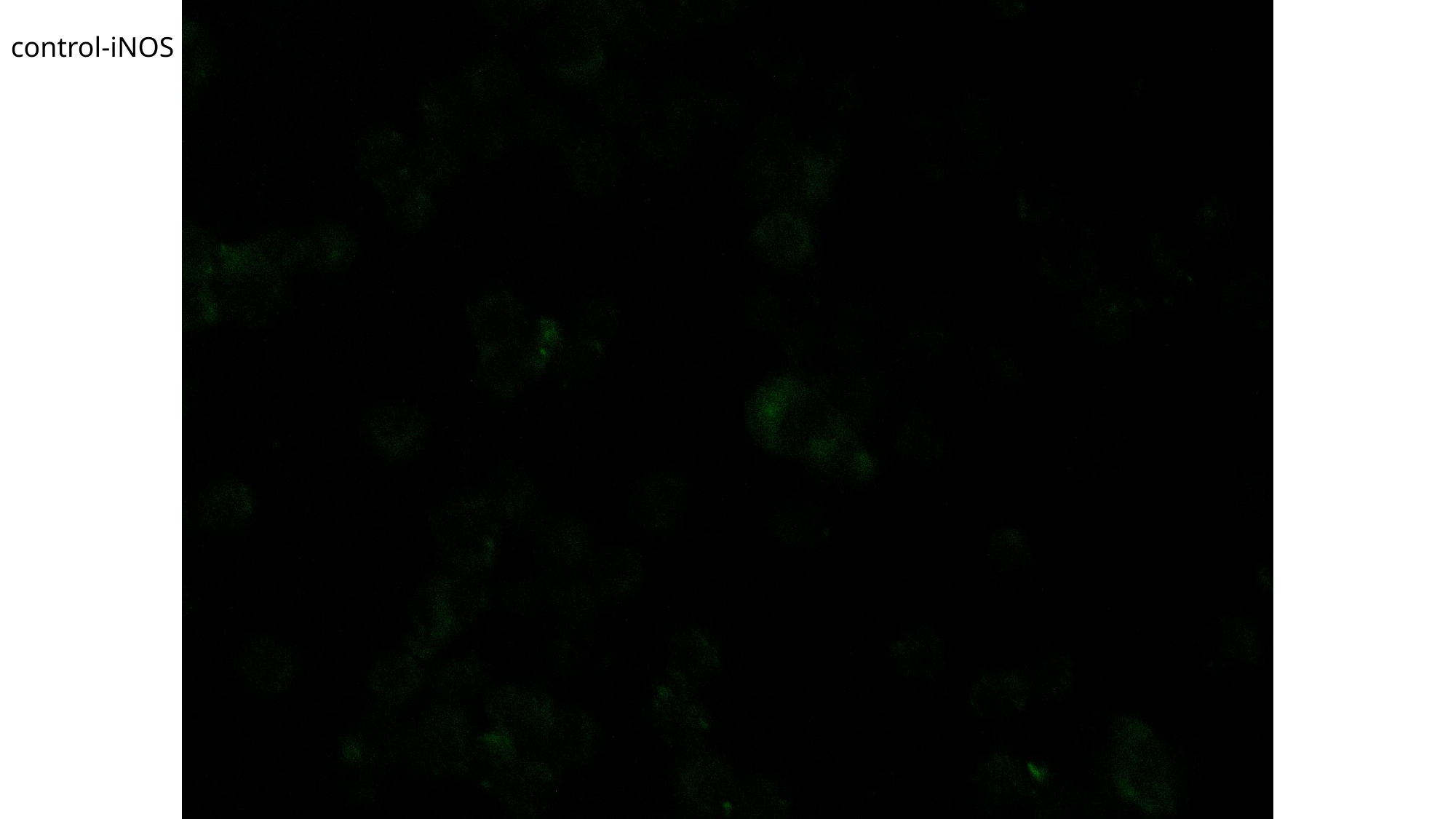

control-iNOS

## Slide 3
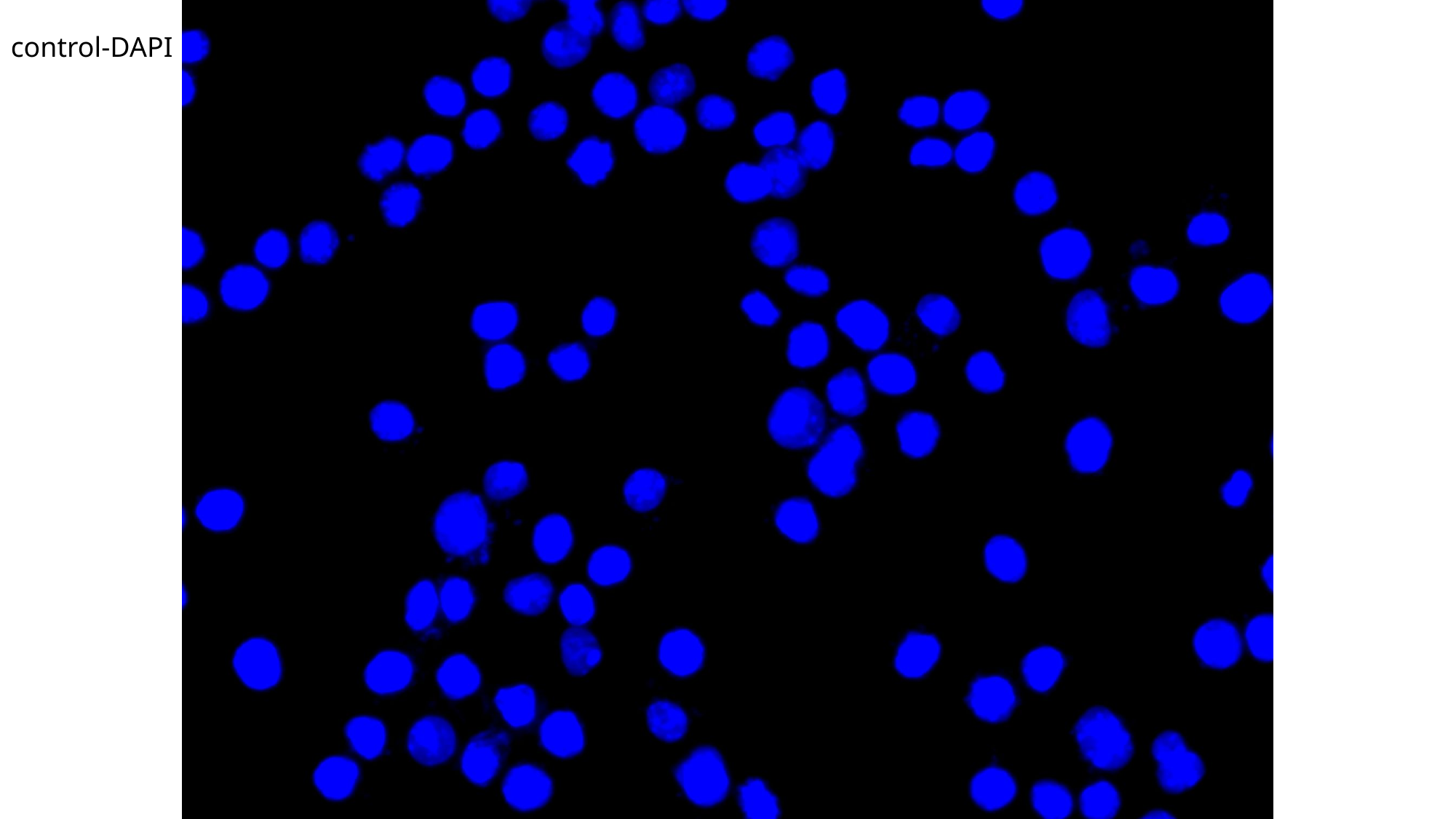

control-DAPI

## Slide 4
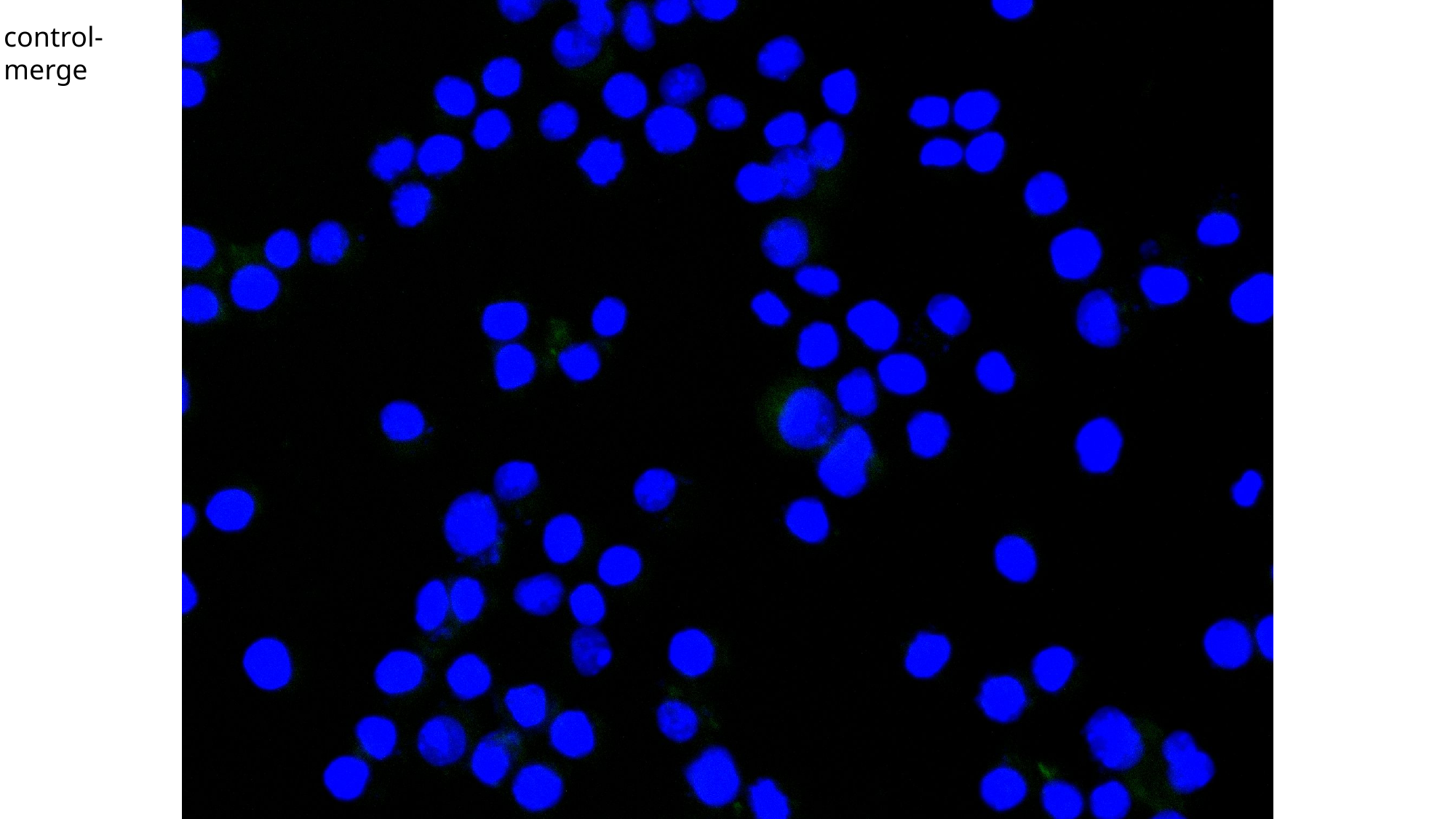

control-merge

## Slide 5
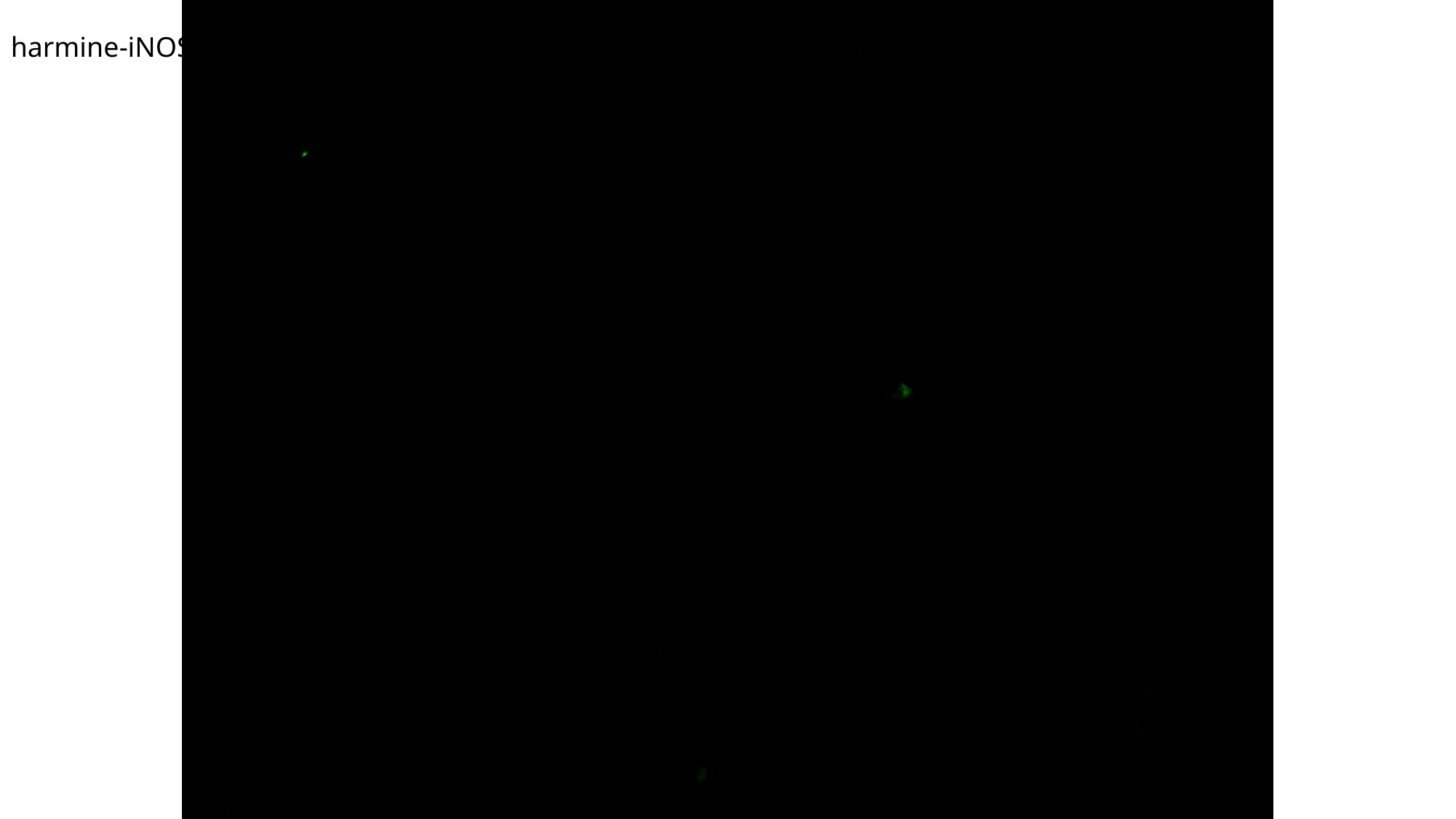

harmine-iNOS

## Slide 6
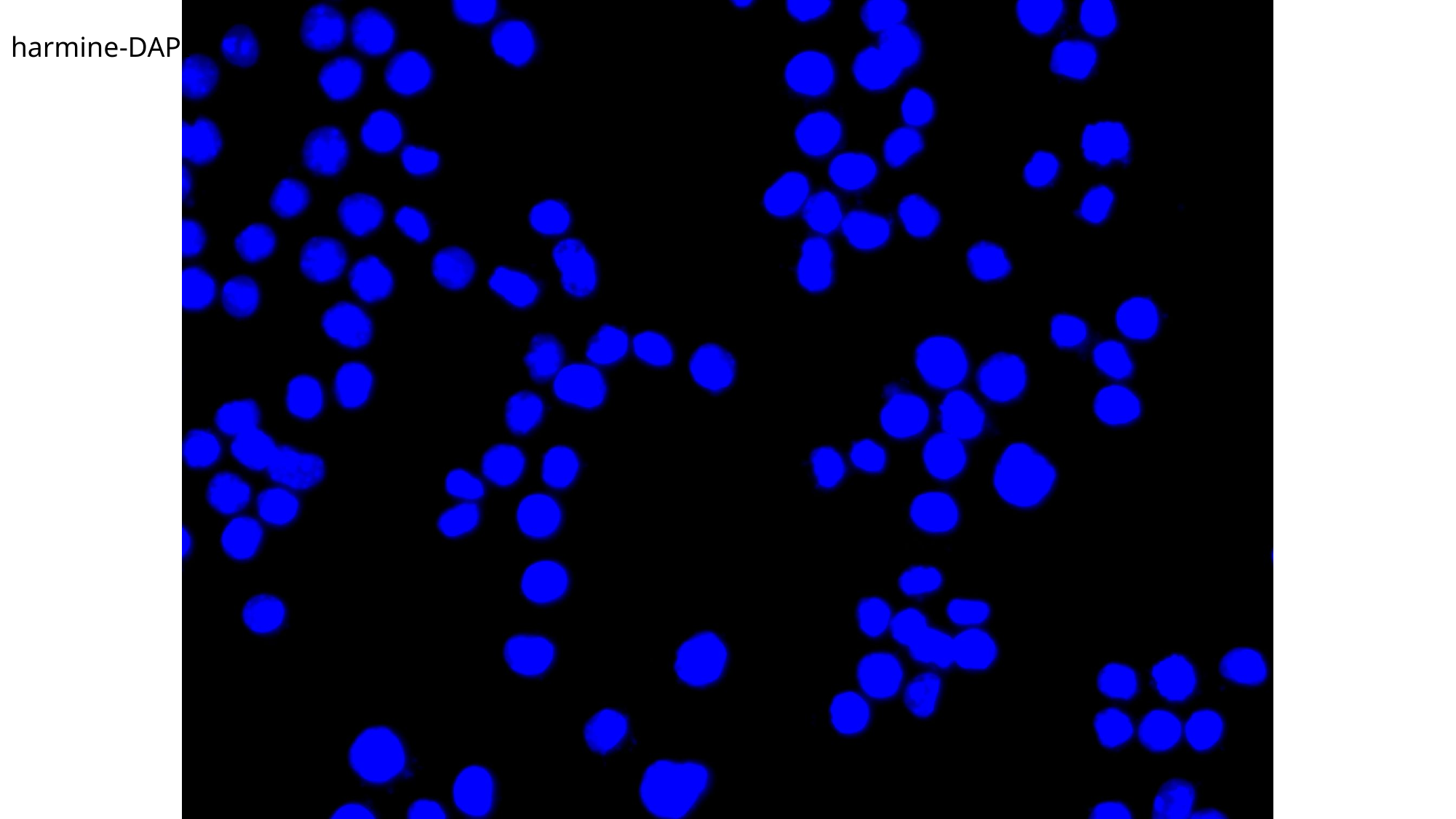

harmine-DAPI

## Slide 7
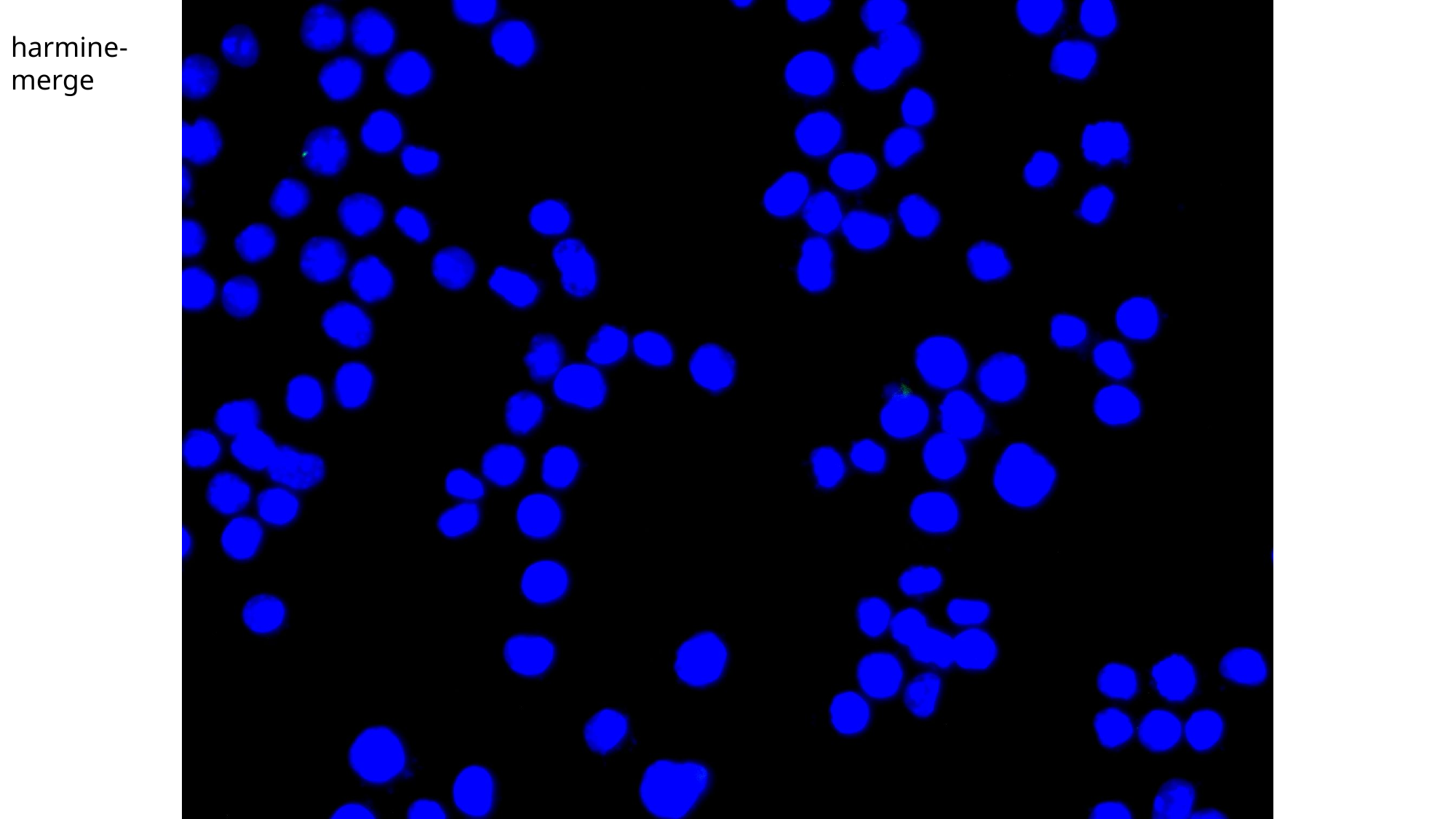

harmine-merge

## Slide 8
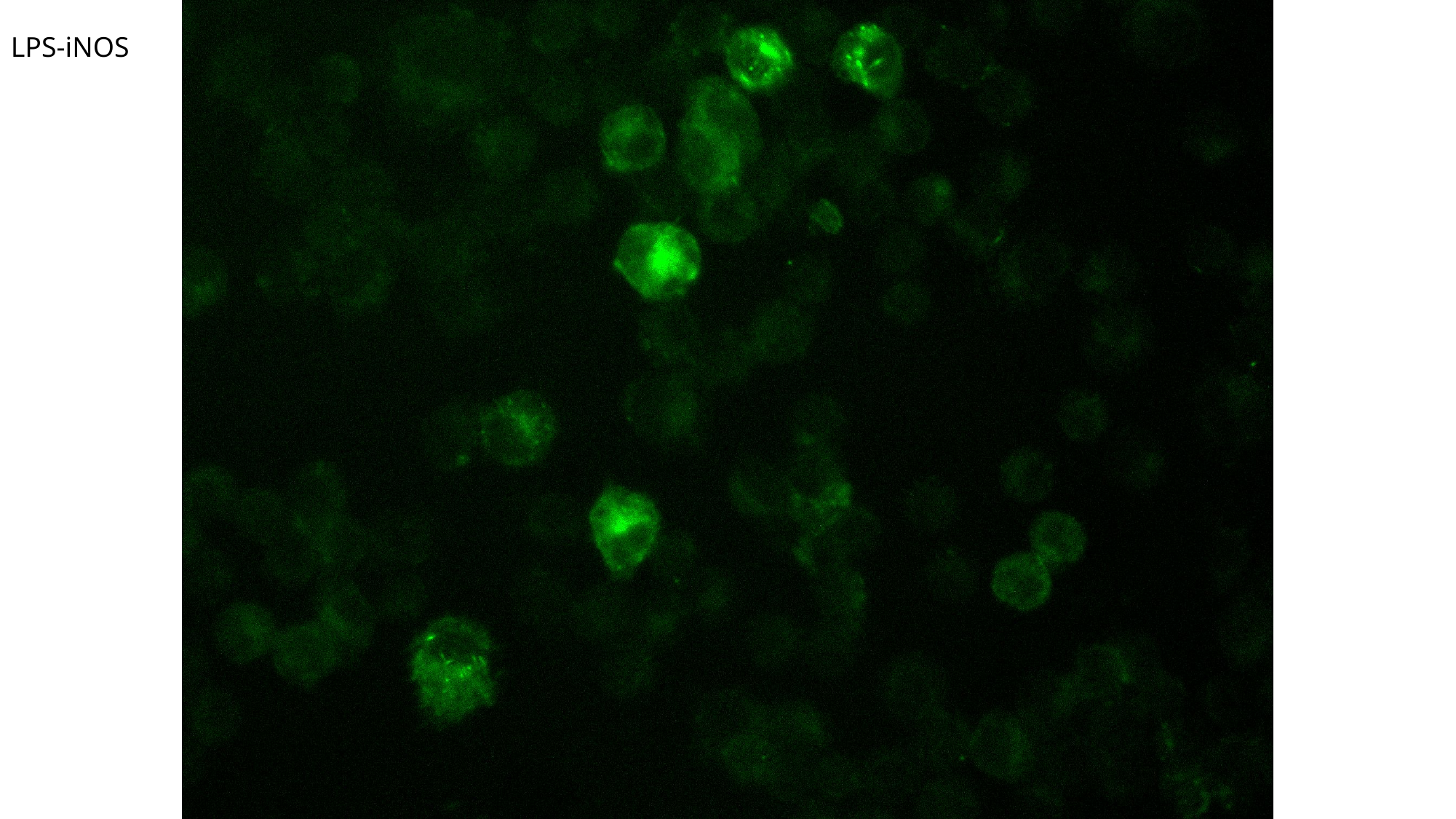

LPS-iNOS

## Slide 9
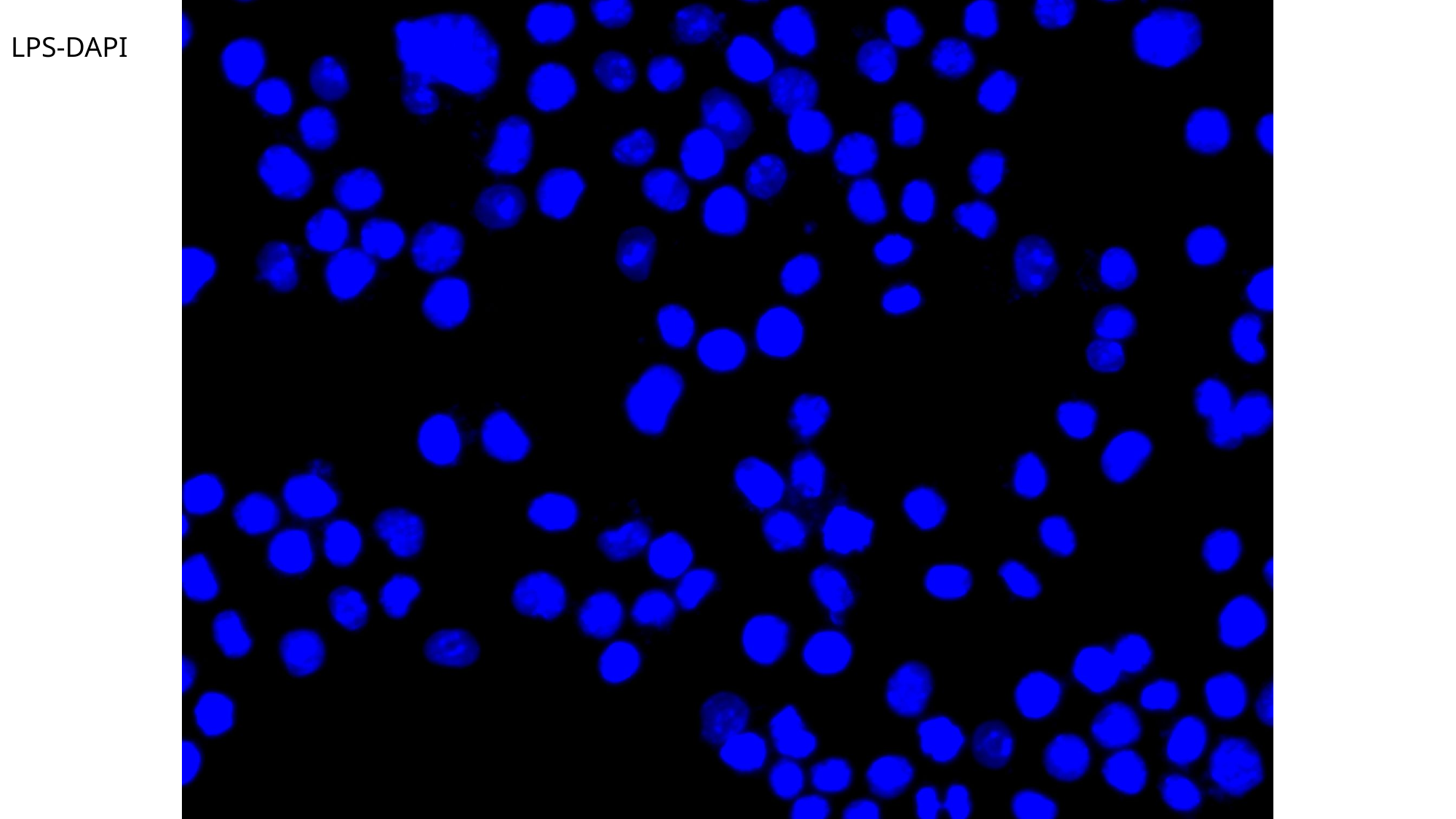

LPS-DAPI

## Slide 10
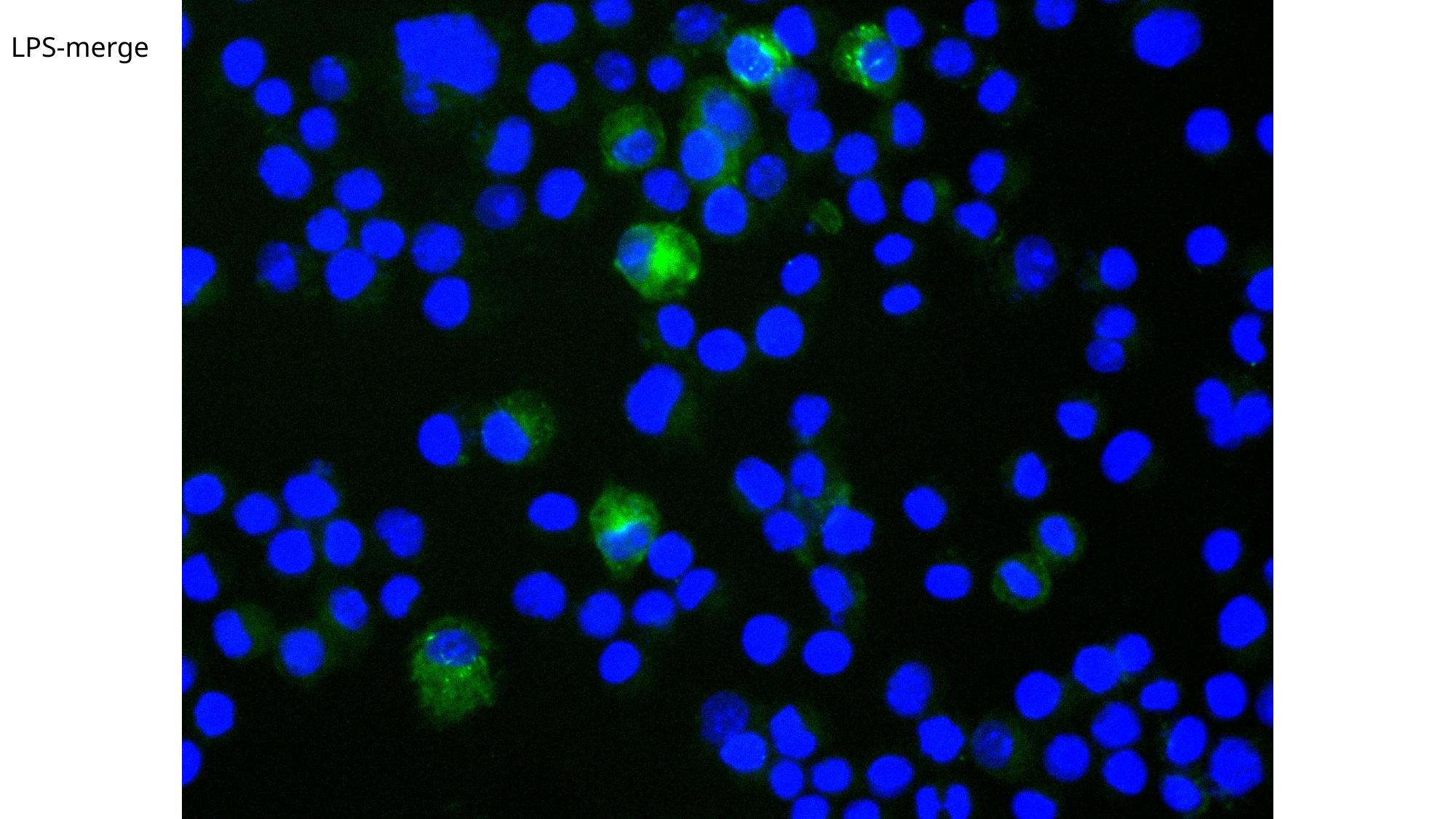

LPS-merge

## Slide 11
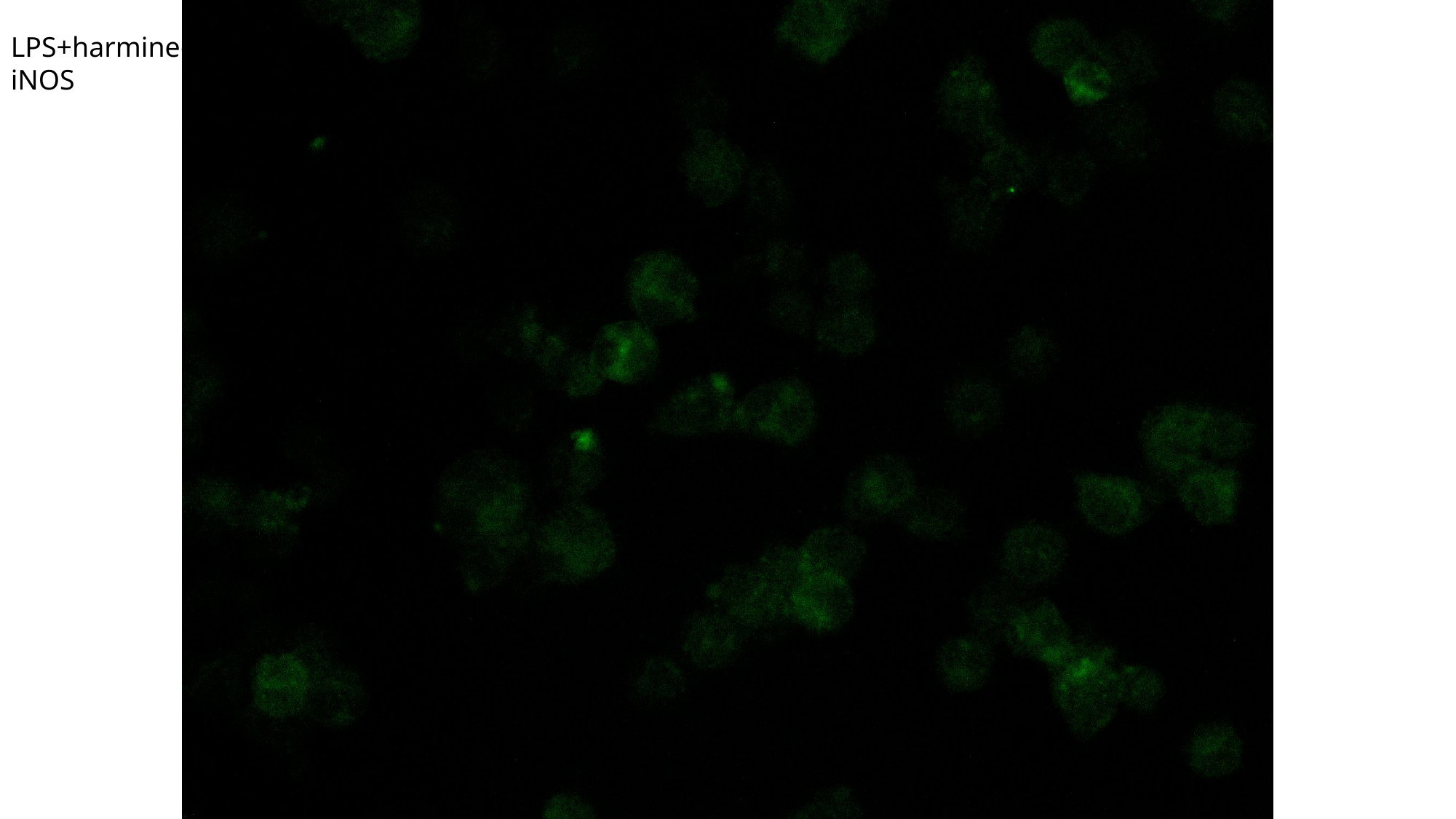

LPS+harmine-iNOS

## Slide 12
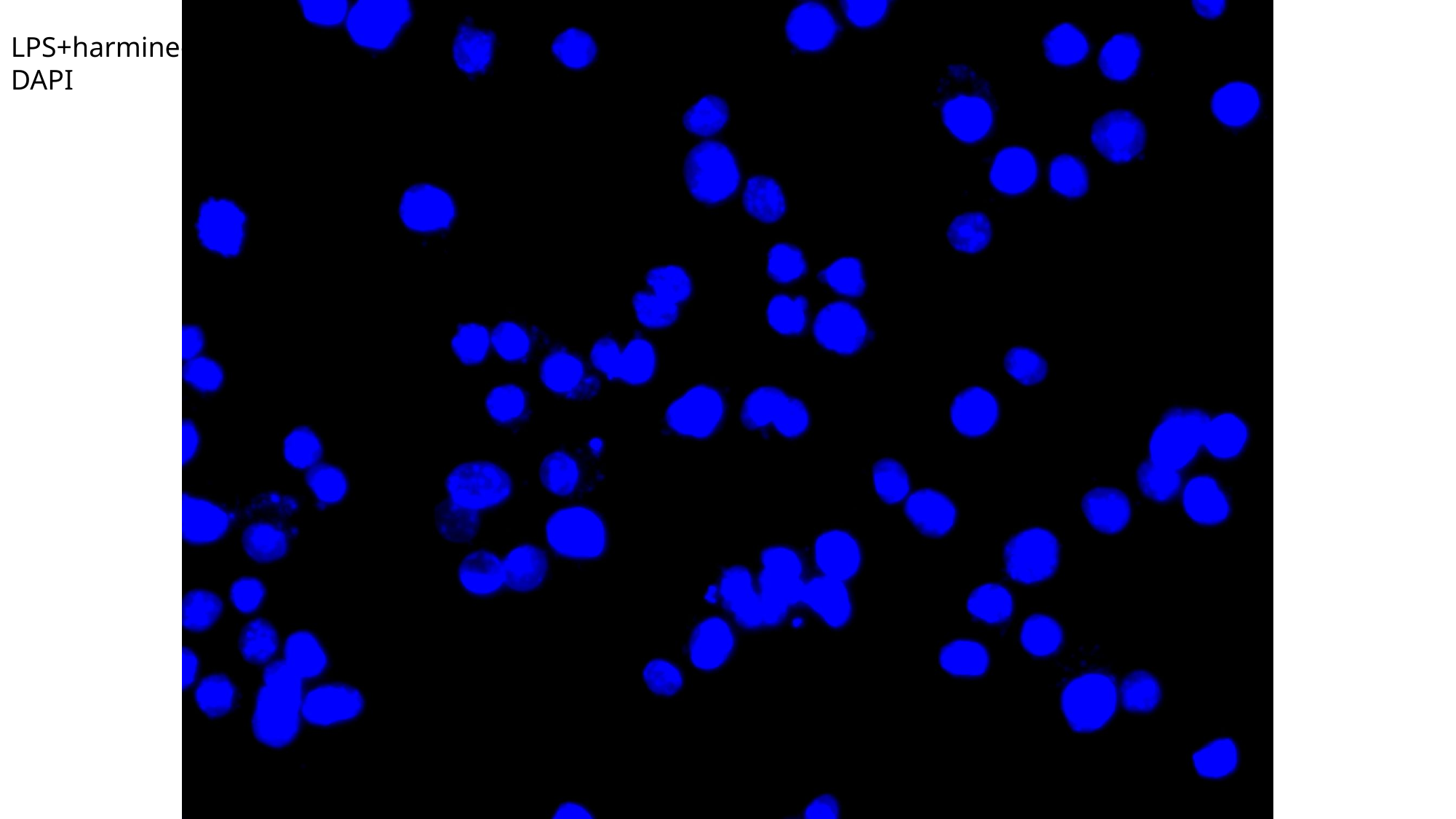

LPS+harmine-DAPI

## Slide 13
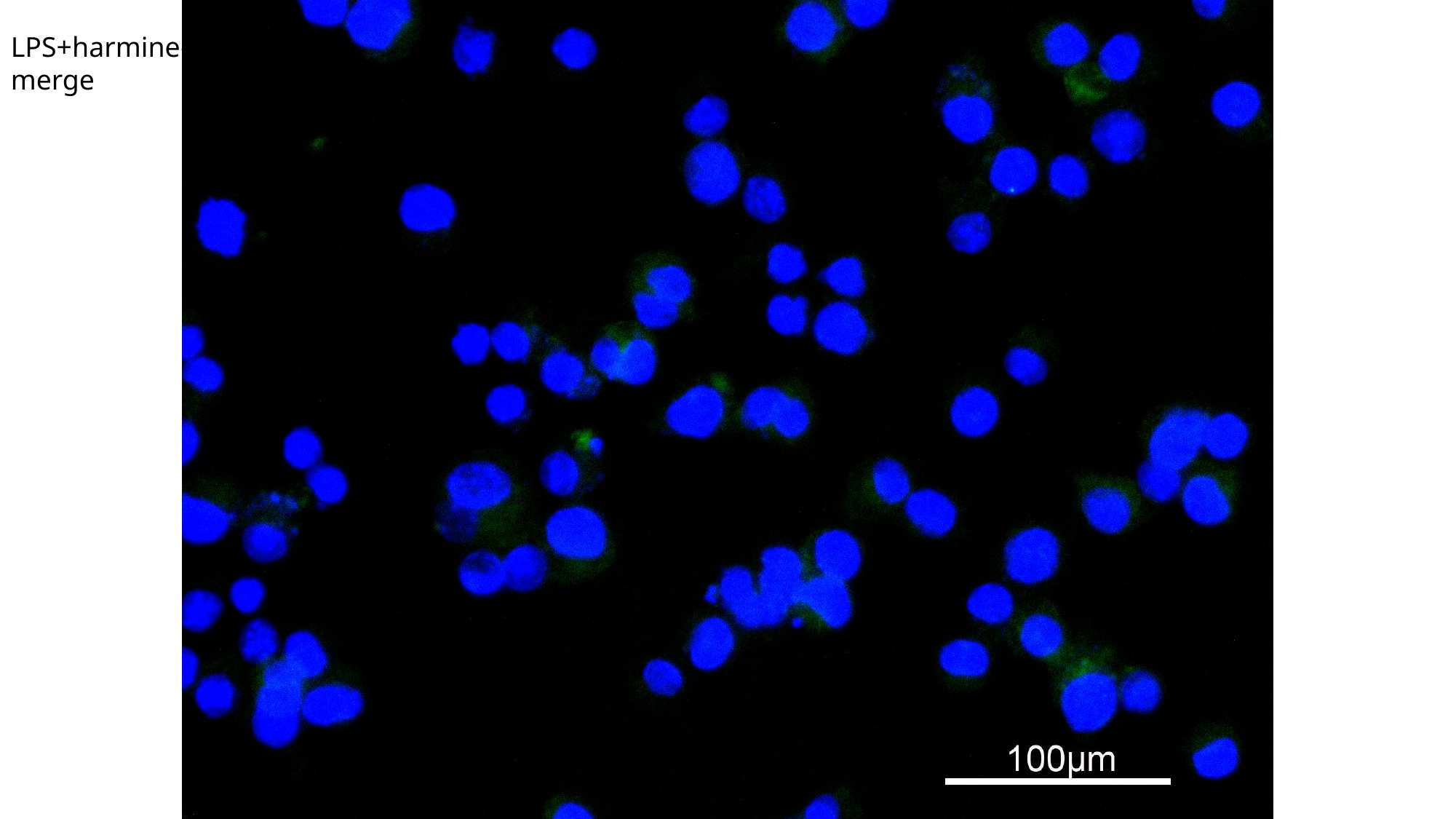

LPS+harmine-merge

## Slide 14
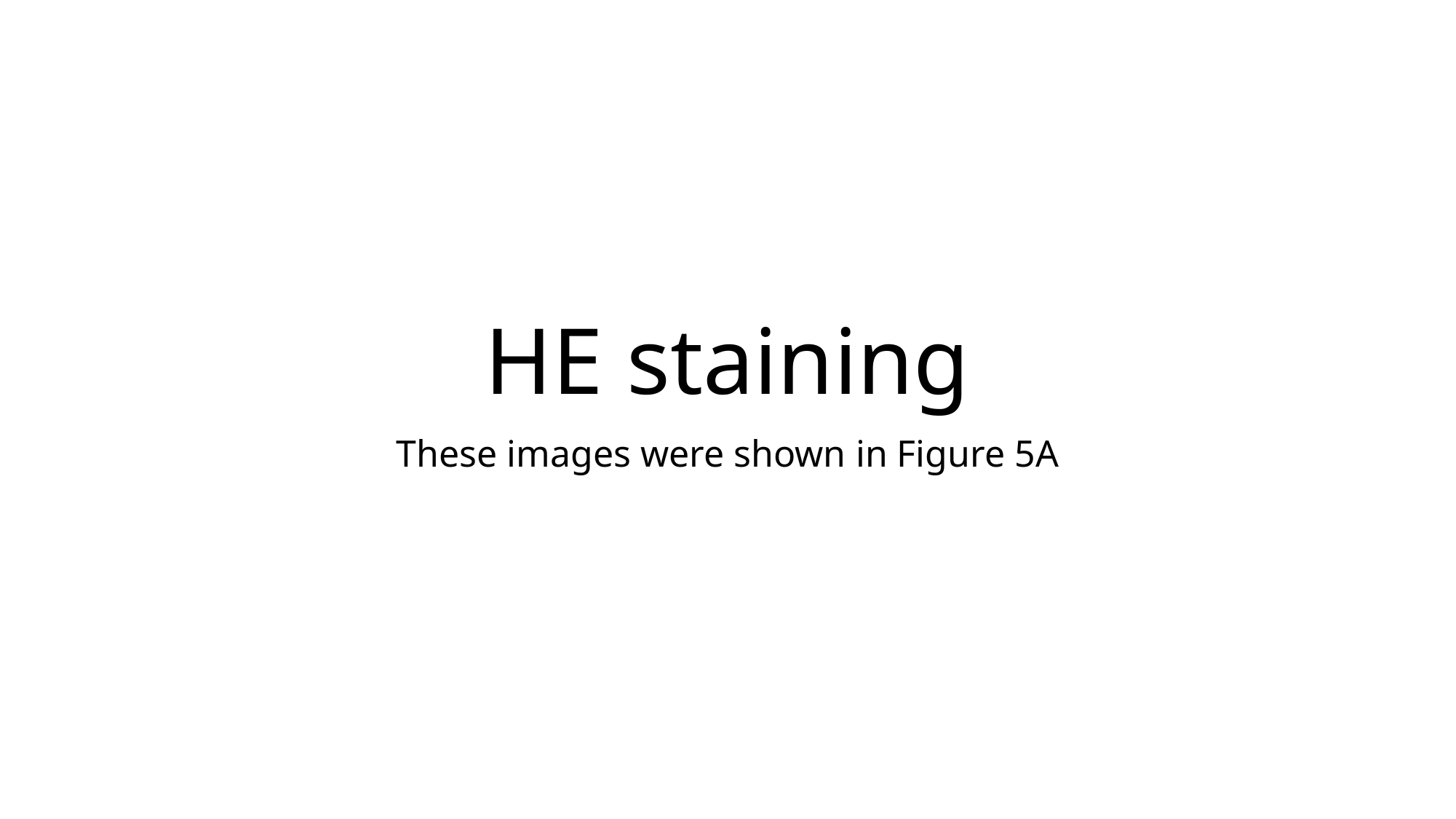

# HE staining
These images were shown in Figure 5A

## Slide 15
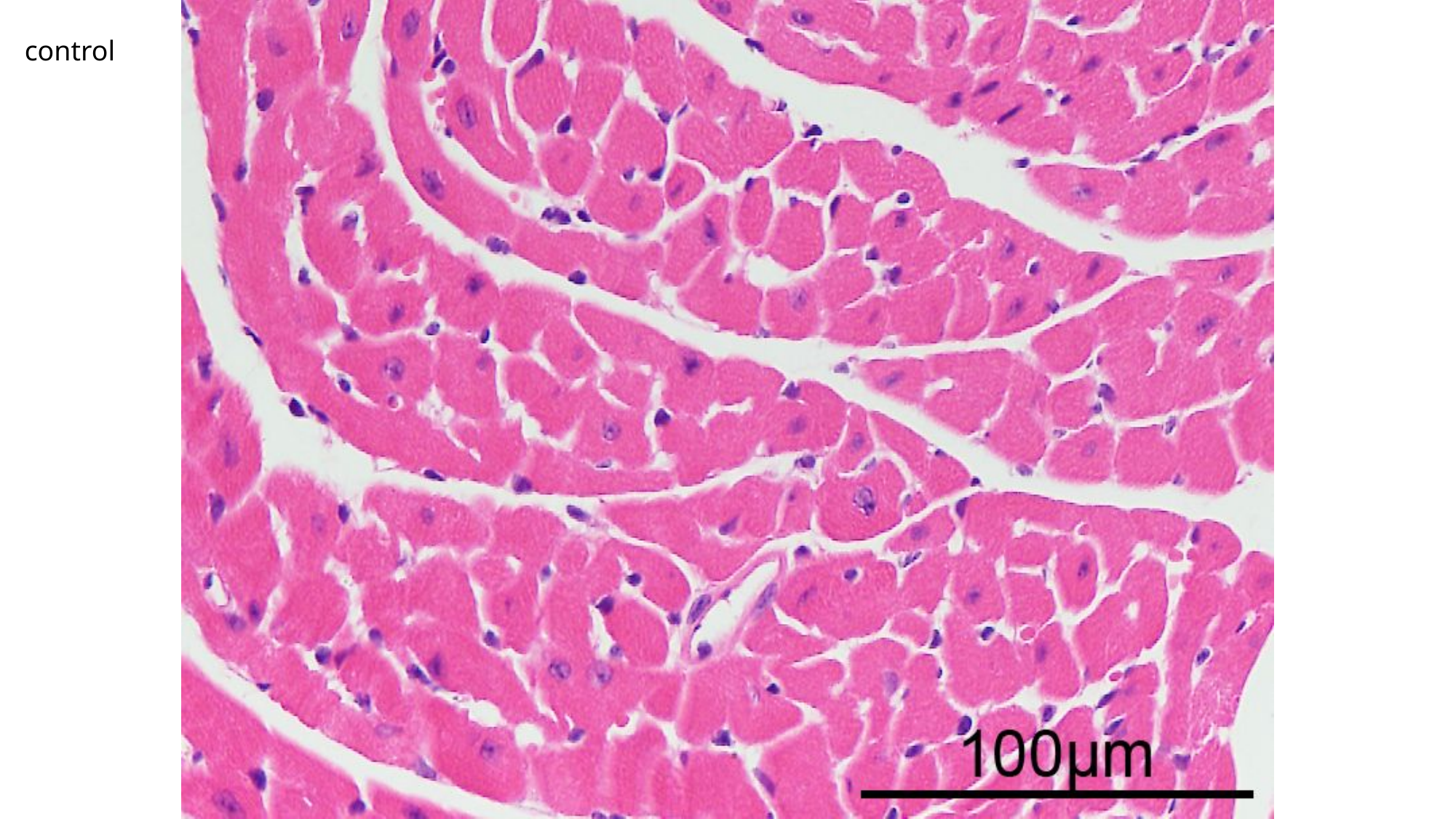

control

## Slide 16
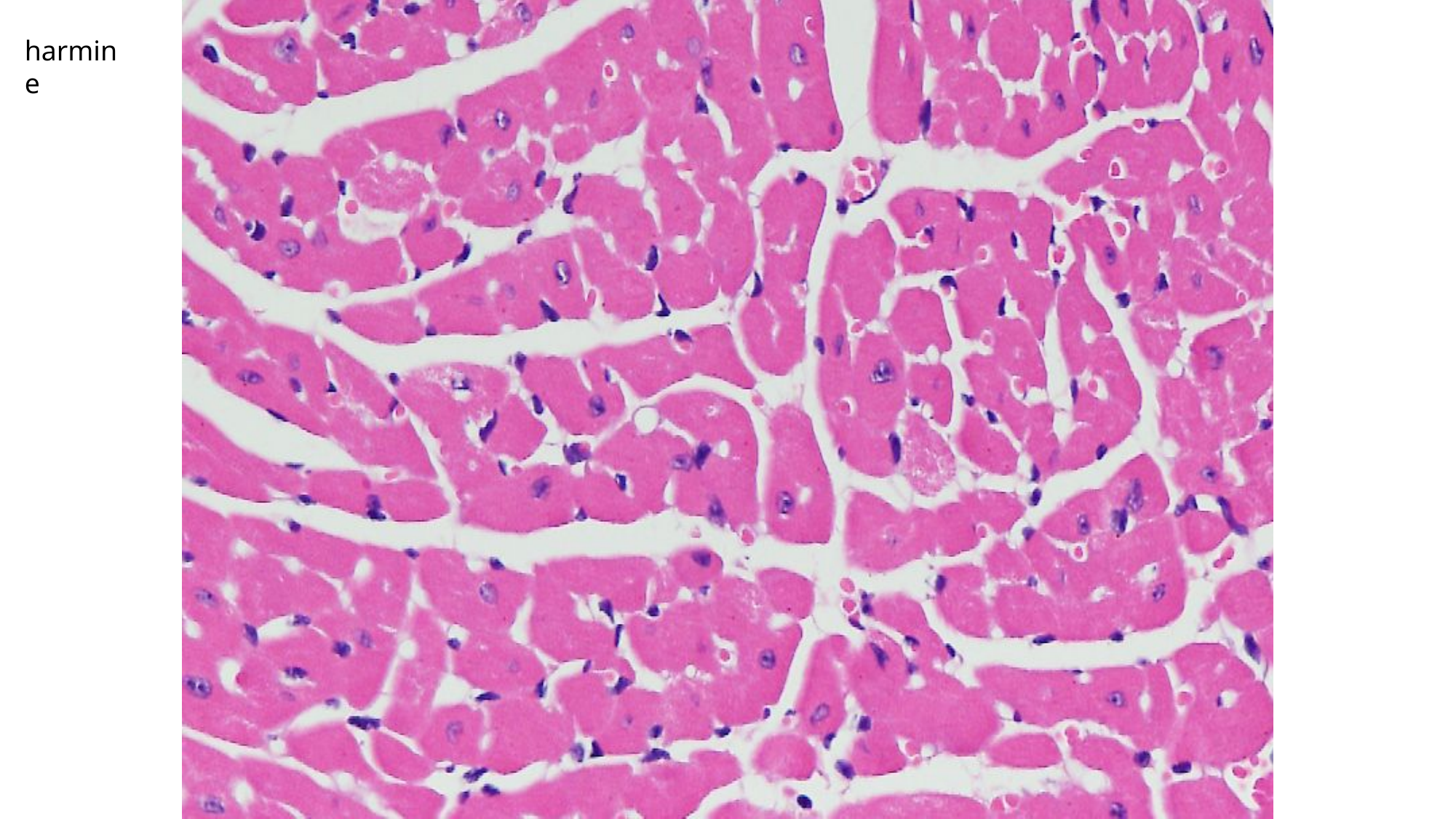

harmine

## Slide 17
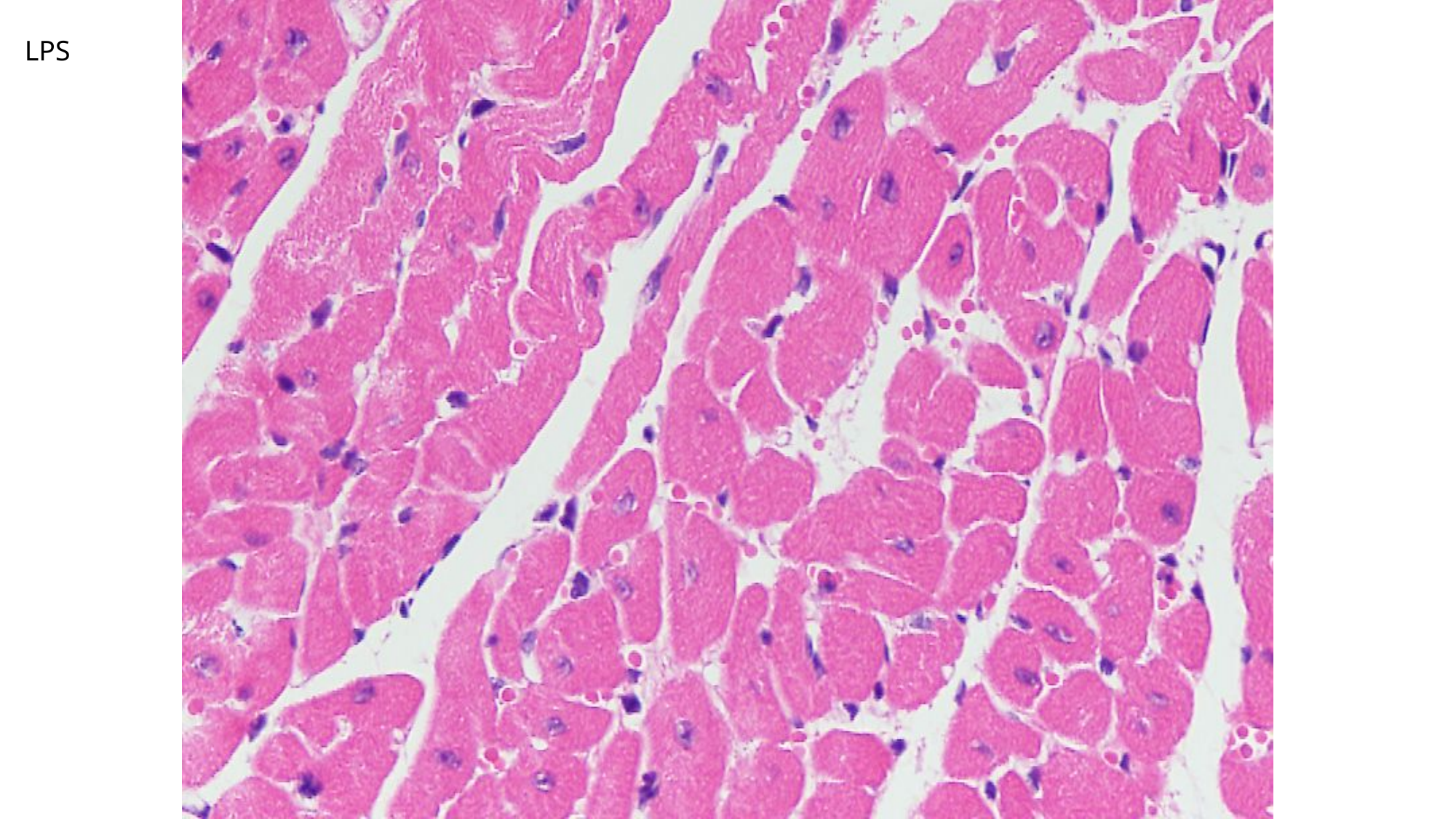

LPS

## Slide 18
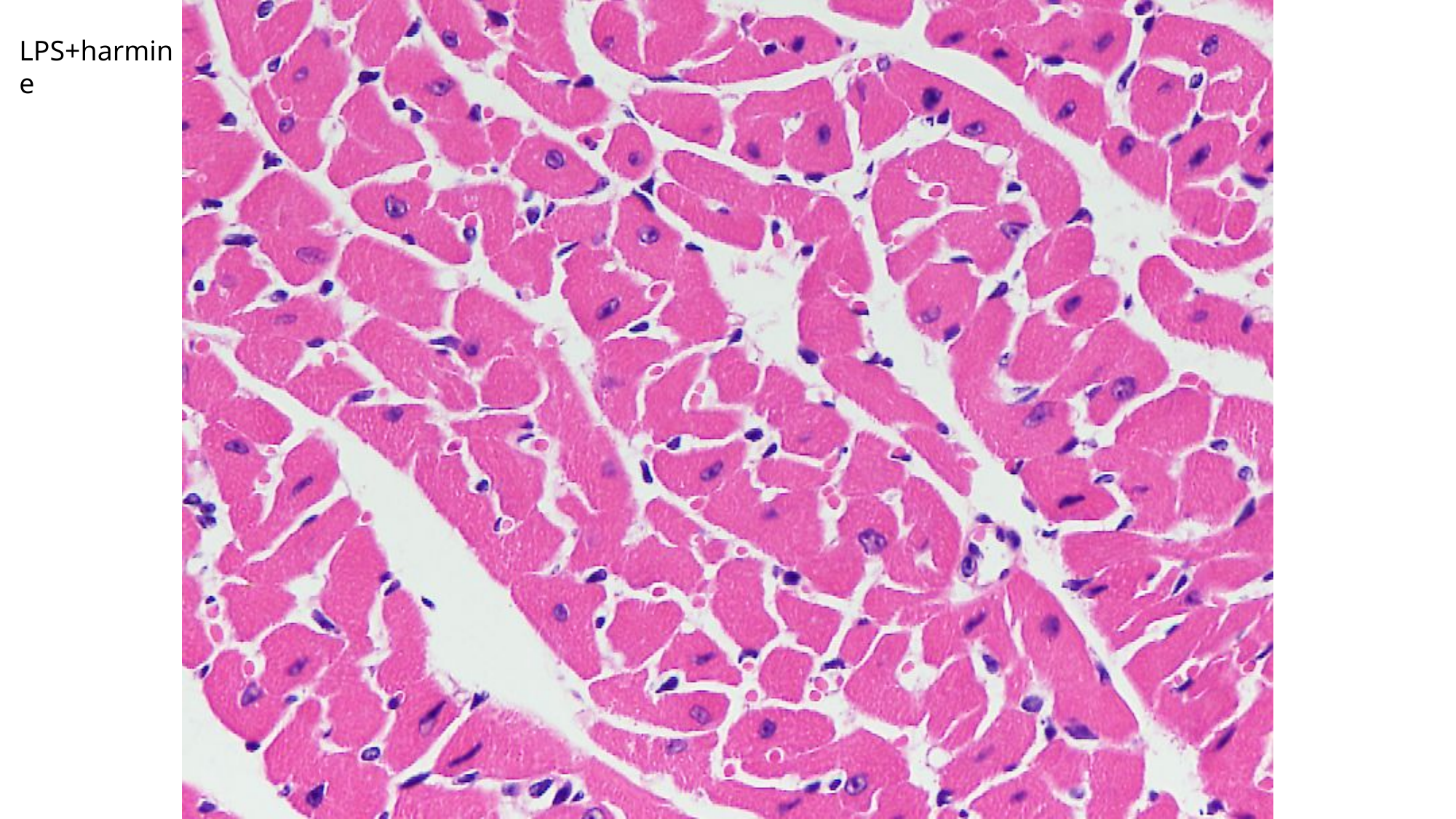

LPS+harmine

## Slide 19
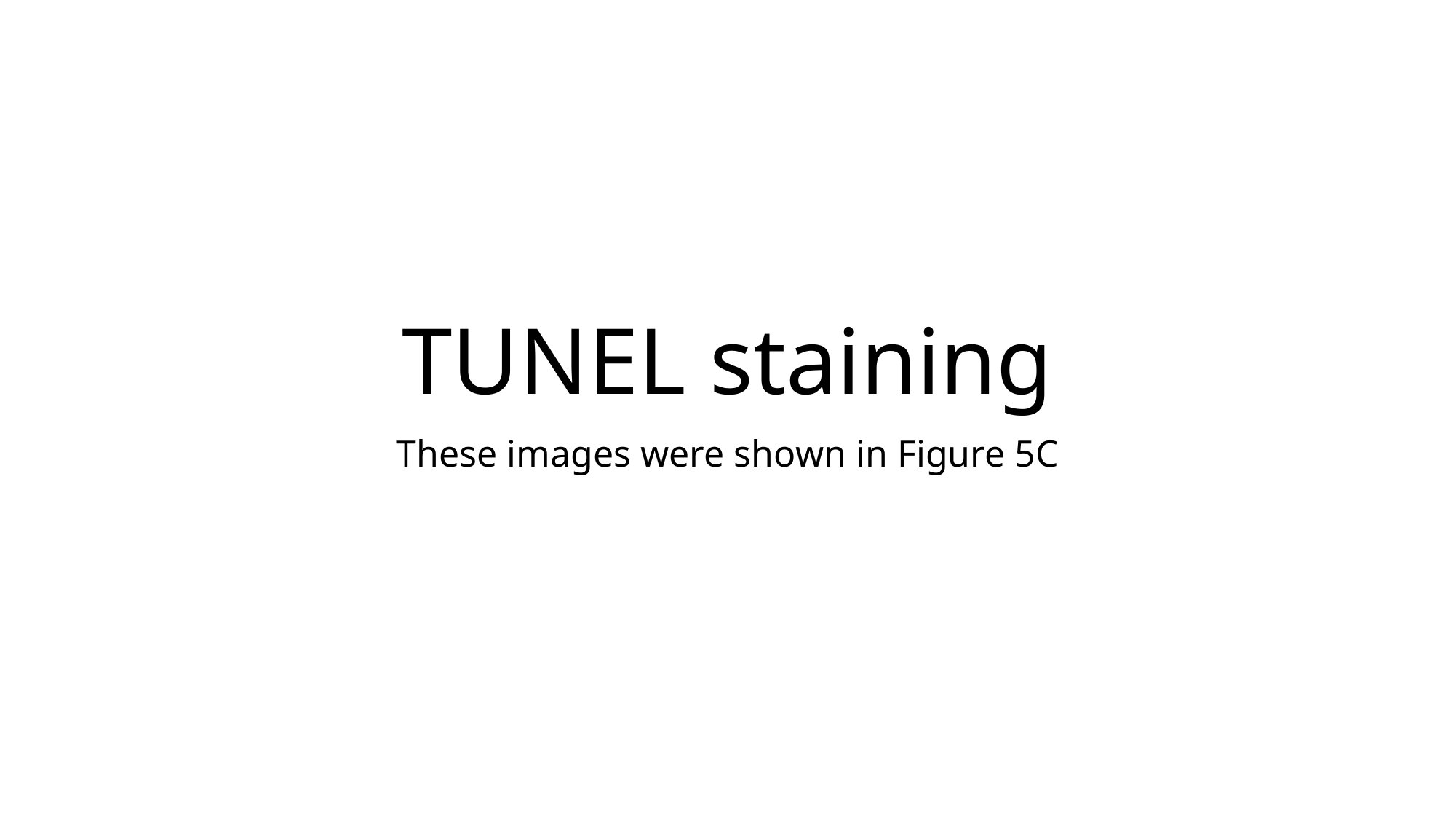

# TUNEL staining
These images were shown in Figure 5C

## Slide 20
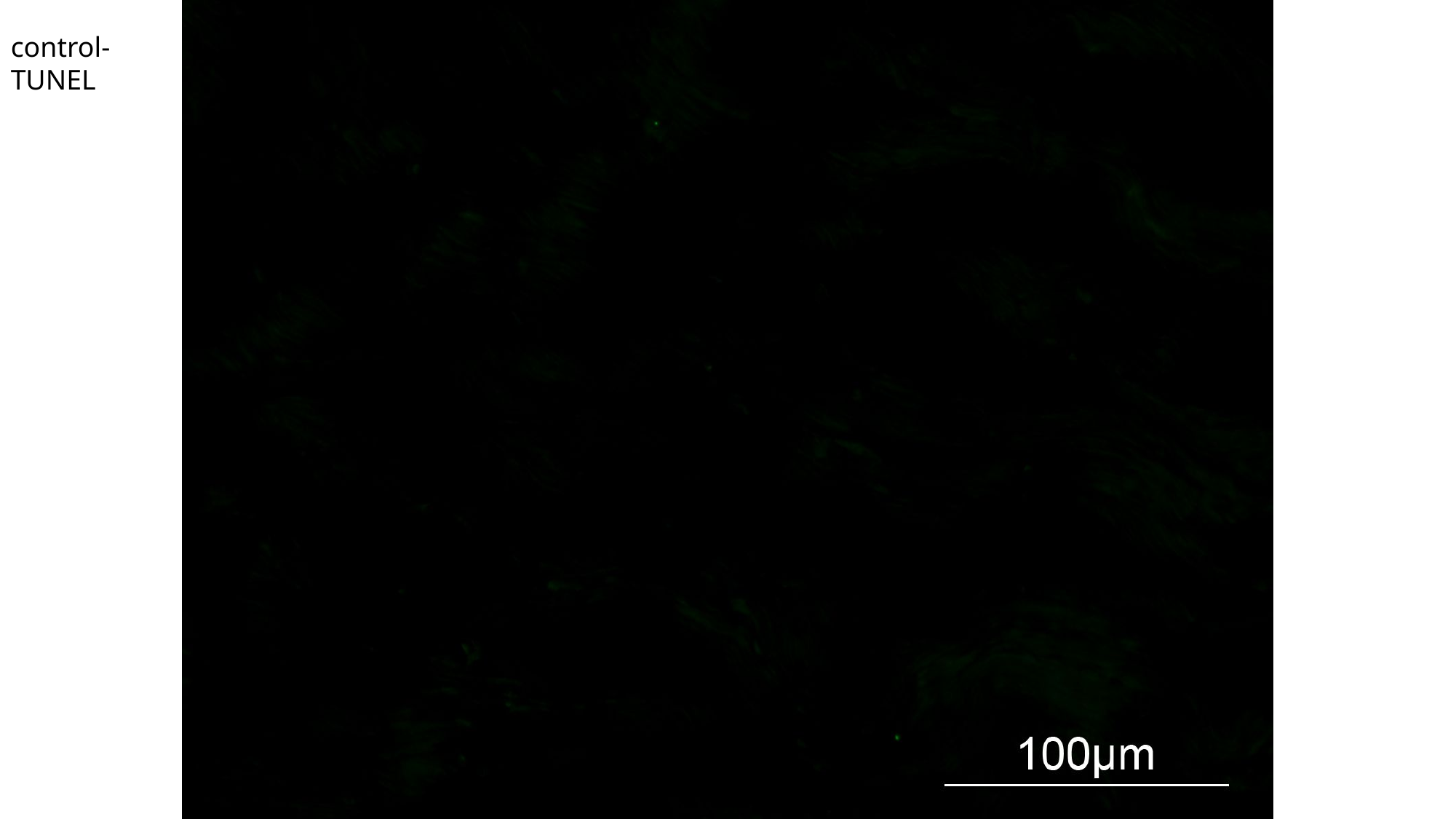

control-TUNEL

## Slide 21
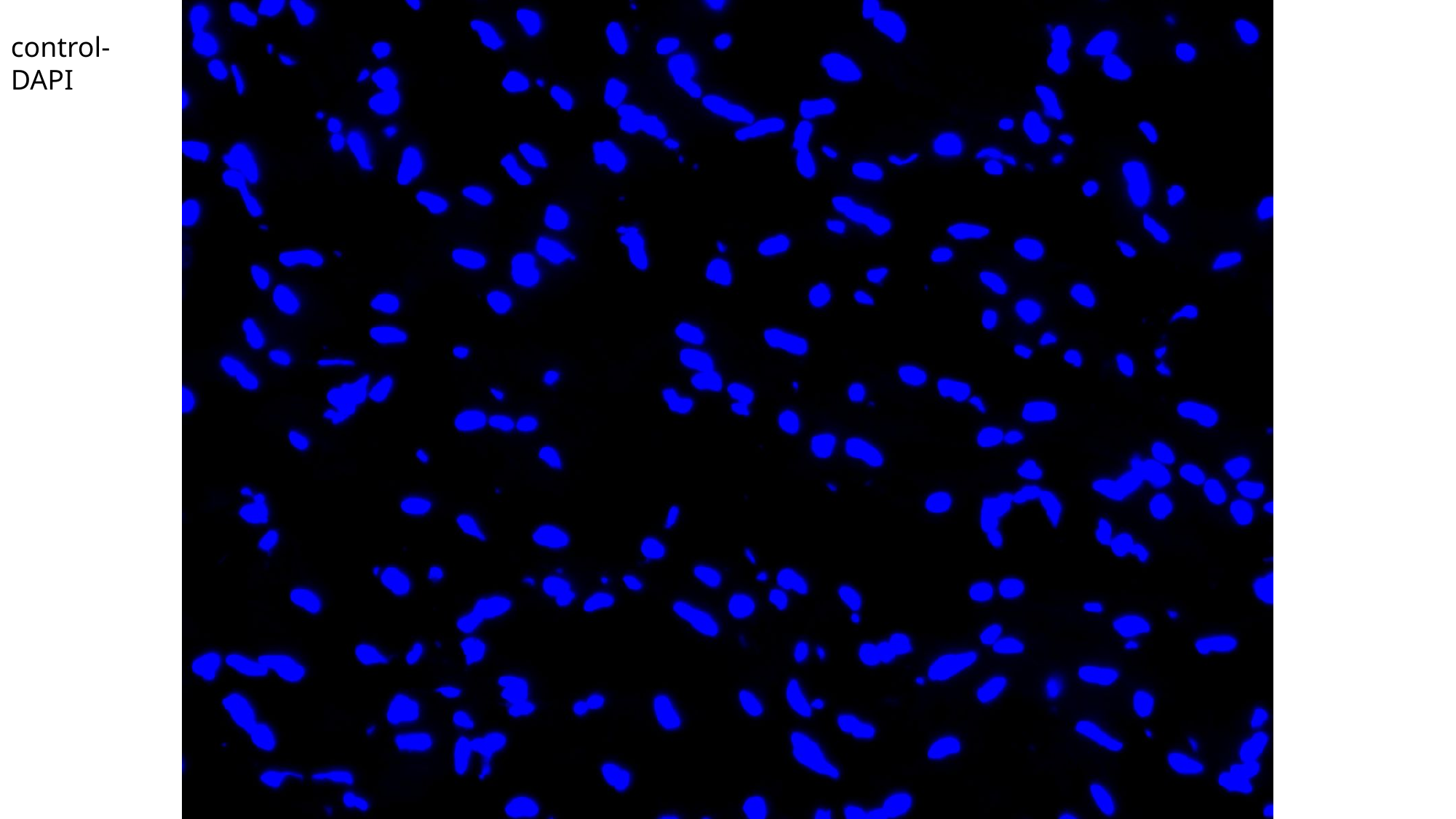

control-DAPI

## Slide 22
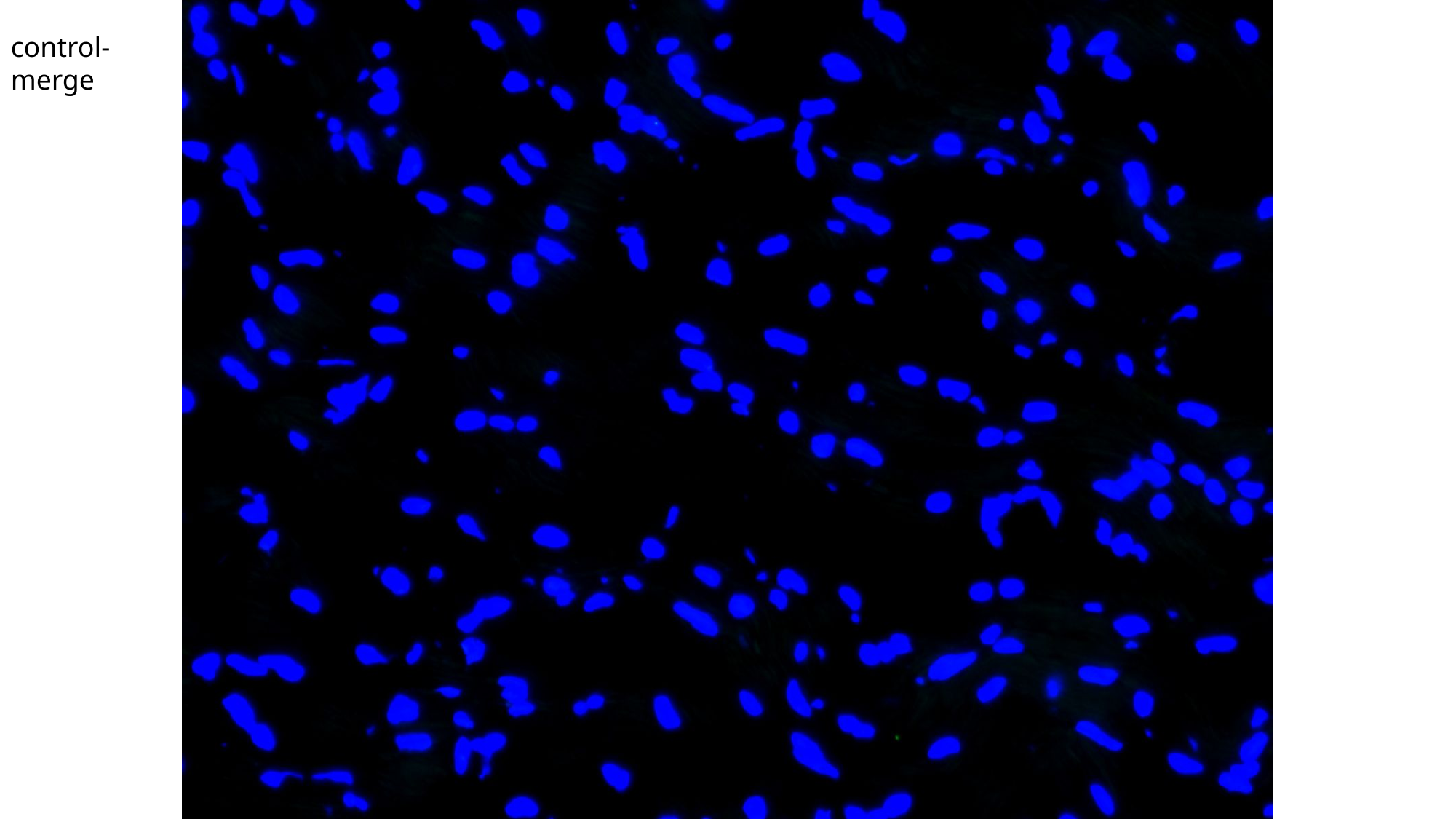

control-merge

## Slide 23
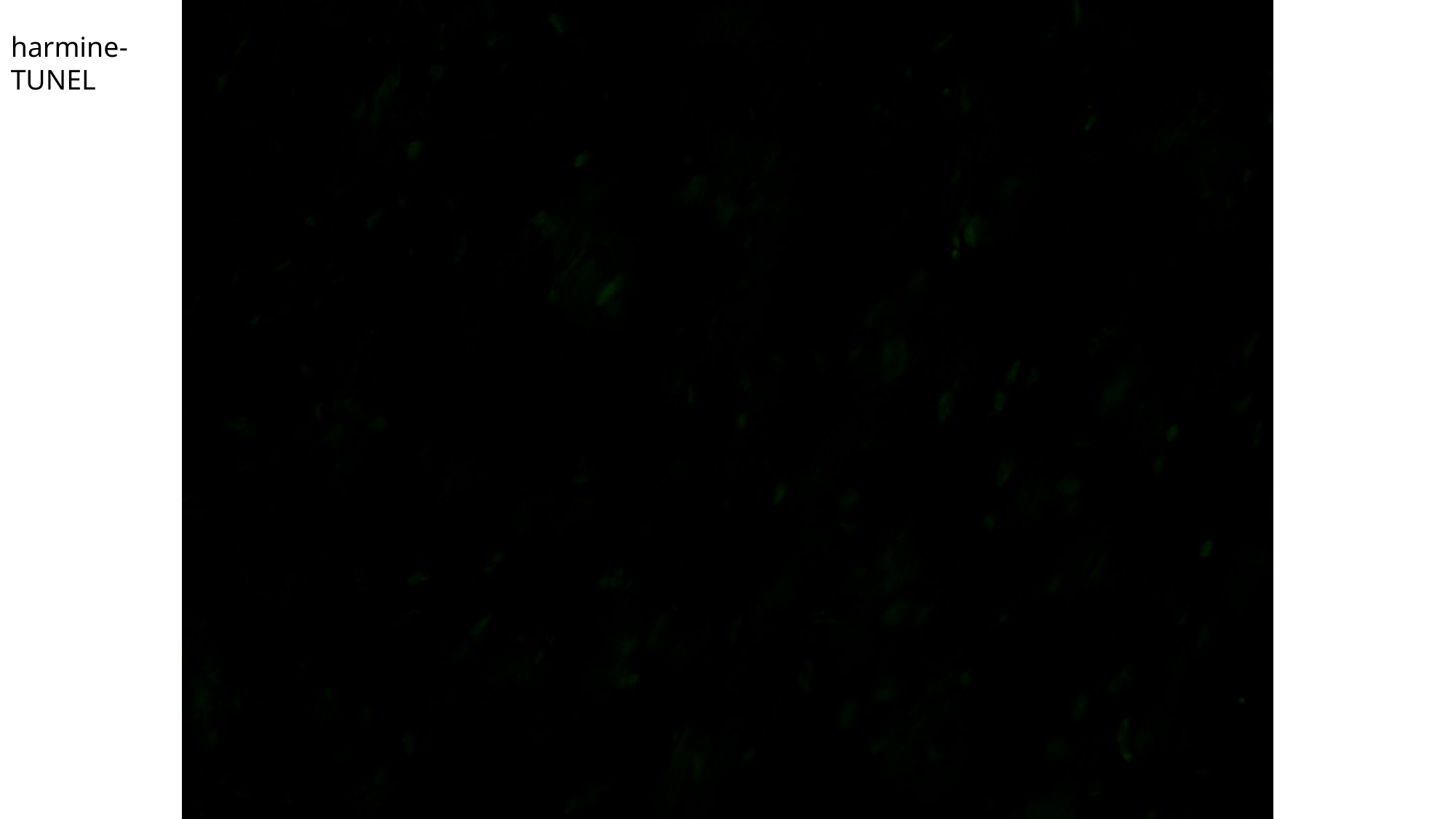

harmine-TUNEL

## Slide 24
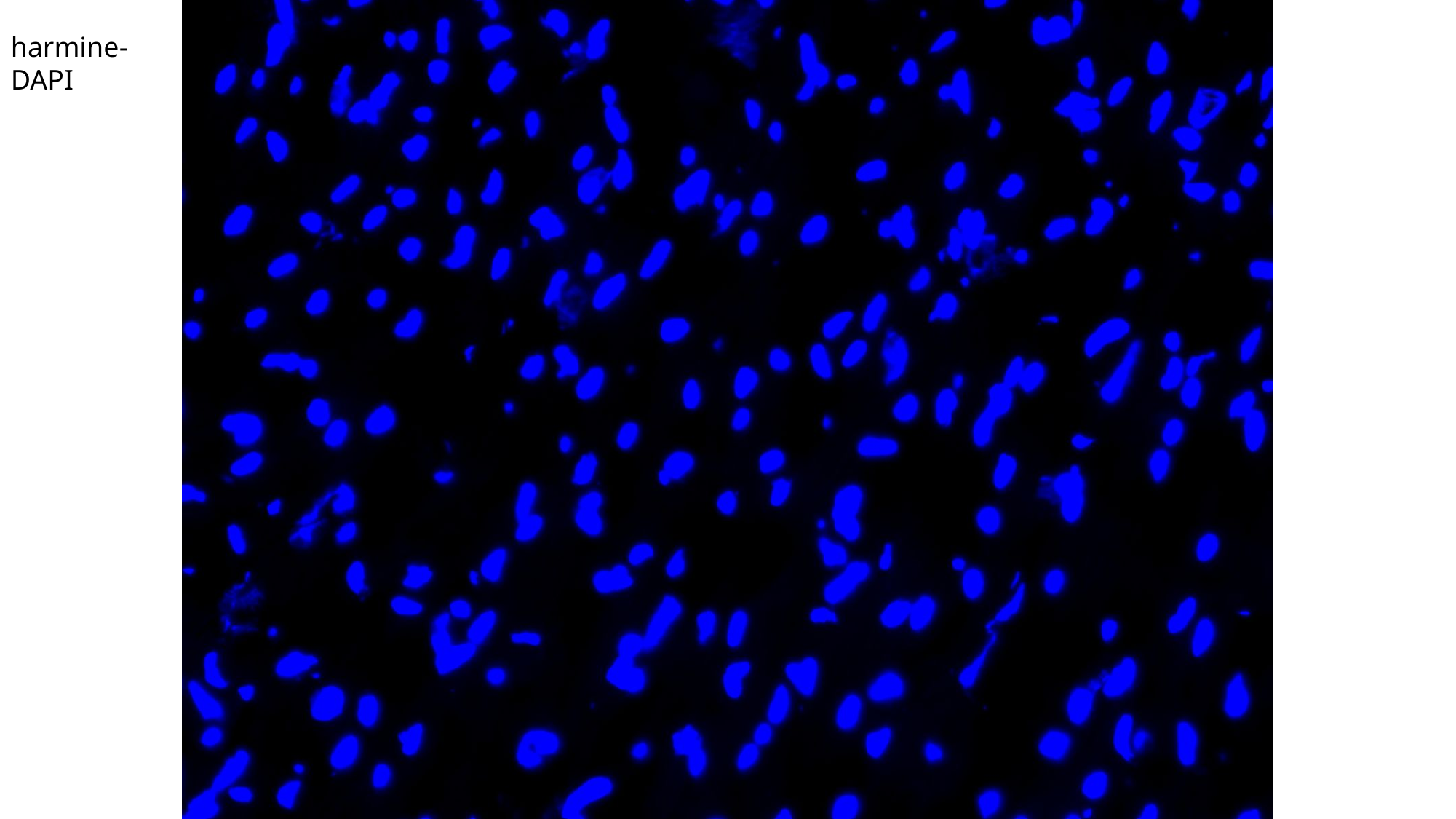

harmine-DAPI

## Slide 25
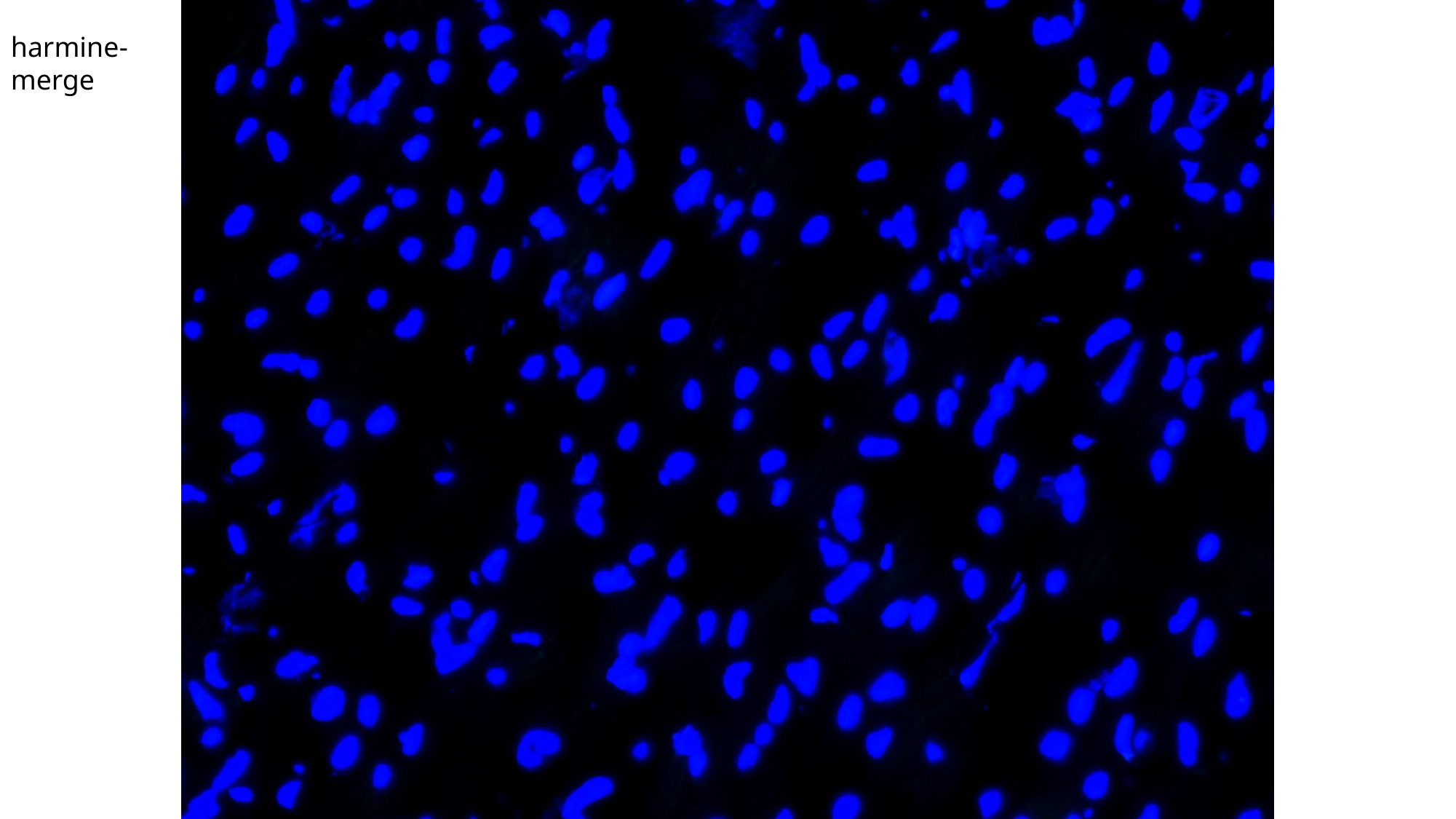

harmine-merge

## Slide 26
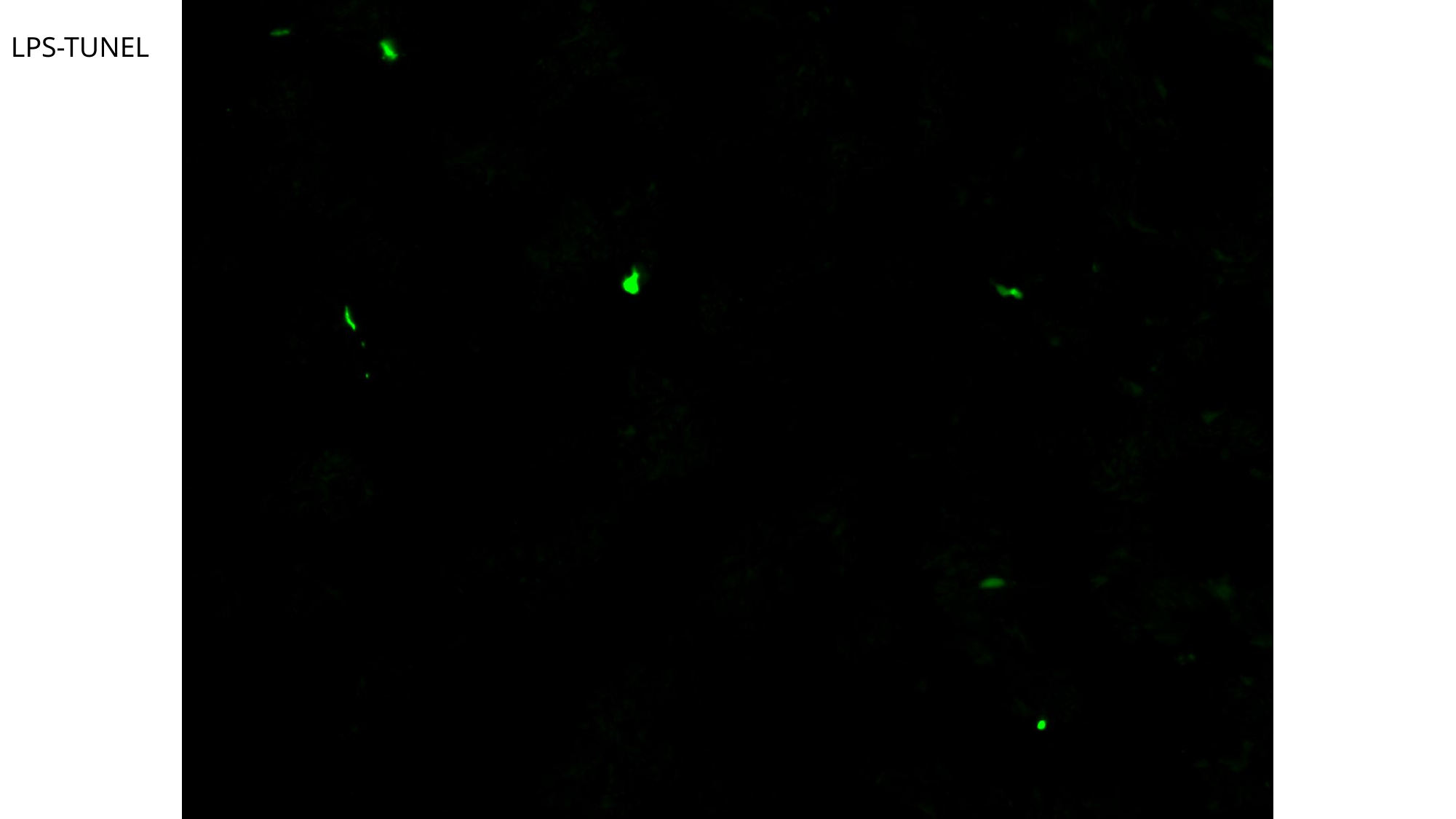

LPS-TUNEL

## Slide 27
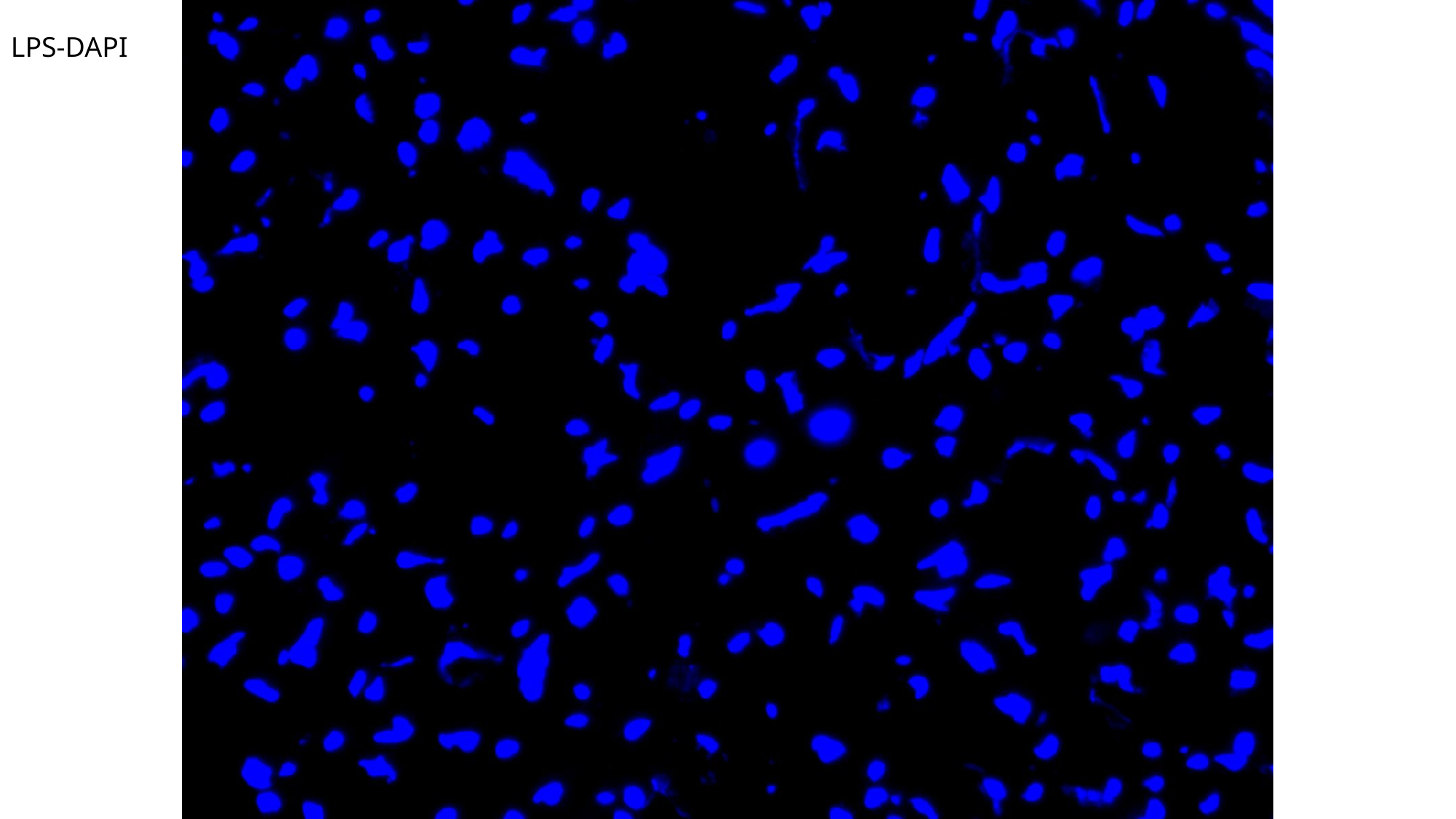

LPS-DAPI

## Slide 28
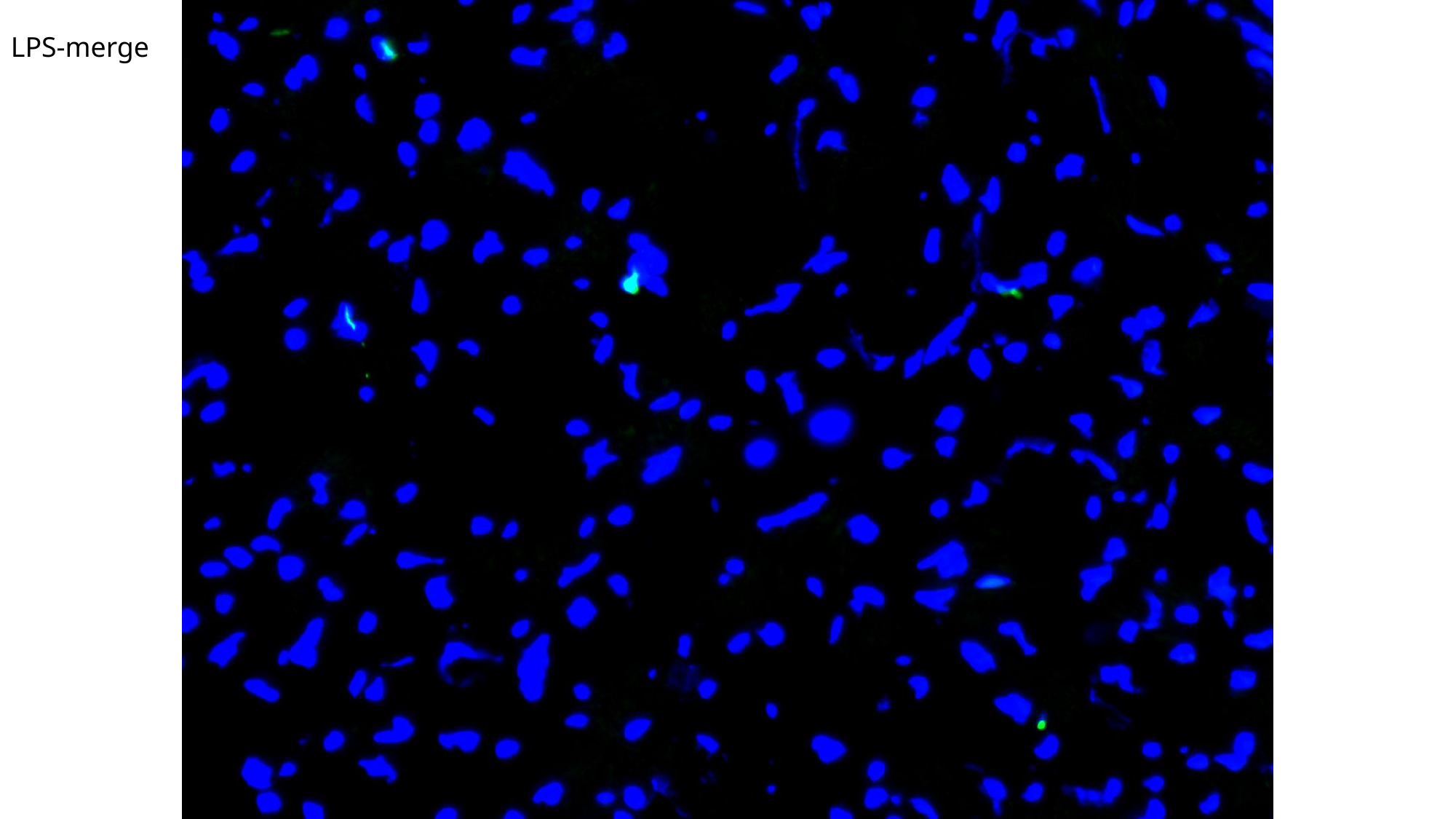

LPS-merge

## Slide 29
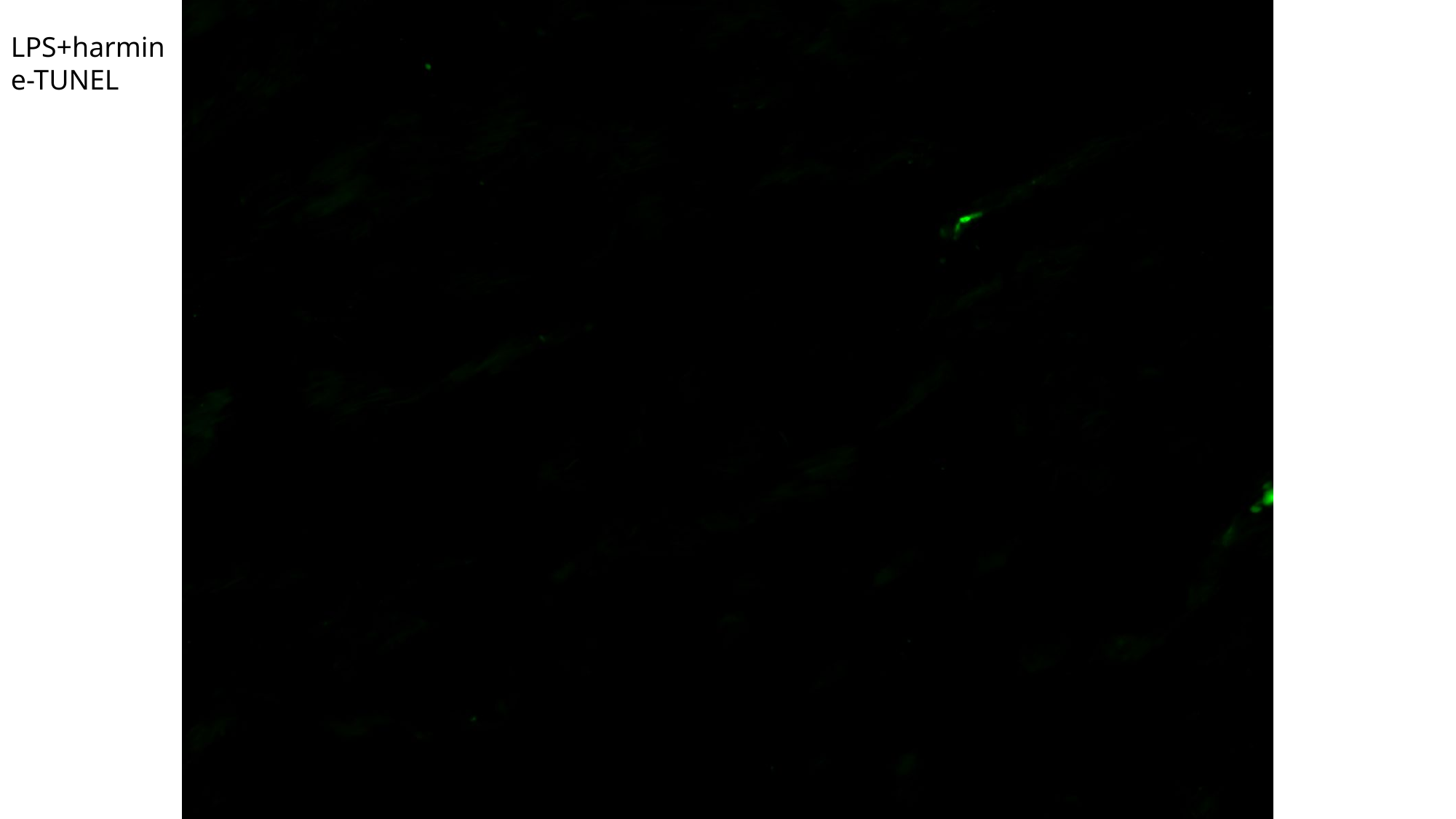

LPS+harmine-TUNEL

## Slide 30
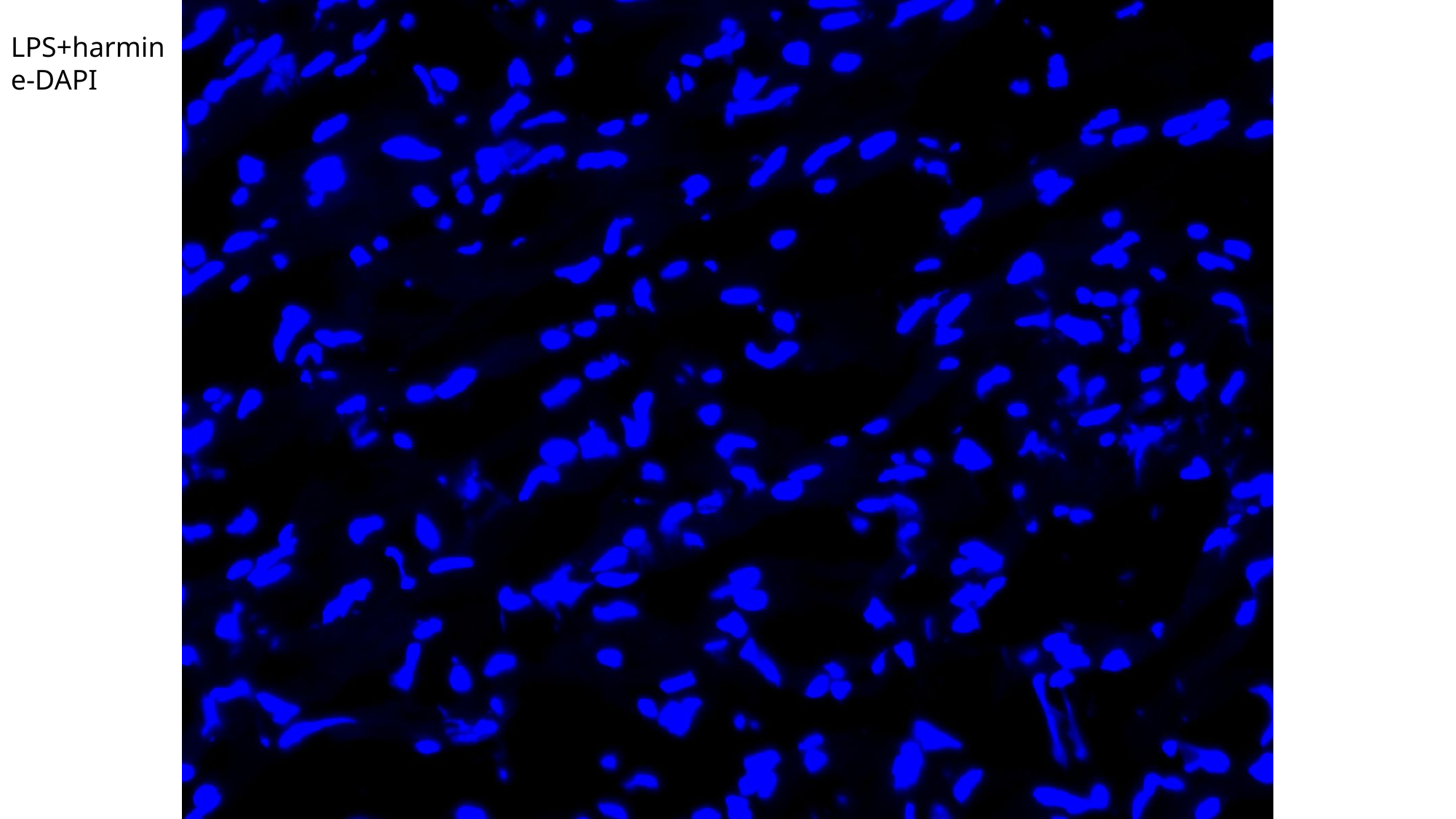

LPS+harmine-DAPI

## Slide 31
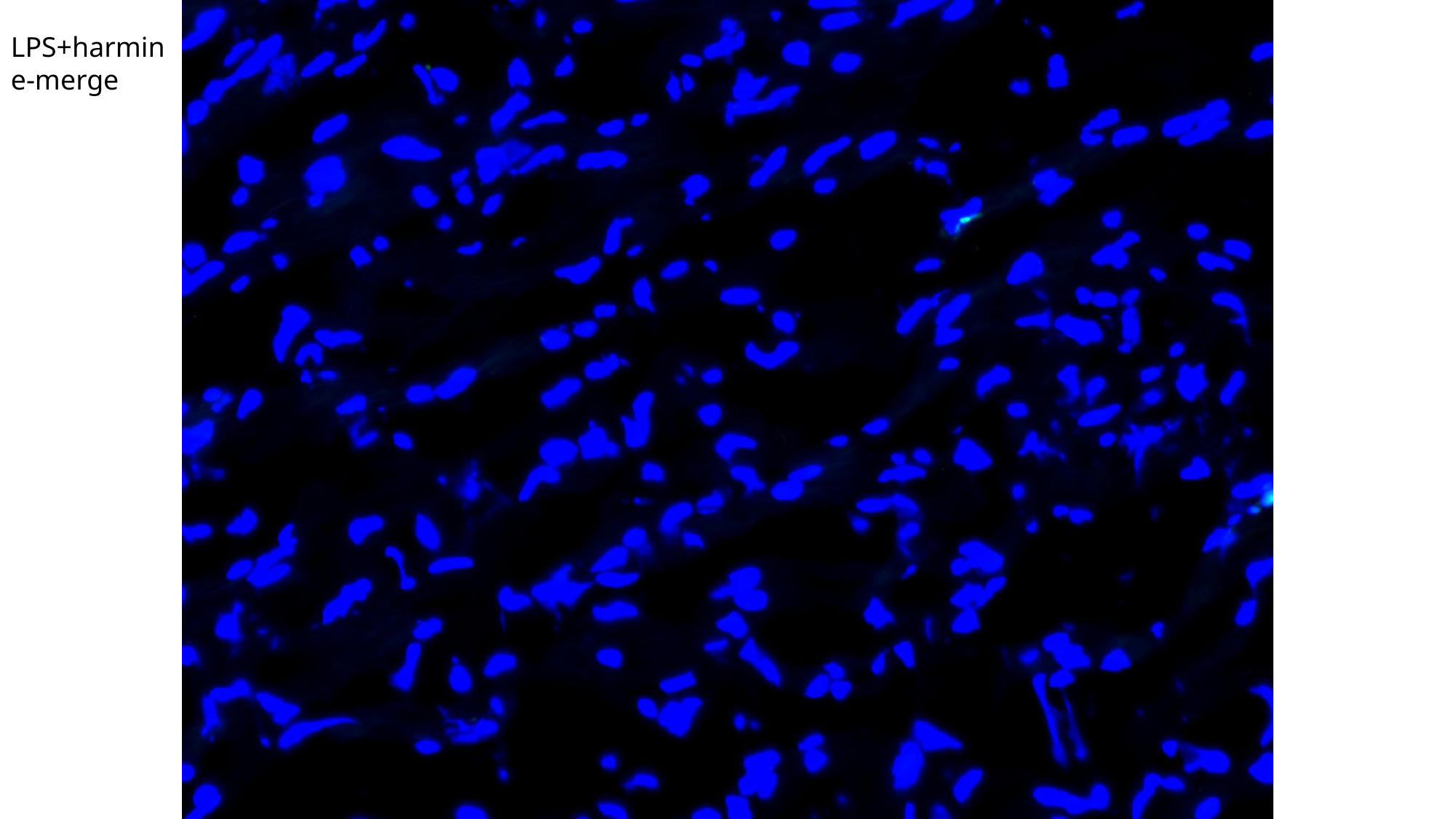

LPS+harmine-merge
