# Supplementary figures and images for: Harmine Alleviated Sepsis-Induced Cardiac Dysfunction by Modulating Macrophage Polarization via the STAT/MAPK/NF-κB Pathway
Source: Front Cell Dev Biol. 2022 Jan 17;9:792257. doi: 10.3389/fcell.2021.792257 (PMC8801946; doi:10.3389/fcell.2021.792257)

## Slide 1
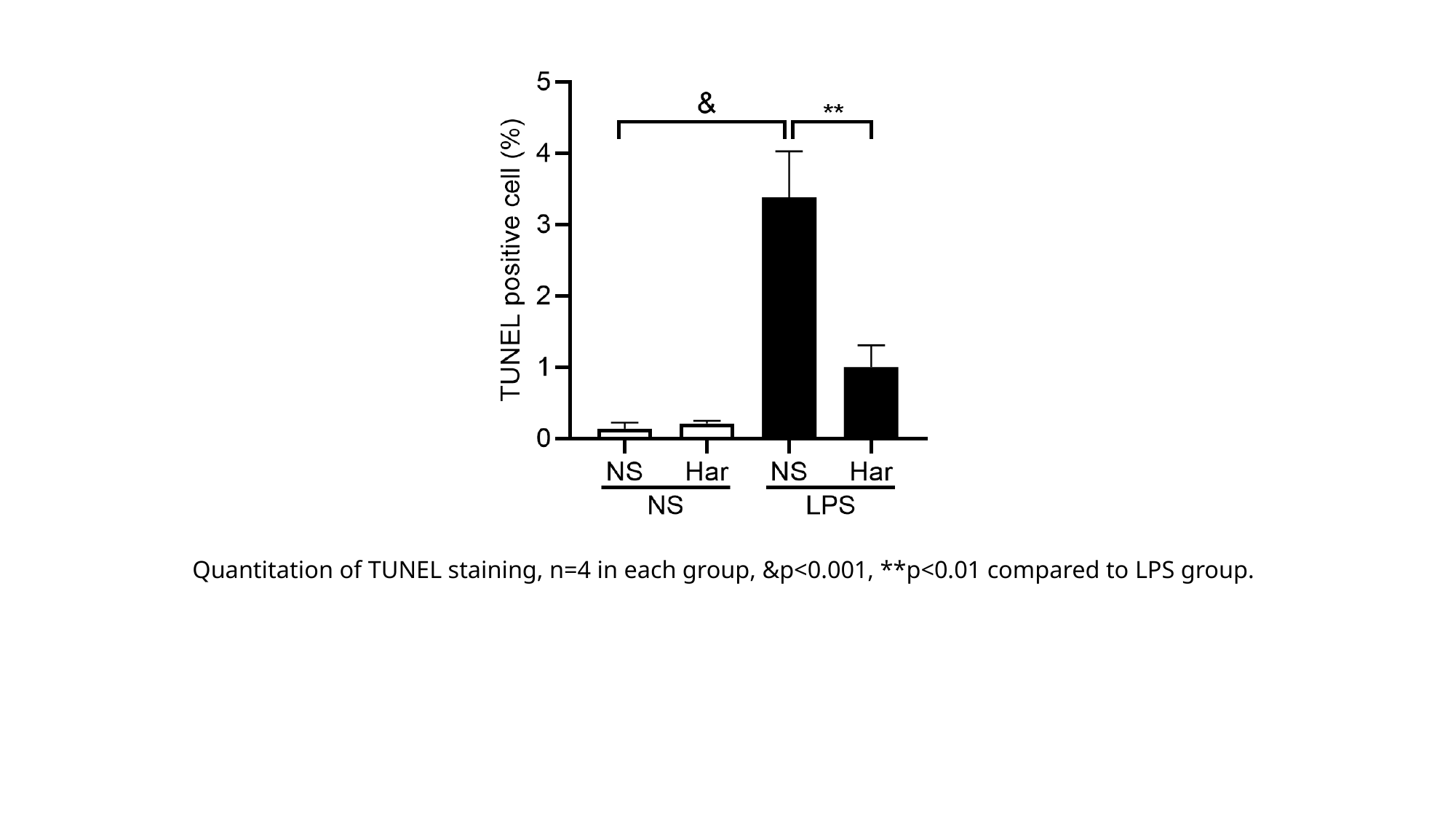

Quantitation of TUNEL staining, n=4 in each group, &p<0.001, **p<0.01 compared to LPS group.

Supplement: Supplementary file 6 [file Presentation7.PPTX]
